# Nanoplatforms for Sepsis Management: Rapid Detection/Warning, Pathogen Elimination and Restoring Immune Homeostasis

**DOI:** 10.1007/s40820-021-00598-3

**Published:** 2021-03-08

**Authors:** Gan Luo, Jue Zhang, Yaqi Sun, Ya Wang, Hanbin Wang, Baoli Cheng, Qiang Shu, Xiangming Fang

**Affiliations:** 1 Department of Anesthesiology and Intensive Care, The First Affiliated Hospital, School of Medicine, Zhejiang University, Hangzhou, 310003 People’s Republic of China; 2 National Clinical Research Center for Child Health, Children’s Hospital, School of Medicine, Zhejiang University, Hangzhou, 310052 People’s Republic of China

**Keywords:** Sepsis, Nanoplatform, Detection, Immune homeostasis

## Abstract

Sepsis, a highly life-threatening organ dysfunction caused by uncontrollable immune responses to infection, is a leading contributor to mortality in intensive care units. Sepsis-related deaths have been reported to account for 19.7% of all global deaths. However, no effective and specific therapeutic for clinical sepsis management is available due to the complex pathogenesis. Concurrently eliminating infections and restoring immune homeostasis are regarded as the core strategies to manage sepsis. Sophisticated nanoplatforms guided by supramolecular and medicinal chemistry, targeting infection and/or imbalanced immune responses, have emerged as potent tools to combat sepsis by supporting more accurate diagnosis and precision treatment. Nanoplatforms can overcome the barriers faced by clinical strategies, including delayed diagnosis, drug resistance and incapacity to manage immune disorders. Here, we present a comprehensive review highlighting the pathogenetic characteristics of sepsis and future therapeutic concepts, summarizing the progress of these well-designed nanoplatforms in sepsis management and discussing the ongoing challenges and perspectives regarding future potential therapies. Based on these state-of-the-art studies, this review will advance multidisciplinary collaboration and drive clinical translation to remedy sepsis.

## Highlights

- This review highlights pathogenesis and clinical challenges of sepsis.
- Advantages of different types of nanoplatforms are presented, and the rationality of nanoplatforms in sepsis management is analyzed.
- Advances of nanoplatforms in diagnosis and therapy of sepsis are systematically summarized, and ongoing challenges and future perspectives are discussed.

## Introduction

**Fig. 1:**
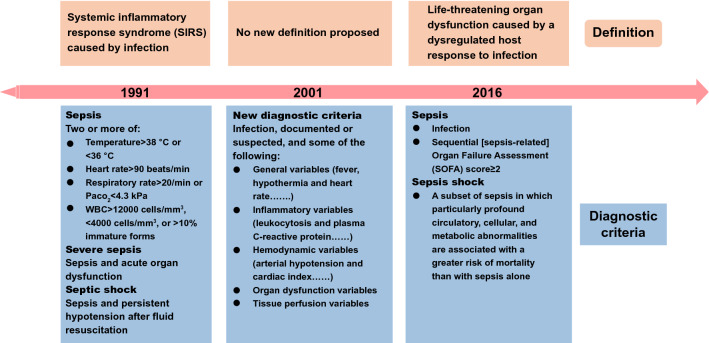
Definition variation of sepsis

Sepsis is defined as life-threatening organ dysfunction caused by a dysregulated host response to infection [1], which contributes the highest mortality to intensive care units (ICU) worldwide [2–4]. Although the term “sepsis” has already been purposed 2700 years ago, sepsis remained clinically undefined until the early 1990s [5]. As we can see in Fig. 1, sepsis definition experiences a historical variation from systemic inflammatory response syndrome (SIRS) to multiorgan dysfunction resulted from infection-caused abnormality in host response [6, 7]. Such a transition, driven by the improved understanding of pathophysiological mechanisms involved in sepsis development, thereby advances the diagnostic criteria and therapeutic principles [7], which represents the significant guideline for developing advanced diagnostic technologies and therapeutic agents. As a common syndrome in clinical intensive care, sepsis remains the leading cause of death among critically ill patients worldwide [2]. In 2001, the incidence of severe sepsis in America was more than 750,000 per year, with 300 cases per 100,000 population [3], contributing to at least one-third of all in-hospital deaths [8]. Additionally, patients with sepsis in the UK occupy approximately 27% of all ICU beds [4, 9]. Nevertheless, a considerable number of septic patients remain outside the ICU due to unbalanced medical resources and economic development in different countries [4]. Although sepsis is a global priority, basic medical research and clinical evidence from low-income countries are poor [10]. Sepsis is also an expensive and frequently fatal syndrome in critically ill surgical patients in China [11]. As early as 2007, an epidemiological study conducted by our group found that the overall hospital mortality of severe sepsis in China had already reached 48.7%, leading to high hospital costs of $11,390 per patient and $502 per patient per day [11]. Although numerous efforts have been made, sepsis continues to impose a heavy burden and high healthcare risk, and related deaths are reported to account for 19.7% of all global deaths [12]. Consequently, there is an urgent clinical need to efficaciously manage sepsis for both developed and developing countries.

**Fig. 2:**
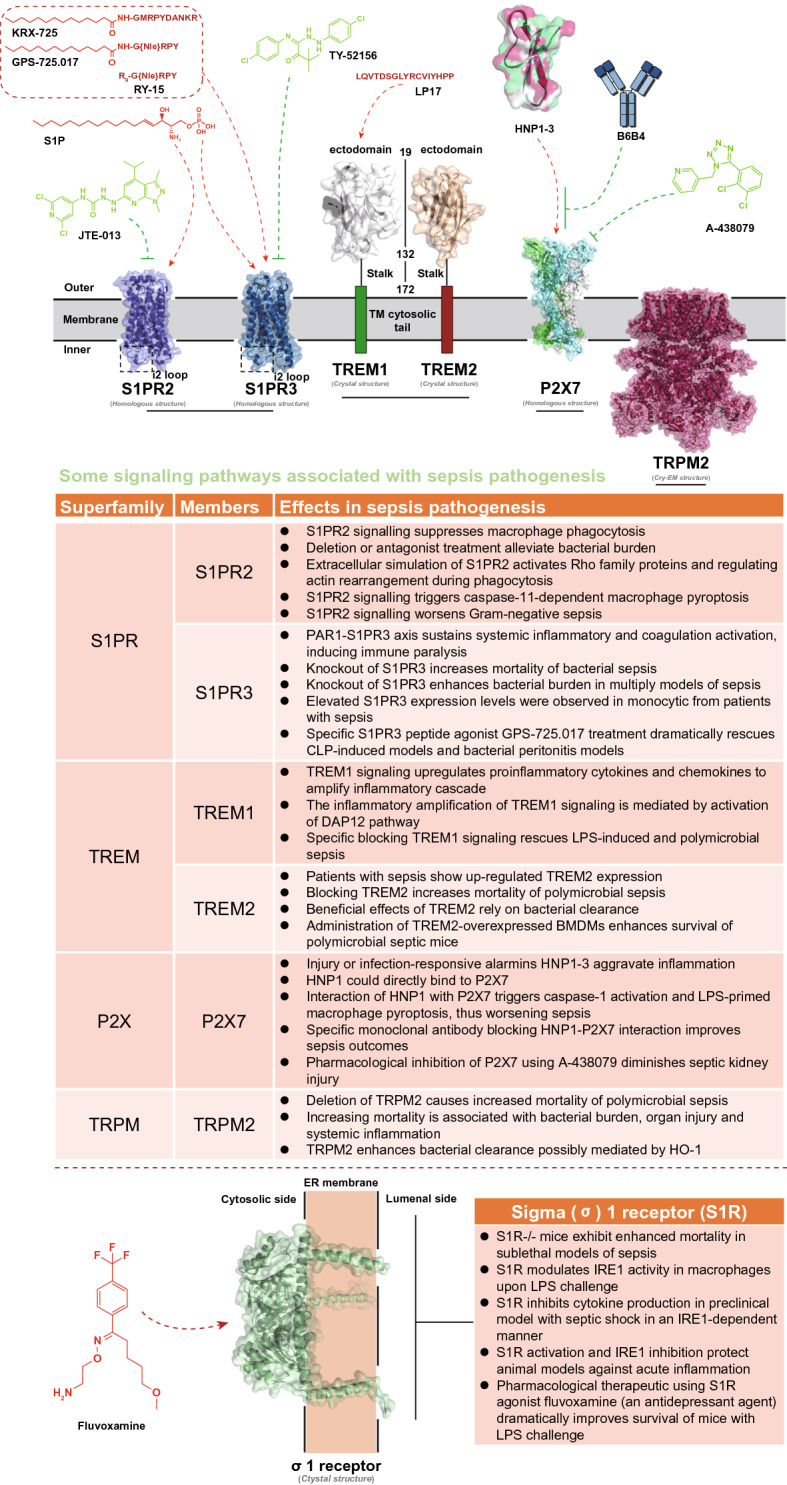
Brief summary of some novel molecular pathways and their potential therapeutic applications

Although an increasing number of the mechanisms involved in sepsis pathogenesis have been elucidated, the complicated alterations in pathophysiology still cause delayed diagnosis and therapeutic failure [13]. The pathogens, with their pathogen-associated molecular patterns (PAMPs), such as lipopolysaccharide (LPS), trigger the activation of innate immune systems to defend and eliminate invaders [14]. However, the invaders sometimes prevail, and the immune responses fail to return to homeostasis [13]. The resultant immune disorder further generates a series of damage-associated molecular patterns (DAMPs) in response to tissue injury and cell death (e.g., endothelial pyroptosis), leading to sustained immune disorder and organ dysfunctions [13, 15]. In the early stage of sepsis processing, large accumulation of cytokines resulted from excessive proinflammatory cascades critically raises risk of multiorgan failure, which also complicates with many other pathological events including complement activation, coagulation and endothelial dysfunction as well as the generation of neutrophil extracellular traps (NETs) [13, 15, 16]. After proinflammatory storm, the imbalanced immune system tends to undergo suppression that is associated with lymphocyte exhaustion and the reprogramming of antigen-presenting cells [10, 13, 17]. In this case, the occurrence of LPS tolerance causes “immune paralysis” with diminished proinflammatory cytokine release upon exposure to PAMPs and DAMPs [13, 17]. Immune suppression in turn results in reduced elimination of infection, leading to the uncontrollable growth of pathogens, which ultimately worsens sepsis outcomes. Consequently, multipathway therapeutics that favorably restores immune homeostasis are urgently needed, owing to the complexity of pathogenesis. Clinically available strategies such as antibiotic treatment, hemodynamic maintenance, and organ support can eliminate causative agents and maintain functions necessary for life; however, these approaches are inadequate to resolve immune disorders and reverse the progress of organ failure [10, 18, 19]. Nevertheless, ongoing identification of pharmacologically valuable drug targets responsible for immune modulation, might contribute to the establishment of multipathway therapeutics in the future. For example, sphingosine 1-phosphate receptor (S1PR) family [20–23], triggering receptor expressed on myeloid cells (TREM) family [24, 25], ion channel P2X7 [26–28] and transient receptor potential melastatin 2 (TRPM2) [29–31], as well as the endoplasmic-reticulum resident transmembrane protein sigma-1 (σ1) receptor [32], have all been proven as pharmacologically acceptable targets with significant influence to sepsis pathophysiology and the final outcomes (Fig. 2) [33]. By using corresponding agonists or antagonists, survival of mice with sepsis can be certainly improved (Fig. 2), thereby lightening the future therapeutic prospects in clinical sepsis management. Currently, antibiotics still represent the most irreplaceable strategy, due to the critical necessity for sepsis management to rapidly control pathogenic sources. The Survival Sepsis Campaign Guidelines strongly recommended antibiotic treatment following prompt identification of sepsis [34]. Of further note, the rapid completion of a 3-h bundle of sepsis care and the rapid administration of antibiotics contribute to lower risk-adjusted in-hospital mortality [35]. Unfortunately, the emergence of antibiotic resistance represents a tremendous threat to sepsis treatment, especially in low-income countries, because of the overuse of antimicrobials [36]. Hence, there is an urgent need to develop next-generation antibiotics and/or novel therapeutic agents as alternative strategies, which usually require multidisciplinary cooperation by medicinal chemists, clinicians, material chemists, and biomedical scientists.

Nanomedicine has aroused increasing attention in recent decades because of its unique advantages in improving therapeutic efficiency. Intriguingly, nanotechnology was already being utilized in the development of molecular machinery and in medical investigations in 1977 [37]. Since then, medical investigators have gradually realized that nanotechnology might contribute potential advances to basic medical research and clinical practice, generating the “nanomedicine” field [38]. Nanomedicine can be achieved by employing self-assembled nanomaterials as drug carriers or reprogramming drug structures using supramolecular chemistry to achieve nanoassembly. The former potentiates targeted drug delivery and/or provides favorable biodistribution and/or bioavailability or release behaviors for cargo molecules by using nanocarriers [39]. Liposomes, polymeric micelles and nanoemulsions all belong to these nanomaterial-inspired delivery systems that have been used in the therapy of various diseases [39–42]. Liposomal doxorubicin has been approved by the FDA for the treatment of HIV-related Kaposi sarcoma [43]. The polymeric micelle paclitaxel was approved in Korea for the treatment of breast cancer and NSCLC [43]. In addition to systemic administration, bacterial surface protein-functionalized liposomes potentially promote the oral delivery of biomacromolecules (*e.g.,* vaccines) by enhancing gastrointestinal adhesion and physicochemical stability [44, 45]. Additionally, nanoemulsions have widely been employed to overcome poor solubility, thus improving the bioavailability of cargo drugs, particularly the biopharmaceutical classification system (BCS) II drugs [46]. In contrast, drug structure-guided nanomedicine directly manipulates supramolecular chemistry to reprogram the chemical structures of target drugs by analyzing the structure–activity relationship, thereby obtaining nanoassembly behaviors that in turn contribute to improved drug properties such as safety and pharmacokinetics [47]. Wang et al. [48] synthesized linoleiclyated SN-38 prodrugs that self-assemble into nanomicelles, contributing to antitumor efficacy by improved safety and enhanced permeability and retention (EPR) effects. After coassembly with the iRGD-LA conjugate, the nanoprodrug shows more favorable targeting properties [26]. Moreover, hydrophilic chain oligolactide-engineered cytotoxic cabazitaxel coassembled with PEG-*b*-PLA represents an adaptive nanoparticle platform for improved drug safety and therapeutic efficacy [49]. In addition to supramolecular chemical nanomedicines, some inorganic nanomedicines (e.g., nano-Au and nano-Ag) are popular in cancer therapy and antibiotic development [50–52]. Nanomedicine has also been applied in other disease treatments, including diabetes and atherosclerosis [41, 42].

With regard to sepsis management, although effective and specific therapeutics remain unavailable, the emergence of nanoplatforms targeting septic microenvironments (e.g., pathogens, imbalanced host responses, and specific biomarkers) presents a novel avenue for assisting accurate diagnosis and precision treatment [38]. As a common strategy for drug formulation, nanomedicine potentially eliminates sepsis-associated pathogens and/or targets the restoration of immune homeostasis [53]. With the in-depth elucidation of pathogenesis, the cross talk between exogenous threats and endogenous molecular signaling networks has gradually been mapped clearly [13, 54], which will guide the rational design of sophisticated nanoplatforms for adaptive sepsis management. Nanoparticle antibiotics (termed nanobiotics) are designed to disrupt antibiotic-resistant bacteria, aiming to overcome the antibiotic overuse-induced poor prognosis in the late phase of sepsis [55]. Additionally, nanomedicine to neutralize bacterial endotoxin will hopefully become an alternative strategy to diminish the activation of proinflammatory pathways [56, 57]. To avoid the excessive inflammation involved in sepsis development, biomimetic nanotherapeutics targeting immune cells or endothelial cells were created. Nevertheless, numerous efforts should still be made to explore more adaptive nanoplatforms for sepsis management [38]. This review will focus on three topics: (1) analyzing the rationality of nanoplatforms in sepsis management, in which we give a general framework of different types of nanoarchitectures and subsequently ask why are nanoplatforms desirable for sepsis management and present our comments; (2) a summary of recent advances in nanotechnology, specifically the construction of nanodiagnostic platforms to achieve rapid, accurate, and/or real-time detection of sepsis-associated biomarkers; and (3) recent advances in nanotherapeutic platforms constructed from supramolecular nanomaterials and/or biomimetic biomaterials that remedy sepsis through eliminating bacterial infections and/or restoring immune homeostasis. We will also discuss future perspectives and ongoing challenges in the continued development of this field.

## Rationality of Nanoplatforms in Sepsis Management

Rapid evolution and progression of nanomaterials allow to fabricate diversiform nanoparticles (NPs) (e.g., inorganic/metallic NPs, polymeric NPs, liposomes, and biomimetic NPs) capable of constructing sophisticated nanoplatforms which are integrated with diagnostic and/or therapeutic functions to manage various diseases [58, 59]. Superior to conventional or microscale chemical/biological materials, nanomaterials present a series of unique characteristics, including optical fine-tuning, magnetism, potent surface energy, biotunable surface chemistry, fine-tunable size and surface potential, and/or in vivo passive and active targeting [60, 61]. By taking advantage of these clinically adaptive abilities, increasing numbers of nanoplatforms have been constructed to advance the development of diagnostic/therapeutic techniques in various diseases (e.g., cancer [62–65], infectious diseases [66], inflammatory diseases [67], cardiovascular diseases [59], and neurodegenerative diseases [68]) through the rational/targeted delivery of diagnostic and/or therapeutic agents, improved bioavailability and biodistribution, maximizing the theranostic efficiency of cargo agents, and/or inherent photomagnetic effects [59].

### Brief Introduction to Different Types of NPs for Establishing Nanoplatforms

Nanoarchitectures represent the most important elements in constructing nanoplatforms. By adopting different types and formulations of nanomaterials, controlling nanoassembly manners, as well as skillfully manipulating surface chemistry, multifaceted nanoarchitectures with diverse physicochemical and biological features (e.g., shape, size, surface potential, biodistribution, release profile, biocompatibility and biodegradation) can be established to construct personalized diseases-guided nanoplatforms for diagnostic and/or therapeutic applications, such as metallic/inorganic nanoparticles (NPs), liposomes, biomimetic NPs, and polymeric NPs.

#### Metallic/Inorganic NPs

Due to their fine-tunable properties, metallic/inorganic NPs have been widely investigated for diagnosis and/or therapy of various diseases (e.g., cancer [69], infectious diseases [70], inflammatory diseases [71], and wound care [72]), including gold NPs (AuNPs), silver NPs (AgNPs), copper NPs (CuNPs), metal oxides NPs, graphene oxide-based NPs, and mesoporous silica (mSiO_2_) NPs, etc*.* [73, 74]. Due to their high surface area-to-volume ratios and fine-tunable surface engineering, metallic/inorganic NPs, especially the metal NPs, can immobilize and sufficiently display diagnostic molecules, thus achieving signal amplification and improvements in sensitivity of molecular detection [75]. Nan Wang and coworkers adopted AuNPs as solid support to surface display copper ions, resulting in a copper ions-mediated AuNPs aggregate (Cu/Au NA) that senses bacterial endotoxin LPS through charge interactions and contributes to signal amplification and trace determination [76]. Besides, some metallic NPs, especially the nanoscale noble metals (e.g., AuNPs), inherently exhibit nanoplasmonic phenomenon such as localized surface plasmon resonance (LSPR) when expose to light, which subsequently induces strong absorption and scattering natures with excitation of visible to near-infrared wavelength range [75]. These spectral properties are sensitive to the variations in refractive index resulted from molecular adsorption onto these plasmonic nanoparticles and thus can be considered as specific signals for molecular detection [75]. Gold nanorods (GNR), a widely used nanoplasmonic materials, have recently been introduced to establish aptamer-based LSPR nanosensor that presents more simplified and ultrasensitive manner for serum extracellular domain of human epithermal growth factor receptor 2 (ECD-HER2) quantification than clinical methods, thereby advancing accurate and real-time prediction of metastatic breast cancer prognosis [77]. Metallic/inorganic NPs also show considerable superiorities as delivery platforms, including high drug loading capability, biotunable targeted properties, long circulation, on-demand release, and low immunogenicity, etc*.* [74]. Integrating multiple types of metallic/inorganic NPs into one unit enables synergistic enhancement in rational drug delivery, and consequently overcomes the limitations of conventional drug delivery platforms. Tran and coworkers fabricated a core–shell magnetic mSiO_2_ NPs that comprise a Fe_3_O_4_ core and a mSiO_2_ coating with graft of fluorescent conjugates [78]. After drug loading and surface coating of polydopamine, they found further wrapping with graphene oxide layer and subsequent antibody functionalization exhibit more stable release behavior, active targeting feature, and dual stimuli-response to pH and near-infrared radiation (NIR), whereas coating with AuNPs layer will additionally support excellent photothermal therapy (PTT) due to nanoplasmonic effect [78]. Similarly, ZnO quantum dots (QDs) with pH-responsive and gatekeeping features can be integrated into plasmonic AuNP@mSiO_2_ nanocomposites, thus simultaneously enabling PTT and on-demand release of therapeutic doxorubicin (DOX) [79]. Intriguingly, this ZnO-triggered nanocomposite also shows distinct antitumor immunity through inducing immunogenic cell death [79], which implies the intrinsic immunomodulatory functions of inorganic nanomaterials should also be taken into account when designing multifunctional/multimodal inorganic nanoplatforms. Some metallic/inorganic NPs can directly mimic the catalytic functions of natural enzymes, so-called nanozymes, that are capable of catalyzing various in vivo chemical reactions in a single-substrate or multisubstrate manner [80]. These inorganic nanozymes accordingly respond to alterations of in vivo microenvironments’ components such as pH, H_2_O_2_, glutathione (GSH), and O_2_, favoring disease management through eliminating dangerous molecules or generating therapeutic agents using their enzyme-mimicking catalytic functions [80]. Gao et al. [81] synthesized a dendritic mSiO_2_ NPs coloaded with glucose oxidase-mimic AuNPs and peroxidase-mimic Fe_3_O_4_ NPs. This nanoplatform could accumulate in tumorigenesis site via EPR effect, and oxidize β-d-glucose into gluconic acid and H_2_O_2_ [81]. The resultant H_2_O_2_ is subsequently catalyzed by Fe_3_O_4_ NPs to high toxic hydroxyl radicals (·OH) which induces tumor-cell apoptosis [81]. However, potential toxicity of reactive oxygen species (ROS) generated by nanozyme in normal tissues is of particular concern. To overcome this issue, Hu and coworkers designed a biodegradation-mediated enzymatic activity-tunable molybdenum oxide nanourchins (MoO_3−x_ NUs) which catalyze production of high concentration of ·O_2_^−^ at acidic tumor microenvironment for inducing tumor-cell apoptosis [82]. While exposing to physiological environment, MoO_3−x_ NUs will catalyze OH^−^ into the nontoxic H_2_O rather than high toxic ROS, thus avoiding side effects [82]. In contrast, inorganic nanozymes that eliminate excessive ROS are more desirable at management of inflammatory diseases such as inflammatory bowel diseases (IBDs) [71]. To eliminate ROS more efficiently at IBDs, Liu and coworkers established an integrated cascade nanozyme by introducing a superoxide dismutase (SOD)-like Mn (III) porphyrin and a catalase (CAT)-like Pt NP into a nanoscale Zr-based metal–organic frameworks (MOF), PCN222 [83]. The resultant cascade nanozyme named Pt@PCN222-Mn could transform the catalytic process of ·O_2_^−^ to H_2_O/O_2_ from inherent two transport steps to a high-performance cascade catalysis in single compartment. Such a high efficiency in ROS scavenging enables favorable therapeutic outcomes of Pt@PCN222-Mn in ulcerative colitis and Crohn’s disease.

#### Polymeric NPs

Polymeric NPs are typical class of drug delivery systems that are long-tested and reliable, and have previously been summarized in state-of-the-art reviews [84–86]. Fabricated from natural and/or synthetic polymers, polymeric NPs share a series of pharmacologically acceptable advantages for drug encapsulation and delivery [84]. Chitosan, alginate, and hyaluronic acid (HA) are the most representative natural polymers; due to excellent biocompatibility/biodegradation and tunable properties, they are regarded as suitable carriers for various agents including antitumor drugs, antimicrobials, genes, proteins, etc*.* [84]. Specifically, chitosan is a positively charged polysaccharide owning favorable membrane penetration and mucoadhesion properties and thus is typically employed to aid gene transfection, intracellular delivery, and mucodelivery [87–89]. Contrary to chitosan, alginate and HA are anionic polymers presenting superior safety and have been extensively used in drug delivery and tissue engineering [90]. Synthetic polymers (e.g., poly(lacticcoglycolic acid), PLGA) not only diversify administration routes and pharmacokinetics of cargo drugs, but also act as structural templates to establish other nanoplatforms such as biomimetic NPs. By adopting aim-guided surface chemistry and/or integrating multicomponent polymers, multifunctional polymeric NPs can be obtained to achieve more adaptive spatiotemporal transport of cargo as well as improved biophysicochemical properties, which might be more desirable to overcome clinical difficulties. Accumulated ROS and hypoxia in pathogenetic tissues delay tissue regeneration thus worsening myocardial infarction. To resolve this issue, Ding and coworkers synthesized ROS-cleavable hyperbranched polymers that coassembled with methacrylate HA to form an injectable hydrogel under UV irradiation [91]. Incorporation of biocompatible catalase conferred this hydrogel with H_2_O_2_ degradative function for O_2_ generation, and the ROS-cleavable polymers degraded once contacting with excessive ROS, contributing to drug release and ROS scavenging [91]. The dual-action hydrogel platform showed advanced therapeutic efficacy against myocardial infarction, as indicated by removal of excessive ROS, inhibition of cell apoptosis, and improved angiogenesis, etc*.* [91]. Methacrylated gelatin (GelMA) could chemically cross-link with N-(2-aminoethyl)–4-(4-(hydroxymethyl)–2-methoxy-5-nitrosophenoxy) butanamide (NB)-modified HA (HA-NB) through UV-triggered click reaction between aldehyde groups and amino groups [92]. The resultant hydrogel fabricated by Hong and coworkers presented strong biomechanical properties and rapidly prevented pig heart bleeding, implying a significant potential to resolve the unmet challenges in surgical bleeding [92]. In treatment of heterotopic ossification, to avoid wide distribution of therapeutic rapamycin and achieve targeted delivery, Chen and coworkers employing collagen hybrid peptide (CHP) to decorate PLGA NPs; the CHP-decorated PLGA NPs could transport rapamycin specifically to pathological tendon collagen [93].

#### Liposomes

Liposomes are nanoscale lipid bilayer vesicles composed of phospholipids and cholesterol, which have advantages of high drug encapsulation, favorable biocompatibility and biodegradability, reduction of drug toxicity, slow-release behavior, passive targeting, ease of surface engineering, and optimizing pharmacokinetic properties, and have been widely investigated as drug carriers for delivery of small molecules, peptides, proteins, genes, and antibodies, with kinds of liposomal products approved such as liposomal DOX and liposomal amphotericin B [44, 94, 95]. It has a hydrophilic core and a hydrophobic layer, thus enabling favorable entrapment ability for both hydrophilic and lipophilic drugs [44]. Due to their broad-panel drug encapsulation, liposomes have already been extended to establish combinatorial therapeutics. Water-soluble DOX and lipophilic hispolon can be simultaneously loaded in the aqueous core and lipid shell of liposomes, respectively [96]. The obtained DOX/hispolon-codelivered liposomes show dramatical improvements in activity against melanoma cells [96]. Besides, liposomal formulation coloaded cytarabine and daunorubicin has also retrieved synergistic improvements in efficacy and pharmacokinetic properties, which has been approved by FDA for treatment of acute myeloid leukemia [97]. More importantly, liposome-based platforms are usually employed to transform toxic drugs to druggable nanomedicines with adaptive safety profiles and high therapeutic indexes. For example, cabazitaxel could overcome taxane-resistant cancer cells due to its low affinity to P-glycoprotein, however, only shows limited clinical applications because of severe systemic toxicity to patients. Shi and coworkers proposed a combinatorial strategy that integrated PUFAylation prodrug technique into liposomal scaffold, thereby resulting in a cabazitaxel prodrug-formulated liposome (lipoprodrug) which exhibited excellent in vivo tolerability, targeted accumulation in tumor tissues via EPR effect, and prolonged half-life [98]. Nevertheless, conventional liposomes usually require additional surface modifications to overcome potential disadvantages such as clearance by reticuloendothelial system (RES), drug leakage, poor stability, undesirable tissue distribution [44]. PEGylation is the representative strategy for surface engineering of liposomes to avoid RES barrier and favor stability; however, it will impede the normal release of therapeutic agents and cell uptake [44]. Alternative strategy proposed by Tang and coworkers might be able to resolve this paradox [99]. They designed a CD47-derived, enzyme-resistant peptide ligand named D-self-peptide to functionalize the surface of liposomes. The D-self-peptide could interact with signal regulatory protein alpha (SIRPα) in phagocytes, thus triggering an inhibitory signal counteracting phagocytosis. Such a “don’t-eat-me” signal protects liposomes from RES capture [99]. In addition to RES barrier, blood–brain barrier (BBB) also needs proper surface chemistry to facilitate central nervous system (CNS) delivery using liposomes or other nanoparticles. Aβ_25–35_, a truncated peptide derived from Aβ_1–42_ that assemble into the pathological plaques in Alzheimer’s disease, directly forms complexes with apolipoproteins to traverse the BBB [100]. Surface modification using Aβ_25–35_ has been proven to indeed promote the brain delivery of DOX-loaded liposomes through receptor-mediated transcytosis [100]. Furthermore, dual-functionalization (e.g., PEGylation combined with cRGDylation) integrating multiple superiorities into one liposomal formulation, such as long circulation, improved biodistribution, increased accumulation of therapeutic agents in target tissues, enables construction of multifunctional, multimodal, and spatiotemporal-responsive liposomes for advanced drug delivery [101].

#### Biomimetic NPs

Billions of years’ biological evolution has evolved a variety of multifunctional and sophisticated biological operative elements to adapt/change the complex living system paradigms regulated by the whole ecological environments as well as the continuous evolution and biogenesis of diseases [102]. For the perspective of disease management including diagnosis and treatment, biomimetic therapeutics that look toward natural environments and/or living systems for inspiration are fascinating and highly effective [102]. Similarly, introducing biomimetic ideology into nanomedicine naturally becomes a promising option in modern medicine because of integrating synergistic advantages, thus generating the field of biomimetic nanotechnology which has proverbially been used in vaccines design, rational drug delivery, tissue engineering, cancer theranostics, and inflammatory modulation, etc*.* [102–105]. Structurally programming synthetic nanoplatforms, inspired by diverse biological events such as ligand-receptor recognition and intracellular communication, to mimic or replace living functions, represents one of the most typical pipelines to construct biomimetic NPs capable of achieving effect amplification or competitive inhibition. To develop broad-spectrum antiviral treatments, many biomimetic NPs were designed to prevent the initial invasive step of virus to host cells through competitive blocking the interactions between viral attachment ligands (VALs) and its target receptors (*e.g.,* heparan sulfate proteoglycans, HSPGs) on host cell surface [106]. Whereas the most crucial problem for HSPG-mimicking NPs is how to expose sulfonate groups on surface of nanomaterial cores more efficiently, which would determine the consequent antiviral effect. Cagno and coworkers found replacing the short linker—3-mercaptoethylsulfonate (MES) responsible for surface display of sulfonate groups on AuNPs with longer linker—undecanesulfonic acid (MUS) significantly enabled and stabilized the multivalent binding between the surface sulfonate moieties for mimicking HSGPs and virus, thereby transforming the antiviral biomimetic NPs from virustatic to virucidal [106]. For vaccination, Wang and coworkers designed a pulmonary surfactant (PS)-biomimetic NPs loaded with cGAMP (an agonist of the stimulator of interferon genes), which can extend the protective spectrum of influenza vaccines from homologous to heterosubtypic viruses and prolong the maintenance of lung-resident memory CD8^+^ T cells for at least 6 months [107]. Such a biomimetic nanoplatform was only fabricated using conventional materials for liposome preparation including DPPC, DPPG, cholesterol, and PEG2000, but whereas it can mimic the lipid composition and charge of the lung PS and overcome the PS barrier to achieve the effective delivery of cGAMP into alveolar macrophages and subsequent alveolar epithelial cells for strengthening T cell immunity [107]. Further investigation demonstrated that uptake by alveolar macrophages of PS-biomimetic liposomes was dependent on surfactant protein A and D-mediated endocytosis [107]. The aforementioned studies inspire us that rationally manipulating structures and formulations of conventional nanomaterials might achieve de novo functions to overcome existing challenges because of being conferred biomimetic features. Nevertheless, using conventional nanomaterials is difficult to maximally mimic the cell morphology and functions for specific uses, especially for biodetoxification and inflammatory neutralization [104, 108]. Though attaching natural ligands or functional moieties to surface of synthetic NPs indeed do excellent work in replicating individual biological events found in nature, it is rather unachievable for such bottom-up strategy to replicate the collective natures of biological systems [104]. Thus, scientists directly extract cell-derived substances for in vitro engineering of naked NPs to disguise parent cells. Molinaro and coworkers extracted membrane proteins from leukocytes and integrated these proteins into synthetic phospholipid bilayer for preparing leukocyte-mimicking liposomes (leukosomes) [109]. The resultant leukosomes with highly homologous surface natures of leukocytes preferentially targeted inflamed vasculature with fivefold and eightfold enhancement of accumulation in inflamed tissues at post-inflammation 1 and 24 h, respectively, consequently contributing to effective delivery of dexamethasone for inflammatory attenuation [109]. Besides, surface layer proteins isolated from *Lactobacillus helveticus* can reassemble onto surface of positively charged liposomes [44, 45]. The resultant *Lactobacillus helveticus*-biomimetic liposomes partially preserved unique features of source bacterium, including increased rigidity and gastrointestinal adhesion [45]. Despite successfully reproducing natures of membrane proteins and preserving some beneficial properties, however, cell membrane comprises complex components including a mixture of lipids, proteins, and carbohydrates all of which jointly participate in the whole interactions with surrounding microenvironments [102, 104, 108], hence replicating systematic natures of cell membranes is more desirable, whereas it is difficult for the aforementioned biomimetic nanotechnology to meet this requirement. To this end, cell membrane coating nanotechnology has been developed [104], which directly isolates cell membranes to coat the surface of synthetic NPs such as polymeric NPs, inorganic NPs (e.g., silica NPs, AuNPs, and iron oxide NPs), nanogels, protein NPs, and MOF [104]. This biomimetic nanotechnology sufficiently preserves the physicochemical and physiological features of source cells. There are multiple cells involved in cell membrane coating technology, including red blood cell (RBC), platelet, immune cell, cancer cell, stem cell, and bacterial cell. The RBC membrane coating, a most common cell membrane coating strategy, endows synthetic NPs with RBC surface properties for avoiding RES capture, thus improving pharmacokinetics, and as nanosponges also enables adsorption of bacterial toxin (*e.g.,* β-hemolysin/cytolysin of group B *Streptococcus*) [110]. Another important membrane source, platelet, is naturally responsive to various biological processes such as coagulation and wound healing as well as participating in the pathogenesis of various diseases, hence can be coated on synthetic NPs to confer a variety of biointerfacing properties including immunocompatibility, pathogen binding, and adhesion to damaged vasculature, and tumor-targeting ability [111]. For example, platelet membrane-coated MOF could specifically deliver siRNA into the tumor cells through biointerfacing interactions, and the MOF responds to the pH reduction in endosomes leading to the release of siRNA for tumor gene therapy [112]. Such a biomimetic NPs perfectly combines both advantages of biomimetic coating and synthetic nanomaterials, and hence provides spatiotemporal-dependent delivery and on-demand release behaviors. Membrane coating derived from immune cells usually exhibits advantages of targeting inflammatory microenvironment, cytokines/PAMPs neutralization, blocking infectious events, and so on. Zhang and coworkers adopted CD4^+^ T cells as membrane donor to coat PLGA NPs, and obtained a nanoengineered CD4^+^ T cell membrane-coated NP (TNP) with broad-panel activity to 125 HIV-1-pseudotyped viruses [113]. As a therapeutic agent, TNP can neutralize cell-free HIV-1 and induce autophagy of HIV-1 gp120-expressing cells, thereby doubly hindering the HIV-1 reservoir [113]. Compared with naked NPs, NPs coated with cancer cell-derived membrane show lower distribution in normal tissues and improved accumulation in tumor tissues and hence have been widely used as carriers of imaging agents and/or therapeutic drugs for cancer theranostics [105]. Tapeinos and coworkers fabricated a biomimetic NPs composed of a Fe_3_O_4_/MnO_2_ inorganic core and a U-251 MG cell-derived membrane coating [114]. Such a nanoplatform display favorable homotypic targeting ability for glioblastoma multiforme-targeted drug delivery [114]. Meanwhile, the superparamagnetic inorganic core can be used as a diagnostic agent for magnetic resonance imaging. Of further note, there are numerous “markers of self” and “self-recognition molecules” expressed on cancer cell membranes [105]. By taking advantage of these characteristics, in vitro engineering wild-type cancer cells to express additional immune stimulatory molecule CD28 that coordinates with inherent antigen MHC-1 to co-stimulate T cell immunity [115]. AuNPs coated with membrane derived from this engineered cancer cells indeed promoted potent tumor antigen-specific immune responses [115]. With the development of biomimetic nanotechnology in modern medicine, other types of source cells can be rationally selected to decorate NPs, according to specificity of different diseases. Coating bacterial outer membrane derived from Helicobacter pylori onto surface of PLGA NPs resulted in bacterium-mimicking NPs (termed OM-NPs); the OM-NPs preserved adhesive capability toward gastric epithelial cells and thus compete with source bacteria for binding to the host cells [116]. This bacteriummimicking nanomedicine might be an alternative therapeutic for antibacterial applications because it may alleviate resistance development. Inspired by pathogenetic mechanisms of COVID-19, the target cells including human lung epithelial type II cells and macrophages for SARS-CoV-2 invading hosts might be desirable membrane donors for establishing specific biomimetic NPs to block SARS-CoV-2 infection [117]. Zhang and coworkers successfully fabricated two SARS-CoV-2-targeted nanosponges based on PLGA core and biomimetic membrane coating derived from human lung epithelial type II cells or macrophages [117]. Both of two nanosponges prevented SARS-CoV-2 infecting Vero E6 cells with IC50 value of 827.1 μg/mL and 882.7 μg/mL, respectively [117]. Nevertheless, in vivo efficacy and safety still need to further validation.

### Why are Nanoplatforms Desirable for Sepsis Management?

Before we evaluating the feasibility of nanoplatforms in managing sepsis, we always ask why are they suitable for sepsis management or what factors determine such a clinical possibility. For these critical problems, only sufficiently understanding the key points and unresolved challenges involved in clinical sepsis management and corresponding advantages of nanoplatforms, can we conclude the most reliable answer. Sepsis is an acute syndrome complicated with infection, host response dysregulation, and multiorgan failure [1, 15]. Consequently, one of the most key points is how to timely identify sepsis for early warning, which is very important to reduce the mortality [16]. However, diagnosis of sepsis requires detection of a series of biomarkers such as pathogens, C-reactive protein (CRP), procalcitonin (PCT), and cytokines, which usually are time-consuming using conventional methods (e.g., cell culture and enzyme-linked immunosorbent assay (ELISA)), delaying the golden time for rescuing sepsis patients. Nanomaterials-based diagnostic platforms (termed nanodiagnostic platforms) might be able to overcome these unresolved challenges. On the one hand, high surface area-to-volume ratios and versatile surface chemistry enable nanomaterials to display detection-associated molecules (e.g., antibodies) more effectively, which provide signal amplification and ultrasensitivity to target biomarkers; on the other hand, some metallic NPs present nanoplasmonic effect when exposed to light ranging from visible to near-infrared wavelength, that rapidly transforms the interactive events between biomarkers and plasmonic NPs to optical signal for readout [75, 118]. Nanodiagnostic platforms integrated these advantages are theoretically desirable for early warning and identification of sepsis. Furthermore, owing to their high sensitivity and rapid detection, nanodiagnostic platforms also provide considerable possibilities for early screening patients once admitted into ICUs in the future.

With regard to the perioperative therapy, large-dose usage of antibiotics is required to eliminate pathogens, which, however, significantly raises the risk of resistance development [10, 19, 35]. A variety of nanomaterials, especially these are rich in basic groups, show broad-spectrum bactericidal activity and have already been used in medical care such as advanced wound management [119]. They usually exhibit low frequency of resistance because they typically disrupt bacterial envelope rather than targeting specific molecular target [119]. Nevertheless, potential in vivo toxicity of these antimicrobial nanomaterials might be a concern that should be carefully evaluated in sepsis models before translating to clinical application. Moreover, transforming conventional antibiotics into nanobiotics by chemical modification has reported enhancing the antibacterial activity compared with parent antibiotics [120]. Through proper surface functionalization, sophisticated nano-based delivery systems enable targeted delivery of antibiotics into infectious microenvironment and subsequent on-demand release, which can ensure the therapeutic efficacy and simultaneously reduce the administrated dosage [120]. These existing superiorities imply a promising possibility for nanomaterials-based therapeutic platforms (termed nanotherapeutic platforms) to overcome the unresolved challenge in antimicrobial strategy involved in sepsis management. Up to now, therapeutics that effectively managing immune dysregulation have remained unavailable. Systemic administration of immunosuppressants (*e.g.,* antagonists of toll-like receptors (TLRs)) leads to severe side effect and might worsen outcomes when progress to “immunoparalysis” period [34, 121, 122]. In this case, biomimetic nanomedicines might be able to resolve this difficulty [102, 104]. Immune cell membrane-inspired biomimetic NPs capable of neutralizing bacterial toxins, cytokines, or targeting inflammatory microenvironment are of particular promises [104]. They directly eliminate excessively accumulated PAMPs and DAMPs rather than simply compromise immune systems, thereby might contribute to attenuated organ injury. Besides, biomimetic NPs with inflammation-targeted nature represent reasonable carriers for antibiotics to exert synergistic therapy. Accumulated ROS represents another class of dangerous molecules threatening organ functions, while nanozymes could eliminate ROS through catalyzing in vivo redox reaction [71, 123]. After treatment, monitoring prognosis is particularly important, which can prevent recurrent sepsis. Nanodiagnostic platforms enabling accurate, rapid, and even real-time detection of target biomarkers consequently provide possibilities to establish adaptive prognosis-monitoring platforms in the future.

Despite considerable feasibility of nanomedicines in sepsis management, one of the most crucial problems we need to focus on is what are the pharmacokinetic fates of these nanoarchitectures after in vivo injection upon septic conditions, which directly determines druggable properties and translational possibilities. So far, there is no literature that systematically report pharmacokinetic features of nanomaterials in sepsis models. It is acknowledged that in vivo biodistribution, metabolism, degradation, and final fate of NPs are jointly determined by their inherent characteristics that can be, respectively, recognized as physical identity, synthetic identity as well as biomolecule corona [124]. Physical identity represents the intrinsic physical natures of nanomaterials or NP cores, including size, shape, surface charge, physical composition, hydrophilicity, superparamagnetic property, plasmonic property, and fluorescence, etc*.* [124, 125]. NPs with different average size or surface charge exhibit different organ accumulation and clearance after in vivo injection. For example, kidney will rapidly eliminate NPs with a diameter smaller than 10 nm because this diameter range (< 10 nm) adapts to the capillaries and renal corpuscles thus can be easily filtered from circulation [126], whereas NPs with a diameter of > 50 nm are mainly accumulated in liver and spleen, controlling average size into range of 100–220 nm contributes to EPR effect under tumor conditions [127]. Besides, NPs with anionic surface tend to be taken up by liver, while the positively charged NPs preferentially contact with peripheral endothelial cells [124]. NPs with a diameter of <  ~ 100 nm, positively charged surface and low solubility usually are cytotoxic; the NPs with a diameter ranging from 100 to 220 nm, positively charged surface and low solubility , are easier to be captured by RES; while the NPs with the diameter of 100–220 nm, negatively charged surface and high solubility can show favorable EPR effect [127]. Inherent characteristics of some NPs such as superparamagnetic, plasmonic, and fluorescent are found to alter or track their in vivo biodistribution, degradation, and fate under exogenous stimuli, providing theranostic applications in disease management [128, 129]. Consequently, the final pharmacokinetics of different NPs are largely dependent on the balance of various physical identities. Of course, synthetic identity conferred by surface chemistry enables new pharmacokinetic behaviors, such as active targeting, specific cell affinity, and long circulation [130]. Combination of physical and synthetic identities cooperatively achieves differential pharmacokinetics of various NPs. Akiva and coworkers fabricated three RBC-coated/uncoated PLGA NPs with different shapes and observed in vivo biodistribution and half-life [131]. Compared to spherical NPs, prolate ellipsoidal and oblate ellipsoidal NPs avoid in vitro macrophage uptake more effectively, and such effect can be significantly enhanced after RBC coating [131]. After in vivo administration, RBC-coated NPs exhibit increased accumulation in spleen compared with uncoated NPs; lower amount of prolate ellipsoidal NPs accumulated in liver in comparison with other NPs, implying a decreased elimination [131]. As expected, RBC-coated prolate ellipsoidal NPs showed the dramatic increase in long circulation with a half-life of ~ 3 h and thus augmented in vivo efficacy in treating α-toxin-induced sepsis [131]. As for the biodegradation and in vivo fate of NPs in septic conditions, they may depend on the types and characteristics of NPs and different stages of sepsis progression, in our perspectives. Once entering blood circulation, NPs immediately expose to a highly complex physiological environment in which biomolecules such as proteins, lipids, and metabolites subsequently adsorb onto surface of NPs through a series of nonbond interactions, resulting in formation of biomolecule corona [124]. The biomolecule corona is not only determined by physiological conditions but also regulated by the inherent characteristics of NPs including physical and synthetic identities. Usually, biomolecule corona might largely change the expected pharmacokinetic properties of our designed nanoplatforms, causing significant difference between in vitro and in vivo efficacy. Nevertheless, proper programming nanoplatforms with full considering pathophysiological features of diseases could transform the unwanted biomolecule corona to sophisticated surface engineering strategy for targeted delivery and expected pharmacokinetics [100]. Sepsis involves severe infections, systemic inflammatory abnormalities, and organ dysfunction, whose in vivo pathophysiological environment is different from any other diseases that nanomedicines are widely applied (e.g., cancer). Hence, the pharmacokinetic processes of NPs in septic conditions are different from that in other diseases or healthy conditions, but we still can get some clues from these widely investigated conditions. In the early stage of sepsis, severe infections trigger highly proinflammatory cascades which result in considerable accumulation of cytokines and infiltration of immune cells, consequent creating infectious/inflammatory microenvironment (IME) that possesses similar characteristics of tumor microenvironment (TME) (e.g., rich bloodstreams, infiltration of immune cells, and enhanced permeability of vessels). In this case, NPs with fine-tunable size might passively target IME via EPR-like effect. Of course, due to the hyper immune responses in this stage, NPs have large possibilities to be rapidly captured and subsequently eliminated by RES, thus require additional surface engineering (e.g., PEGlyation and RBC coating) to avoid such a fate. Some biocompatible NPs might be catalyzed by the abundant enzymes in IME and degraded finally. Other types of NPs (e.g., inorganic/metallic NPs) in our opinion would be metabolized and degraded or excreted by liver and/or kidney. When progressing to late stage, multiple organs after experiencing severe infections and proinflammatory storms will suffer serious tissue damage and dysfunction. Hence large-dose administration of synthetic NPs might cause lethal side effects. In this case, using biomimetic NPs might be more reasonable. Furthermore, alterations in hemodynamics (e.g., coagulation abnormality) might also impair the pharmacokinetic properties of NPs. Immunosuppression and bacterial regrowth co-occur in this stage, which create a unique IME without inflammatory properties. How NPs interact with this IME represents a meaningful topic to be in-depth investigated in the future, which may advance development of precision nanoplatforms for sepsis management.

## Nanodiagnostic Platforms for the Accurate and Rapid Detection of Sepsis-Related Biomarkers

Point-of-care management requires early warning and diagnosis [34]. However, the complexity of pathogenesis for aspects including infection, immune abnormality, and organ dysfunction can prevent rapid identification and subsequent therapy [10]. Clinically, the SOFA scoring system has been adopted to evaluate the severity of organ injury [1]. Infection and inflammation generally depend on the bacterial culture and characterization of biochemical indicators. Given the versatile pathophysiology, multifaceted biomarkers have been proposed to collaboratively identify sepsis [132]. Living bacteria detection helps to determine the species of pathogens, thereby guiding antibiotic usage. C-reactive protein (CRP) and procalcitonin (PCT) are relevant to the susceptibility to systemic infection. Plasma endotoxins and cytokines (e.g., IL-3, IL-6 and TNF-α) are employed to reveal inflammatory progression. However, current detection strategies for these biomarkers usually require intricate procedures and expensive costs, which dramatically delay effective treatments and patient compliance. Intriguingly, efforts that combine nanotechnology with sepsis diagnosis have made promising progress. By taking advantage of nanoscale platforms, some limitations or issues impeding traditional strategies for clinical use can be solved effectively. We summarize some interesting nanodiagnostic platforms for the identification and quantification of sepsis-related biomarkers in Table 1 [133–165]. Several nanodiagnostic platforms to detect sepsis-associated biomarkers, including bacteria, CRP, PCT, and cytokines, are discussed in this section.

**Table 1:** Representative nanodiagnostic platforms for the detection of sepsis-relevant biomarkers

Classification of biomarkers	Nanodiagnostic platforms	Supramolecular nanoarchitectures	Detected mechanisms	LOD/response time	References
*Bacteria*					
*E. coli*	Bacteria-instructed click chemistry-associated colorimetric systems	Azide/alkyne-functionalized AuNPs	Biomimetic strategy based on bacterial metabolic pathway: Cu^+^-catalyzed click chemistry (alkyne-azide cycloaddition)	40 CFU mL^−1^ Within 1 h	[133]
Polymyxin B-resistant *E.coli*	Chiral upconversion heterodimers	Upconversion NPs; gold yolk-shell NPs	Biomimetic strategy based on antibiotic–bacterial interaction: different interactions of polymyxin B with lipid A from wild-type strain and resistant strain	8 h	[134]
Polymicrobes	Microvesicle-based microfluidic chip	PMNs-derived microvesicles	Biomimetic strategy based on cell-bacteria interactions: microvesicle-bacteria aggregation	Within 1.5 h	[135]
*E. coli* *S. typhimurium* *S. aureus*	Multicolored FRET silica NPs	Silica NPs	Immune (antibody detection)	Within 30 min	[136]
Urease-positive bacteria (*Proteus mirabilis*)	Plasmonic nanosensors	PDDA-coated magnetic beads; gold NPs	NH_3_-dependent assembly of gold NPs	10^1^ cells mL^−1^ 40 min	[137]
*P. aeruginosa* *S. aureus*	raGNPs-integrated NS-MFS	raGNPs	Immune (antibody detection)	10 CFU mL^−1^ 30 min	[138]
*E. coli*	Procedure combining biofunctional magnetic NPs and fluorescent probes	Vancomycin-functionalized FePt NPs	Biomimetic strategy based on antibiotic–bacteria interaction: multivalent interactions mediated by vancomycin	10 CFU mL^−1^ 2 h	[139]
*CRP*					
CRP	Nanoparticle-enhanced plasmonic biosensor	Gold NP-integrated gold nanohole arrays	Immune (antibody detection)	27 pg mL^−1^ 2 h	[140]
CRP	Magnetic NP-based biosensor	Magnetic NPs	Immune (antibody detection)	0.6 pg mL^−1^	[141]
CRP	Carbon nanofiber-based biosensor	Carbon nanofiber	Immune (antibody detection)	~ 11 ng mL^−1^	[142]
CRP	Copper oxide/zinc oxide composite Nanosurface-based immunosensors	ZnO–CuO nanocrystal surface	Immune (antibody detection)	–	[143]
CRP	Iron oxide nanoparticle-linked immunosorbent assay (ILISA)	Iron oxide nanocrystal	Immune (antibody detection)	Subpicomolar	[144]
CRP	Zinc oxide-based nanosurfaces	Zinc oxide nanocrystal	Immune (antibody detection)	< 1 ng mL^−1^	[145]
CRP	Fluorescent fullerene NP-based lateral flow immunochromatographic assay	Fullerene nanoparticles	Immune (antibody detection)	0.1 ng mL^−1^ 15 min	[146]
CRP	Gold nanorod-based dielectric voltammetry detection	Gold nanorod	Immune (antibody detection)	10 fM	[147]
CRP	Vertical flow-based point-of-care immunokit (VFIK)	Gold nanoconjugates (GNC)	Immune (antibody detection)	10 ng mL^−1^ 1–2 min	[148]
*PCT*					
PCT	Amperometric immunosensor	Ferrocene-modified gold NPs	Immune; electrochemistry	0.8 pg mL^−1^	[149]
PCT	Ultrasensitive electrochemical immunosensors	PtNP-Fc-C_60_ nanocomposites	Immune; electrochemistry	6 pg mL^−1^	[150]
PCT	Nanocomposite signal tag-based electrochemical immunosensors	Cu/Mn double-doped CeO_2_ nanocomposites	Immune; electrochemistry	0.03 pg mL^−1^	[151]
PCT	Fiber optic nanogold-linked immunosorbent assay (FONLISA)	mSAMs-coated AuNPs	Immune sensing (antibody detection)	7.3 fM 15 min	[152]
PCT	Ultrasensitive sandwich electrochemical strategy	Reduced graphene oxide (rGO)–gold (Au) nanocomposite film; single-walled carbon nanohorns (SWCNHs)/hollow Pt chains (HPtCs) complex	Immune sensing (antibody detection)	0.43 pg mL^−1^	[153]
PCT	Time-resolved digital immunoassay	Streptavidin-coated AuNPs	Immune sensing (antibody detection)	~ 2.8 pg mL^−1^ ~ 25 min	[154]
PCT	Porous layer open tubular-signal amplification (PLOT-SA) sensor	Streptavidin–biotin-HRP nanocomplex	Immune sensing (antibody detection)	0.01 pg mL^−1^	[155]
PCT	Washing-free centrifugal microchip fluorescence immunoassay	Fluorescent microspheres	Immune sensing (antibody detection)	0.05 ng mL^−1^ 10 min	[156]
*Cytokines*					
IL-6	CuInS_2_/ZnS nanocrystal-based fluoroimmunoassay	CuInS_2_/ZnS nanocrystals	Immune; optical	0.008 ng mL^−1^	[157]
	Graphene-based fully integrated portable nanosensing system	Aptameric graphene-based sensing platform	Aptamer detection	12 pM 400 s	[158]
IL-6 TNF-α	Multiplexed cytokine detection nanoplasmonic platform with direct-write protein patterns	Nanoplasmonic AuNPs	Immune detection	–	[159]
IL-1β TNF-α	Electrochemical immunosensors	Carbon nanotubes	Immune; electrochemistry	0.38 pg mL^−1^ 0.85 pg mL^−1^	[160]
TNF-α	LSPR nanoplasmonic optofluidic platform	Nanoplasmonic AuNPs	Immune detection	4–5 h	[161]
IL-1β	Biotunable nanoplasmonic filter on few-layer MoS_2_	Nanoplasmonic AuNPs	Immune sensing; nanoplasmonic signaling	14 fM 10 min	[162]
IL-3	Integrated biosensor	Immunomagnetic beads	Immune; electrochemistry	< 10 ng mL^−1^ 1 h	[163]
IL-2 IL-4 IL-6 IL-10 TNF-α IFN-γ	LSPR-based microfluidic optical biosensing	AuNRs	Immune sensing; nanoplasmonic signaling	IL-2: 20.56 pg mL^−1^ IL-4: 4.60 pg mL^−1^ IL-6: 11.29 pg mL^−1^ IL-10: 10.97 pg mL TNF-α: 11.43 pg mL^−1^ IFN-γ: 6.46 pg mL^−1^ 40 min	[164]
IL-6 IL-8 MCP-1	Nano-in-micro-smart hydrogel composite	Immunosensing polystyrene nanobeads; stimuli-responsive microgel matrix	Immune sensing; fluorescence signaling		[165]

LOD, limit of detection; NPs, nanoparticles; AuNPs, gold nanoparticles; PMNs, polymorphonuclear cells; CFU, colony-forming units; FRET, fluorescence resonance energy transfer; PDDA, poly(diallyldimethylammonium chloride); raGNPs, redox-active gold nanoparticles; NS-MFS, nanosieving microfluidic system; *E. coli*, *Escherichia coli*; *S. typhimurium*, *Salmonella typhimurium*; *S. aureus*, *Staphylococcus aureus*; *P. aeruginosa*, *Pseudomonas aeruginosa*; CRP, C-reactive protein; PCT, procalcitonin; IL-6, interleukin-6; IL-1β, interleukin-1β; TNF-α, factor necrosis tumor α; IL-3, interleukin-3; LPS, lipopolysaccharide; sPLA2-IIA, secretory phospholipase 2-IIA; SAMs, self-assembled monolayers; LSPR, localized surface plasmon resonance; AuNRs, gold nanorods

### Nanodiagnostic Platforms that Detect Bacteria for the Identification of Sepsis

**Fig. 3:**
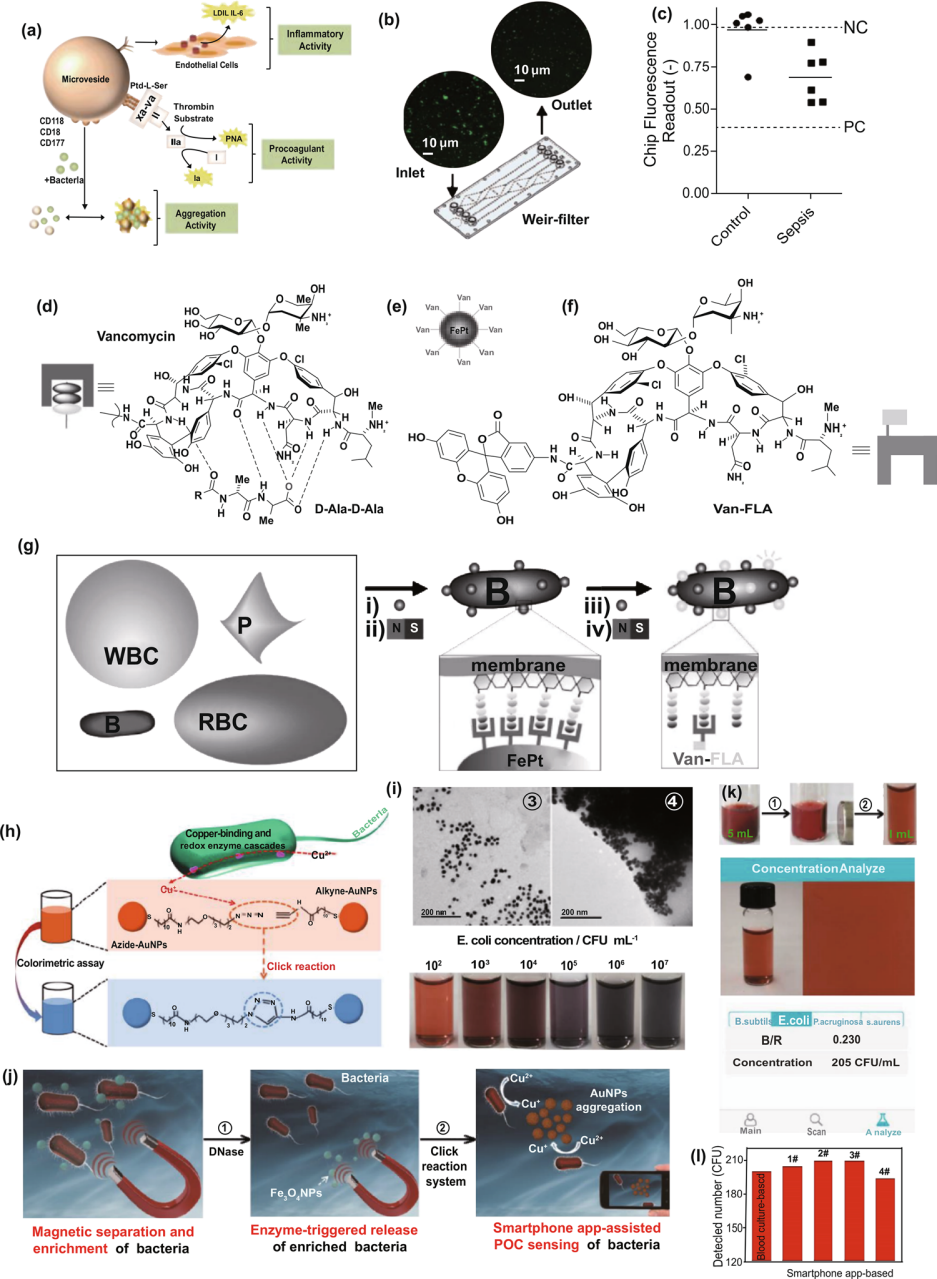
Innate immune cell-derived microvesicles differentiate sepsis from noninfectious systemic inflammation. **a** PMN-derived microvesicles induced bacterial aggregation, procoagulant activity and inflammatory response in endothelial cells. **b** PMN-derived microvesicle-based point-of-care-compatible microfluidic chip with four separation channels. **c** Detection of 12 clinical samples from control (*n* = 6) and sepsis (*n* = 6) patients using a microvesicle-based microfluidic chip. NC, negative control; PC, positive control. Reproduced with permission from Ref. [135]. Copyright 2015 Royal Society of Chemistry. Design and mechanism of the FePt@Van nanoparticle-Van fluorescent probe detection system for the rapid detection of bacteria in human blood. **d** Multivalent binding of Van to bacterial surface terminal peptide D-Ala-D-Ala. **e** Schematic drawing of FePt@Van nanoparticles. **f** Chemical structure of Van fluorescent probe (Van-FLA). **g** Illustration of bacterial detection step. Bacteria were captured by FePt@Van nanoparticles with magnetic assistance, and the resultant bacteria were stained by Van-FLA and magnetically separated from the blood separation. Reproduced with permission from Ref. [139]. Copyright 2006 John Wiley and Sons, Inc. Bacteria-instructed click chemistry-guided functionalized AuNPs for point-of-care microbial detection. **h** Conceptual mechanism of the colorimetric transformation of functional AuNPs triggered by bacteria-instructed click chemistry. **i** TEM images and colorimetric photographs indicating the bacteria-instructed click reaction between the azide- and alkyne-AuNPs, resulting in AuNP aggregation (TEM images) and color transformation (colorimetric photographs). **j** Schematic illustration of bacteria-instructed click chemistry-guided AuNPs sensor combining magnetic separation and smartphone app-assisted colorimetric strategy. **k** Experimental photographs for the click chemistry-guided AuNP sensing of *E. coli* from complex artificial sepsis blood samples. The concentration of *E. coli* could be analyzed by smartphone colorimetric strategy. **l** Detected *E. coli* numbers of four parallel artificial sepsis blood samples by the click chemistry-guided AuNP platform. Reproduced with permission from Ref. [133]. Copyright 2019 American Chemical Society

Individual antimicrobial treatment is advocated to prevent the spread of drug resistance during sepsis management. Hence, rapid recognition of infection severity and pathogen species is urgently needed to facilitate clinical treatments. The current gold standard to identify pathogens is cell culture. However, cell culture is usually laborious and time-consuming (e.g., several days). In addition, differentiating sepsis from noninfectious systemic inflammatory syndrome remains a challenge for clinicians. To overcome these issues, Herrmann et al. [135] focused on magical trigger-dependent microvesicles consisting of nanoscale membrane-bound fragments derived from innate immune cells (e.g., leukocytes and neutrophils) that are specifically responsive to bacteria. They dissected the septic sensibility of three natures of polymorphonuclear cell (PMN)-derived microvesicles, including inflammatory, procoagulant and aggregation activity (Fig. 3a). The results indicated that aggregation with bacteria represented the most sensitive nature for PMN-derived microvesicles to sense infections, suggesting that microvesicle-bacterial aggregation might be regarded as a strong candidate to differentiate sepsis from noninfectious inflammatory syndrome. To this end, the authors designed a point-of-care-compatible microfluidic chip based on PMN-derived microvesicles (Fig. 3b). The fluorescence intensity of the samples was recorded at the inlet and outlet of a microfluidic chip whose fluorescence readout was set to outlet/inlet. Twelve clinical samples (from 6 noninfectious patients and 6 septic patients) were detected using a microvesicle biosensor or clinical diagnosis. Under the clinical diagnosis strategy, 10 of 12 samples were diagnosed as the correct group, but the other 2 samples suffered false assignment. In contrast, only 1 noninfectious inflammatory sample was incorrectly assigned after screening by the microvesicle biosensor (Fig. 3c). Notably, the microvesicle biosensor contributed a more rapid response time (≤ 1.5 h) to accurate diagnosis. This strategy is based on the cell biomimetic principle, which inspires us to mimic or manipulate cell functions or natures and can achieve unexpected advantages in the development of nanodiagnostic platforms. For example, immune cell (*e.g.,* macrophage, neutrophil, and T cell)-derived exosomes might preserve the capacity to recognize bacteria and PAMPs. By exhibiting reasonable surface engineering and signal transduction, immune cell-derived exosomes can be implemented in biosensors for detecting bacteria and PAMPs, which will warn of infectious and inflammatory risks during sepsis progression in a timely manner.

In addition to immune cell-bacteria interactions, physicochemical interactions between antibiotics and bacteria represent another biomimetic strategy to develop nanodiagnostic platforms for the identification of sepsis. Gao et al. [139] reported a vancomycin-functionalized magnetic nanoparticle biosensor combined with fluorescence probes. Vancomycin capable of recognizing bacterial surface terminal peptide D-Ala-D-Ala through hydrogen bonds (Fig. 3d) [139, 166], served as a bacterial sensor to functionalize FePt magnetic nanoparticles (FePt@Van) (Fig. 3e). To introduce optical signaling, a unique fluorescence probe (Van-FLA) was generated by the conjugation of vancomycin with a fluorescent amine (Fig. 3f). The procedures involving this biosensor are shown in Fig. 3g. Significantly, the FePt@Van-Van-FLA detection system, which provided a rapid response time (≤ 2 h) with a low limit of detection (LOD) of 10 CFU/mL, was suitable for the detection of both gram-negative *E. coli* and gram-positive *Staphylococcus*. Nevertheless, the sensitivity of this nanodiagnostic platform to gram-negative bacteria might be weaker than that to gram-positive bacteria, owing to the narrow antimicrobial spectrum of vancomycin (sensitive only to gram-positive bacteria). Based on a similar principle, magnetic nanoparticles can be functionalized by other antibiotics, such as polymyxin B, that are capable of binding to LPS on the outermost membrane of Gram-negative bacteria, contributing to the specific identification of gram-negative sepsis. In addition, human defensins, a type of endogenous antimicrobial peptides, can specifically recognize the bacterial cytoskeleton to support broad-spectrum killing. In terms of this nature, we think human defensins are ideal biomaterials to construct nanorobots for bacterial separation and detection.

Versatile metabolic pathways can help bacteria metabolize various toxins that threaten bacterial survival. Manipulating versatile metabolic pathways and enzyme systems of bacteria has been considered as another biomimetic strategy to provide important rationales for the design of bacterial sepsis-detected nanodiagnostic platforms. By taking advantage of this biomimetic strategy, living bacteria in samples can be quantified effectively by observing the macroscopic presentations of metabolic pathway/enzyme-mediated chemical reactions. Based on these ideas, Mou et al. [133] innovatively introduced bacteria-instructed click chemistry into a gold nanodiagnostic platform for point-of-care microbial sensing in sepsis samples. The redox enzyme system generated by microbes exposed to the toxin Cu^2+^ will transform exogenous Cu^2+^ to Cu^+^ which subsequently catalyzes a click chemical reaction between azide-modified and alkyne-modified gold nanoparticles (AuNPs), producing AuNP aggregations with a color transformation from red to blue that supports a colorimetric strategy (Fig. 3h, i). Based on such rationale, adding azide/alkyne-AuNPs into enriched bacterial suspension will transform the bacterial number signaling to color signaling that can be further recorded by a portable smart phone to present a colorimetric quantification (Fig. 3j). Practically, this novel nanodiagnostic platform accurately identified and counted the amount of *E. coli* in artificial sepsis blood samples that contain multiple pathogens (Fig. 3k). Furthermore, multiple parallel trials proved that measurements by the nanodiagnostic platform exhibited a favorable match with those by blood culture (Fig. 3l), notably showing a low response time (< 1 h) and a high sensitivity of 40 CFU mL^−1^. In addition, bacterial lipase, an enzyme abundantly expressed in the infectious microenvironment, has previously been employed to develop stimuli-responsive nanomedicines and can also be introduced into the construction of nanodiagnostic platforms to detect bacteria. We can also utilize the pH difference between different amounts of bacteria to construct signal transduction for transforming the concentration signaling of hydrogen ions to bacterial count signaling. Nevertheless, the feasibility of these approaches remains to be proven.

**Fig. 4:**
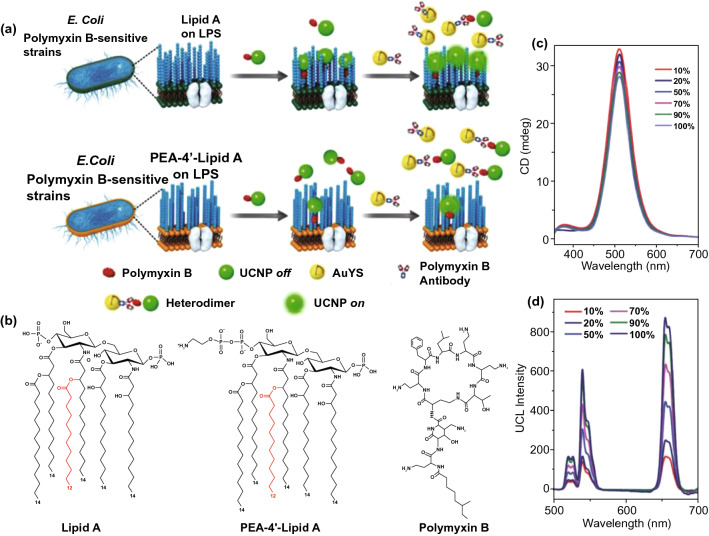
Chiral upconversion Au YS-UCNP heterodimers for the quantitative analysis and bioimaging of polymyxin B-resistant bacteria in vivo. **a** Schematic mechanism of Au YS-UCNP heterodimers for detecting polymyxin B-resistant bacteria. **b** Chemical structure of lipid A (from polymyxin B-sensitive strains), PEA-4′-lipid A (from polymyxin B-resistant strains) and polymyxin B. **c, d** The CD signal (**c**) and UCL intensity (**d**) of the Au YS-UCNP heterodimer after incubation with different ratios of polymyxin B-resistant bacteria and polymyxin B-sensitive strains. Reproduced with permission from Ref. [134]. Copyright 2018 John Wiley and Sons, Inc

Owing to constant antibiotic treatments, the emergence of drug resistance increases the life-threatening risks for patients who suffer sepsis. The timely diagnosis and analysis of antibiotic-resistant bacteria have significant clinical value. By adopting the biomimetic strategy for antibiotic–bacteria interactions, Sun et al. [134] developed a chiral upconversion heterodimer platform for the quantitative analysis and bioimaging of polymyxin B-resistant bacteria in vivo (Fig. 4a). Polymyxin B-resistant bacteria usually evolve a mutant LPS with *N*-acylethanolamine modification of lipid A, thus diminishing affinity between polymyxin B and resistant strains (Fig. 4b). By taking advantage of this differential affinity, a well-designed nanodiagnostic platform that comprises polymyxin B-immobilized upconversion nanoparticles (UCNPs) and polymyxin B antibody-functionalized gold yolk-shell nanoparticles (Au YS), was established. The UCNPs exhibit strong upconversion luminescence (ULC) with no circular dichroism (CD) signal in the absence of Au YS. Once UCNPs are conjugated with Au YS, a strong CD signal will be triggered, whereas the ULC will be shut down. Based on this signaling mechanism, addition of UCNPs and subsequent Au YS could present significant differences in ULC and CD signals between sensitive and resistant strains, thereby contributing to the successful identification of polymyxin B-resistant pathogens. As expected, with an increasing ratio of sensitive strains, the CD signals decreased, while the ULC intensities were enhanced significantly (Fig. 4c, d).

### Nanodiagnostic Platforms that Detect CRP for the Identification of Sepsis

**Fig. 5:**
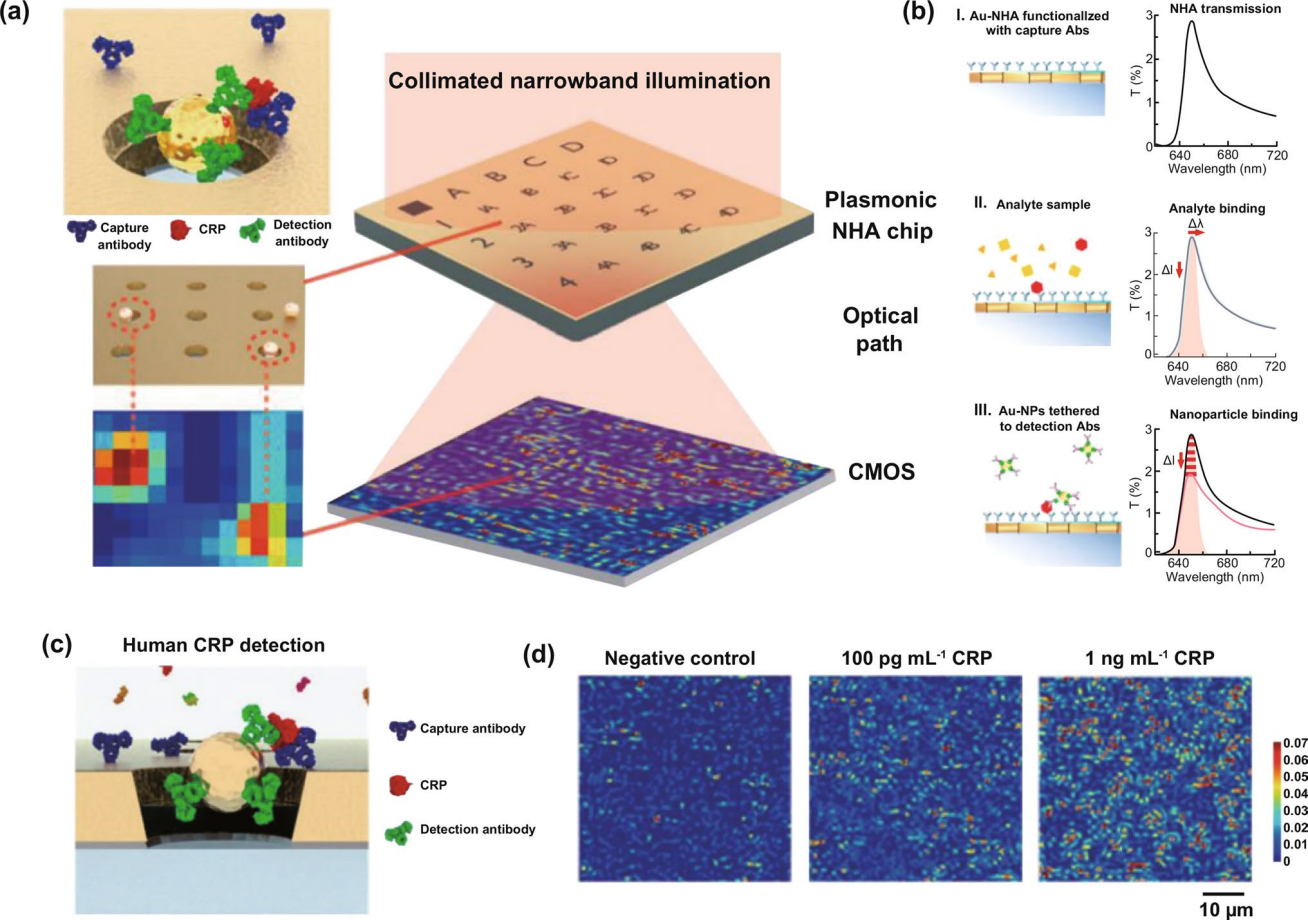
Nanoparticle-enhanced plasmonic biosensor for the rapid and precise detection of CRP. **a** Design and detected mechanisms of the nanoparticle-enhanced plasmonic imager. **b** The EOT peak variance of Au-NHAs in different steps during detection. **c** Human CRP sandwich assay. **d** Different concentrations of CRP can be visually distinguished on plasmonic imaging. Reproduced with permission from Ref. [140]. Copyright 2018 American Chemical Society

CRP, an acute-phase reactant, is positively correlated with infection and now serves as a classical biomarker to assist the diagnosis of inflammation-relevant diseases such as sepsis [167]. In particular, the clinical detection of CRP can be predominately employed to guide antibiotic treatment for sepsis, thus avoiding the disproportionate and excessive usage of antimicrobials [168]. However, the conventional laboratory strategies, enzyme-linked immunosorbent assay (ELISA) and fluorescent labeling, require complex executive procedures and expensive expenditure that are unsuitable for early warning and identification. In an attempt to improve the speed and accuracy, Belushkin et al. [140] created a nanoparticle-enhanced plasmonic biosensor for the rapid and precise detection of CRP. The nanodiagnostic biosensor consists of specific antibody-functionalized gold nanoparticles (AuNPs) and a large-area gold nanohole array (Au-NHAs) (Fig. 5a). The AuNPs can present a sharp extraordinary optical transmission (EOT) resonance with a dip and a peak in the far-field spectrum, which can be imaged by a complementary metal–oxide–semiconductor (CMOS) to characterize the signal intensity (Fig. 5a, b). Once target molecule was recognized by Ab-functionalized AuNPs, it created strong local transmission suppression in the far field, thus strengthening the bright-field imaging intensity. Such a bright-field imaging nanoplasmonic device indeed exhibits a distinct correlation between bright-field imaging intensity and CRP concentrations (Fig. 5c, d) and also contributes to a swift speed (< 2 h) and comparable sensibility (LOD = 27 pg/mL) with laboratory methods. In addition to the above-mentioned mental nanodiagnostic platforms, some other nanoplatforms that were previously used in drug delivery are also capable of supporting the rapid and precise detection of CRP for sepsis identification. Gupta et al. [100] designed a carbon nanofiber-based biosensor platform whose LOD for CRP detection was found to be approximately 11 ng mL^−1^. Magnetic nanoparticles were also considered to construct a biosensor that was used to sense CRP for the characterization of sepsis and necrotizing enterocolitis with an LOD of 0.6 pg mL^−1^ [141].

**Fig. 6:**
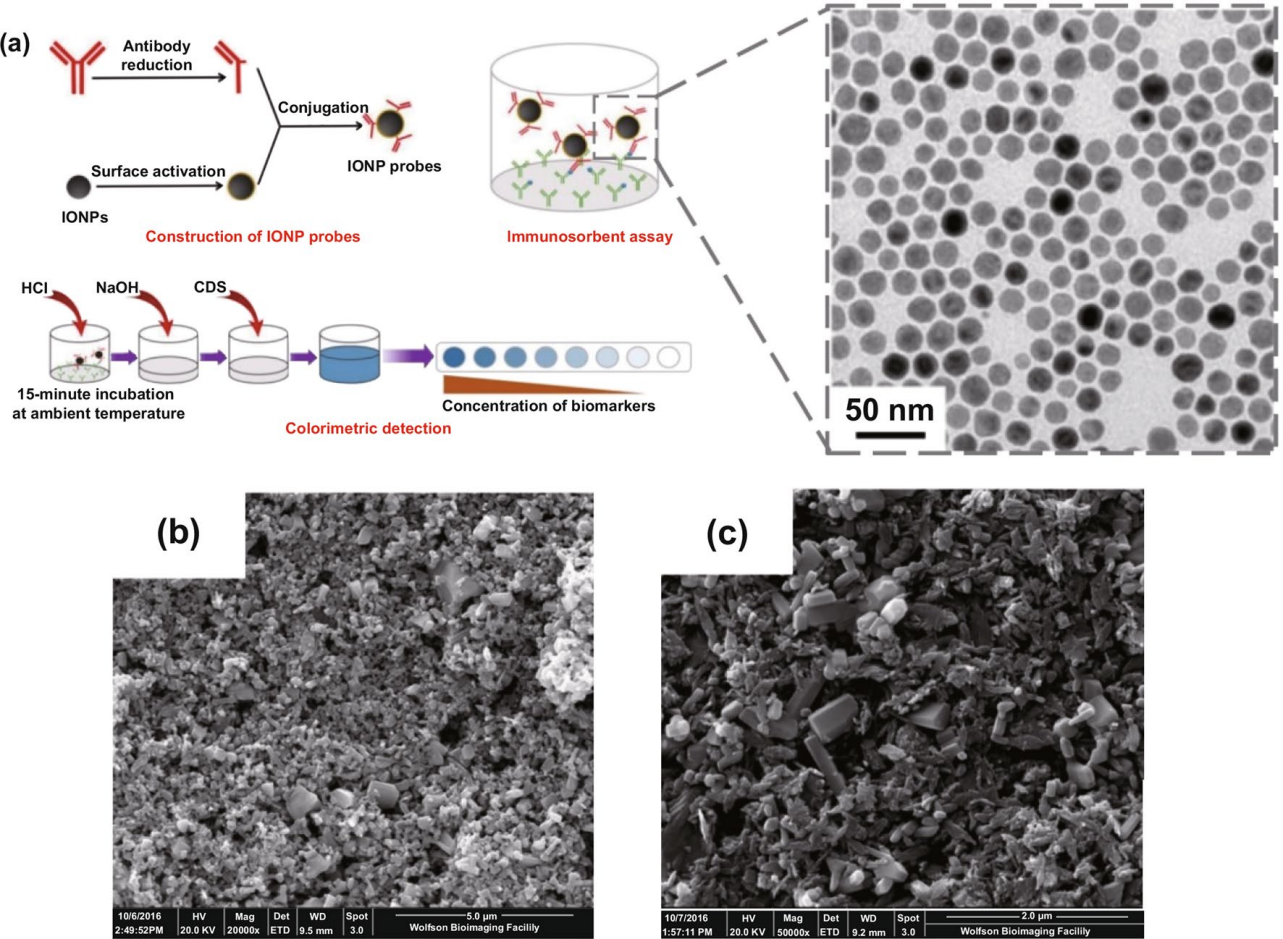
Nanocrystal-based diagnostic platforms for the detection of CRP. **a** IONP nanocrystal-linked immunosorbent assay (ILISA): schematic illustration of ILISA protocols for CRP quantification (left) and representative TME image of IONP nanocrystals. Reproduced with permission from Ref. [144]. Copyright 2016 Ivyspring International Publisher. **b** Representative SEM image of ZnO nanocrystal sensor surfaces on polyethylene terephthalate. Reproduced with permission from Ref. [145]. Copyright 2018 Nature Publishing Group. **c** Representative SEM image of the ZnO–CuO hybrid nanocrystal sensor surface. Reproduced with permission from Ref. [143]. Copyright 2019 MDPI

**Fig. 7:**
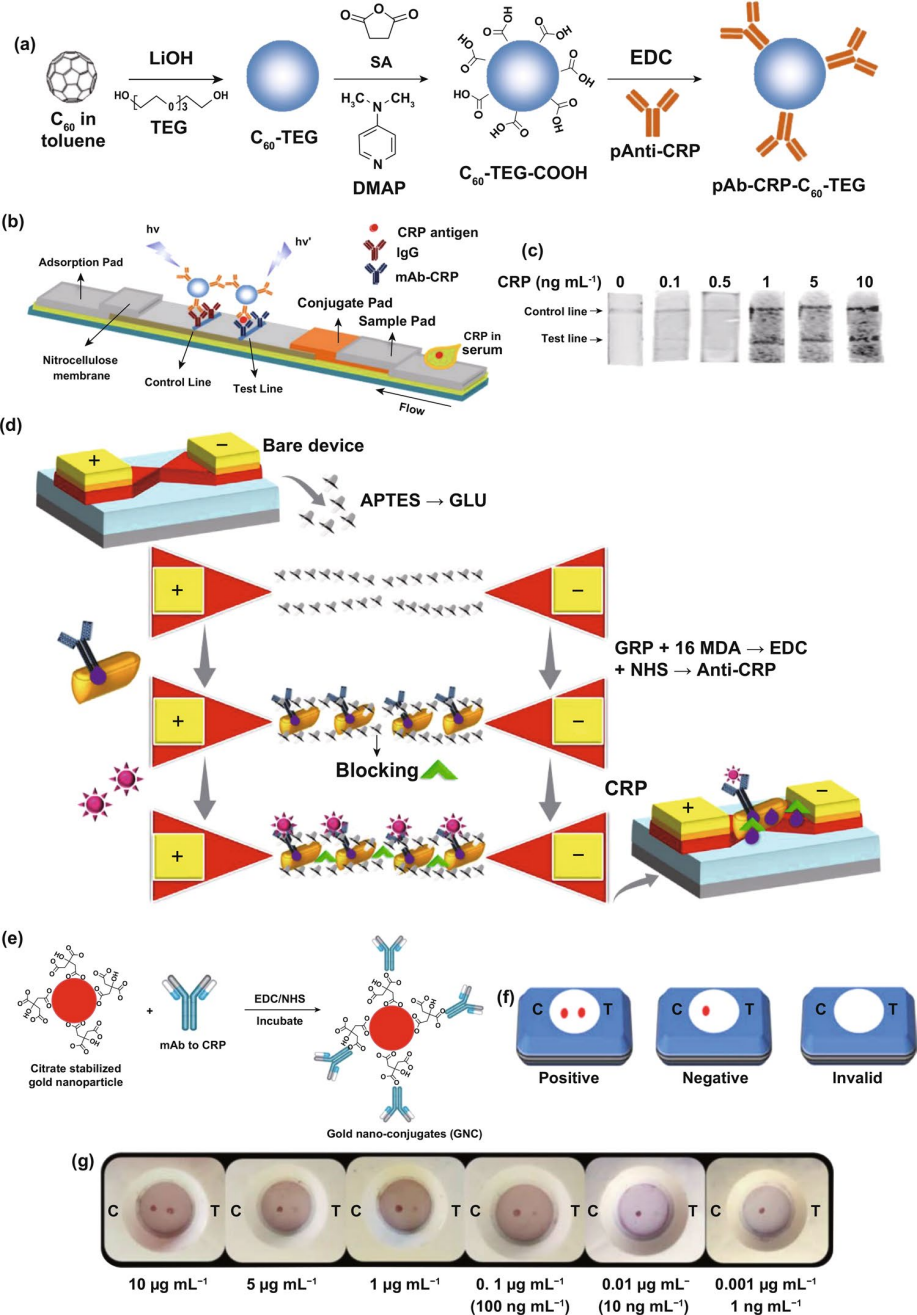
Fluorescent C_60_ nanoparticle-based lateral flow immunochromatographic platform for CRP detection. **a** Schematic illustration of the fabrication of pAb-CRP-C60-TEG fluorescent nanoprobes. **b** Schematic drawing and operative procedures of the pAb-CRP-C60-TEG fluorescent nanoprobe-based lateral flow immunochromatographic platform to detect CRP. **c** The fluorescence images of the test strips with various concentration of CRP from 0.01 to 10 ng/mL. Reproduced with permission from Ref. [146]. Copyright 2019 Springer Nature. **d** Schematic diagram for GNR-integrated voltammetry detection of CRP. Reproduced with permission Ref. [147]. Copyright 2019 Elsevier. GNC probe-based ultrasensitive vertical flow immunokit (VFIK) for the rapid detection of CRP. **e** Rational design and synthesis of GNC probes. **f** Schematic illustration indicating the prospective results presented by GNC-VFIK. **g** Test performance of GNC-VFIK with different concentrations of CRP in analytes. Reproduced with permission from Ref. [148]. Copyright 2020 Springer Nature

Owing to the large surface area, potent physicochemical stability, and fine-tunable surface engineering, nanocrystal-based architectures provide another route to develop nanodiagnostic platforms for biomolecular detection [169–171]. Zhang et al. [144] designed an iron oxide nanoparticle-linked immunosorbent assay (ILISA) to overcome the shortcomings faced by ELISA based on iron oxide nanoparticles (IONPs) (Fig. 6a). To this end, IONPs were functionalized by the surface engineering of a simplified CRP antibody, which constructed IONP probes for CRP labeling. The capture antibody immobilized on solid supports recognizes and captures the CRP molecules in biological samples, followed by the addition of IONP probes to label the captured CRP molecules; the unbound IONP probes are then removed and the bound IONP probes dissolved through acid lysis. The amounts of iron ions released could reveal the relative contents of the CRP, which could be quantified by a colorimetric strategy. Compared with ELISA, ILISA contributed to a simpler and more rapid method whose sensitivity could reach the subpicomolar level. Similarly, improved diagnostic efficiency can be achieved by introducing other metal oxide nanocrystals, such as zinc oxide nanocrystals, that were previously adopted to construct nanosurfaces for the highly ordered display of CRP antibody with an LOD of < 10 ng mL^−1^ (Fig. 6b) [145]. More importantly, the nanosurface-based nanodiagnostic platform is easy to fabricate inexpensively [145], which implies excellent possibilities for clinical translation. Alternatively, the incorporation of copper oxide (CuO) nanocrystals could improve the performance of zinc oxide (ZnO) nanocrystal-based biosurfaces by potentiating redox properties and electron transfer and decreasing band gap energy [143, 172]. For instance, biosurfaces fabricated by ZnO–CuO hybrid nanomaterials with a volume ratio of 1:2 exhibited a dramatically enhanced signal for CRP detection in comparison with pure ZnO-based biosurfaces (Fig. 6c) [143]. Consequently, more accurate, time-saving and adaptive methods for clinical applications can be developed by precisely designing and optimizing the formulations of nanocrystal-based diagnostic platforms, including nanocrystal species, multiple components, sizes, and ratios. In addition to colorimetric signals, some nanomaterials with inherent physicochemical properties can transform immune signals into other readout signals, such as fluorescent signals, electrochemical signals, and other visible signals. Polyclonal anti-CRP (pAb-CRP) can be immobilized on the surface of fluorescent nanomaterials, such as tetraethylene glycol-conjugated fullerene nanoparticles (C_60_-TEG), via simple two-step reactions (Fig. 7a) [146]. The resultant nanoprobe pAb-CRP-C_60_-TEG recognizes the CRP molecules on the lateral flow strip based on an immunochromatographic assay (Fig. 7b) and exhibits fluorescent signals displaying the corresponding band responses to different concentrations of CRP (Fig. 7c). Integration of gold nanorods (GNR) into voltammetry detection systems can improve the sensing performance on CRP through a large surface area anti-CRP display that sufficiently facilitates antigen–antibody recognition (Fig. 7d) [147]. With the help of GNR, the voltammetry nanosystem decreases the LOD of CRP to 10 fM, which is 10,000,000-fold lower than that of ELISA (100 nM) [147]. In addition, citrate-stabilized gold nanoparticles have been adopted to surface display anti-CRP (Fig. 7e), thus obtaining anti-CRP gold nanoconjugate (GNC) probes that were introduced to develop an ultrasensitive vertical flow immunokit (VFIK) for CRP quantification [148]. Based on the similar immunosandwich reactions on the VFIK device, the appearance of two red dots represents the existence of CRP (Fig. 7f), and the intensities of the red dots show a positive correlation to the different concentrations of CRP (Fig. 7g). Valuably, the GNC-based VFIK could achieve simple and fast detection for within 2 min [148], which satisfies the early warning principle for sepsis management.

### Nanodiagnostic Platforms that Detect PCT for the Identification of Sepsis

PCT is a thyroid-yielded polypeptide released in response to infection with a high serum concentration in patients with sepsis and infections, representing a reliable biomarker of severe bacterial infections for sepsis differentiation [173, 174]. Clinical inclusion of PCT can effectively guide antibiotic treatments, thereby avoiding possible generation of drug resistance and benefiting therapeutic outcomes [175]. Similarly, to overcome the limitations impeding clinical PCT assays such as immunoluminometric and chemiluminescence strategies, nanodiagnostic platforms are introduced for the rapid, simple, sensitive and low-cost detection of PCT.

**Fig. 8:**
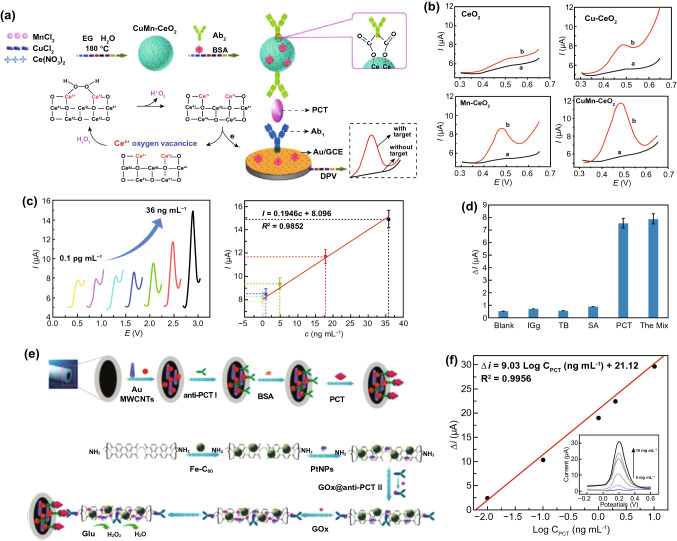
Cu/Mn double-doped CeO_2_ nanocomposites contributing to sensitive electrochemical detection of PCT via signal amplification. **a** Proposed mechanism of signal amplification provided by the CuMn–CeO_2_ nanocomposite immunosensor for PCT detection. **b** DPV responses of the proposed immunosensor incubated with PCT using Ab2/BSA/CeO_2_, Ab2/BSA/Cu–CeO_2_, Ab2/BSA/Mn–CeO_2_ or Ab2/BSA/CuMn–CeO_2_ as a signal tag in the absence of H_2_O_2_ (curve a) and in the presence of H_2_O_2_ (curve b). **c** DPV responses of the CuMn–CeO_2_ nanocomposite immunosensor after incubation with different concentrations of PCT (left), calibration curve of the intensity current of the immunosensor with Ab2/BSA/CuMn–CeO_2_ (right). **d** DPV current response of five samples detected by the CuMn–CeO_2_ nanocomposite immunosensor for verifying the sensitivity of PCT detection. Reproduced with permission from Ref. [151]. Copyright 2017 American Chemical Society. C_60_ carboxyfullerene-based functionalized nanohybrids for ultrasensitive electrochemical detection of PCT as a signal-amplifying tag. **e** Schematic illustration of the fabrication of the C_60_ carboxyfullerene-based functionalized nanohybrid immunosensor and the corresponding catalysis amplifying principle. **f** DPV responses of the proposed nanosensor contribute to a favorable linear relationship with the logarithm of PCT concentrations. Reproduced with permission from Ref. [150]. Copyright 2015 Elsevier

Specifically, increasing advances have been achieved in nanomaterials with robust catalytic activity. Nevertheless, most of these nanomaterials fail to directly be used as redox probes to create electrochemical biosensors for biomedical assays because the redox signal of these nanomaterials can be triggered and read only in strong acid or alkali solutions at high positive or negative potential, dramatically restricting their clinical translation [151]. In this case, Yang et al. [151] reported a Cu/Mn double-doped CeO_2_ (CuMn–CeO_2_) nanocomposite that contributes to signal amplification for the precise electrochemical detection of PCT (Fig. 8a). MnCl_2_, CuCl_2_ and Ce(NO_3_)_2_ were employed to synthesize CuMn–CeO_2_ nanocomposites, and then the detected antibody for PCT (Ab_2_) was immobilized on the surface of CuMn–CeO_2_ via simple ester-like bridging. After PCT in the sample was recognized and immobilized by a capture antibody (Ab_1_)-functionalized Au/GCE chip, the addition of Ab_2_-immobilized CuMn–CeO_2_ nanoprobes in the presence of H_2_O_2_ can produce and amplify redox signals for PCT characterization (Fig. 8a). Mechanistically, the introduction of Cu and Mn into CeO_2_ lattices will generate extra oxygen vacancies, thus exhibiting superior catalytic activity for promoting electron transfer. The results indicated that CuMn–CeO_2_ amplified redox signals more effectively than CeO_2_, Cu–CeO_2_, and Mn–CeO_2_ (Fig. 8b). The signals increased with gradually increasing concentrations of PCT from 0.1 to 36 pg mL^−1^, exhibiting a positive linear relationship (Fig. 8c), simultaneously with a low LOD of 0.03 pg mL^−1^. Furthermore, the CuMn–CeO_2_ nanocomposites-based biosensor could specifically identify and quantify the PCT in presence of other interference proteins including thrombin (TB), hemoglobin (IGg) and streptavidin (SA) (Fig. 8d). For electrochemical signal amplification, Li et al. [150] proposed another interesting nanoplatform, C_60_ carboxyfullerene-based functionalized nanohybrids, to support a more ultrasensitive electrochemical immunosensor for PCT detection (Fig. 8b). In detail, multiwalled carbon nanotubes (MWCNTs) and AuNPs were cointegrated into GCE systems that subsequently immobilized with primary PCT antibody which constructed an anti-PCT I/AuNP@MWCNT/GCE immunosensor capable of capturing PCT in samples (Fig. 8e). Hydrophilic C_60_ carboxyfullerene linked to the redox probe ferrocene carboxylic acid (Fc) was used to synthesize Fc-C_60_ nanocomposites which were attached with platinum nanoparticles (PtNPs) with excellent electrocatalytic activity (Fig. 8e). The resultant PtNPs-Fc-C_60_ nanohybrids further immobilized glucose oxidase (GOx)-labeled secondary PCT antibodies (anti-PCT II) to generate GOx@anti-PCT II-PtNP-Fc-C_60_ nanohybrids as nanoprobes that could detect the captured PCT by electron transfer. As expected, a favorable linear relationship was established by the proposed immunosensor with LOD of 6 pg mL^−1^ (Fig. 8f). By taking advantage of these nanomaterials capable of manipulating redox reactions and favorably displaying detection antibodies to amplify readout signals, the LOD of diagnostic devices for PCT monitoring would be decreased significantly, which was suitable for advancing clinical diagnostic technology.

**Fig. 9:**
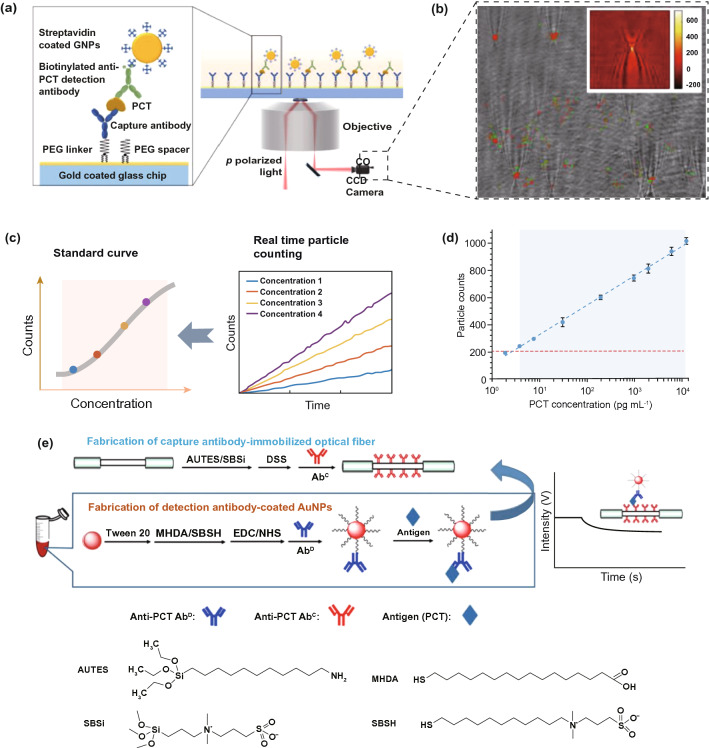
Nanodiagnostic platforms based on nanoplasmonic technology for PCT detection. TD immunoassay. **a** Construction and mechanisms of TD immunoassay for PCT detection. **b** Representative image of TD immunoassay for PCT detection using nanoplasmonic imaging. **c** The signal transduction from real-time particle counting to PCT concentrations can then be employed to draw standard curve. **d** Standard curve of PCT detection generated by TD immunoassay showing a favorable linear relationship between particle counts and PCT concentrations. Reproduced with permission from Ref. [154]. Copyright 2019 American Chemical Society. **e** Fiber optic nanogold-linked immunosorbent assay (FONLISA): fabrication of capture platforms and nanoplasmonic probes. Reproduced with permission from Ref. [152]. Copyright 2020 Elsevier

Nevertheless, nanodiagnostic platforms that achieve both fast/real-time detection and ultrasensitive accuracy are urgently needed to satisfy clinical requirements. Although PCT has been considered one of the most accurate biomarkers for the diagnosis of sepsis, its detection usually delays the 2–4-h gold treatment time after the occurrence of sepsis, which is lethal for many patients worldwide [174, 176]. In this case, nanoplasmonic technology has been adopted to improve the response speed while maintaining detection accuracy [177]. Jing et al. [154] designed a time-resolved digital immunoassay (TD immunoassay) that supported the rapid and sensitive detection of PCT through nanoplasmonic imaging technology (Fig. 9a). In detail, the gold-coated glass chip was functionalized by the immobilization of the capture antibody as a capture platform; after being captured by a gold-coated glass chip, PCT molecules were recognized by biotinylated anti-PCT detection antibody to form a sandwich-like complex, followed by incubation with streptavidin-coated gold nanoparticles (GNPs) that possess potent nanoplasmonic absorption and fine-tunable optical features of particle plasmon resonance, to label the bounded detection antibody via biotin-streptavidin reaction; then, p-polarized light was directed onto the sensor surface to excite the plasmons of the GNP surface (Fig. 9a), and the resultant reflected light could be collected and imaged by CCD (Fig. 9b). By using this TD immunoassay, the concentration of PCT in biological samples could achieve real-time quantification by counting particles (Fig. 9c). The readout signals (GNP counts) of the TD immunoassay showed a favorable linear relationship with the series of concentrations of PCT (Fig. 9d). The TD immunoassay contributed to an ultralow LOD of ~ 2.8 ng mL^−1^ for a total detection time of ~ 25 min, which is significantly superior to clinical methods. In addition, another nanoplasmonic platform-based device, fiber optic nanogold-linked immunosorbent assay (FONLISA), proposed by Chiang et al. [152] (Fig. 9e), exhibited a sufficiently low LOD of 7.3 fM and response time of < 15 min. FONLISA used detection antibody-coated gold nanoparticles (AuNPs) as detection probes and capture antibody-immobilized optical fibers as capture probes (Fig. 9e). To reduce background noise and enable biofunctionalization and antibiofouling properties, both AuNPs and optical fibers were coated with a mixed self-assembled monolayer composed of 16-mercaptohexadecanoic acid (MHDA) and sulfobetaine silane (SBSi) or 11-aminodecyltrethoxysilane (AUTES) and sulfobetaine thiol (SBSH), respectively (Fig. 9e). Contrary to conventional fiber optic particle plasmon resonance (FOPPR) biosensors, integrating plasmonic nanomaterial probes (AuNPs) into FONLISA dramatically amplifies the electrochemical signals, thereby decreasing the LOD for PCT detection. Another strategy based on the immune sandwich assay employs a reduced graphene oxide (rGO)–gold (Au) nanocomposite film as the sensing platform, which exhibits a large surface-to-volume ratio to increase the amount of PCT detection antibody immobilization and utilizes single-walled carbon nanohorn (SWCNH)/hollow Pt chain (HPtC) complexes as detection probes, which are synthesized through coimmobilizing detection antibodies, HPtCs and horseradish peroxidase (HRP) onto the surface of SWCNHs [153]. HPtCs and HRP can synergistically catalyze H_2_O_2,_ thereby contributing signal amplification to readout. Given the synergistic improvement effects, a novel nanodiagnostic platform named the Porous Layer Open Tubular-Signal Amplification (PLOT-SA) sensor integrates a streptavidin–biotin signal amplification (SA) system with a porous layer open-tube (PLOT) capillary column, which combines the advantages of the enhanced immobilization of capture anti-PCT antibody and signal amplification, achieving an LOD of 0.01 pg mL^−1^ [155]. Furthermore, combining matrix nanospotting technology and lateral flow immunoassays has been shown to simplify the procedure and maintain sensitivity [156].

### Nanodiagnostic Platforms that Detect Cytokines for the Identification of Sepsis

Sepsis involves complex immune responses that result in the biogenesis, secretion and biodistribution of versatile cytokines [178]. Both proinflammatory and anti-inflammatory events coexist with innate immune and adaptive immune disorders during sepsis [179]. In the early phase, cytokine storms triggered by dangerous molecules (e.g., PAMPs and DAMPs) contributed to the concomitant occurrence of excessive inflammation [13, 179]. However, pathogenetic alterations such as lymphocyte exhaustion and LPS tolerance at the late stage cause a significant decrease in cytokine production, which further leads to the impairment of immune functions and subsequent uncontrollable growth of invaders. Consequently, monitoring the levels of cytokines represents a reliable strategy to characterize sepsis progression, which can guide clinicians to adopt precision and adaptive therapeutics.

**Fig. 10:**
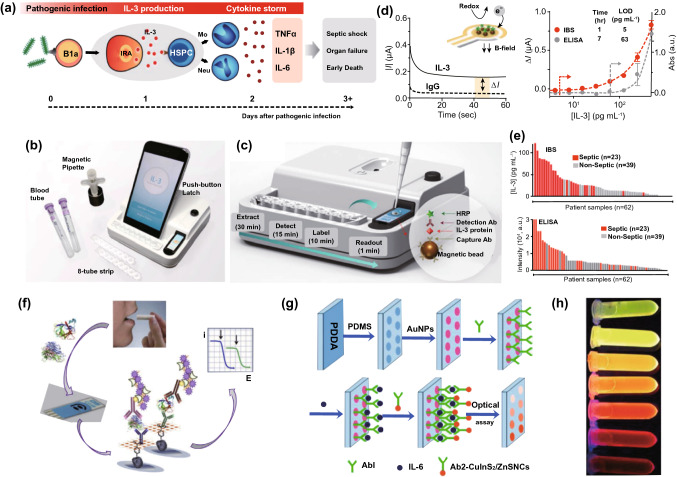
Integrated biosensor for rapid point-of-care sepsis diagnosis by detecting IL-3. **a** IL-3-mediated mechanism of sepsis. **b** Photograph of the whole integrated biosensor system. **c** Detection steps and principle of the integrated biosensor for IL-3 detection. **d** Electrical currents are generated on or near the electrode via TMB redox reaction catalyzed by HRP-coated magnetic beads, varying concentrations of IL-3-containing human plasma were assayed by IBS and ELISA. **e** Samples from septic (*n* = 23) and nonseptic patients (*n* = 39) were analyzed by integrated biosensor and ELISA. Reproduced with permission from Ref. [163]. Copyright 2018 American Chemical Society. **f** Dual screen-printed electrodes modified with functionalized double-walled carbon nanotubes for the simultaneous detection of IL-1β and TNF-α in serum and saliva. Reproduced with permission from Ref. [160]. Copyright 2017 Elsevier. Color-tunable CuInS_2_/ZnS nanocrystals for IL-6 detection. **g** Schematic mechanism of the proposed nanosensor for IL-6 detection. **h** Photographs of CuInS_2_/ZnS NCs with various ratios of Cu/In (bottom 3:4, 1:2, 3:8, 1:3, 1:4, 1:5, and 1:10 top) under excitation at 365 nm UV. Reproduced with permission from Ref. [157]. Copyright 2013 American Chemical Society

Upon stimulation by risk factors (e.g., pathogens, PAMPs, and/or DAMPs), IL-3 is released by innate response activator (IRA) B cells to operate downstream of a series of cytokines, such as IL-1β, IL-6, and TNF-α, causing subsequent cytokine storm and multiple organ failure leading to a fatal outcome (Fig. 10a) [163]. Min et al. [163] theorized that IL-3 might be an independent predictor of sepsis and septic shock. However, admitting IL-3 into clinically practical trials is restricted by the lack of fast, user-friendly, and highly accurate detectors. Hence, they developed a point-of-care integrated biosensor for sepsis (IBS) for fast and accurate identification, thereby enabling timely treatment (Fig. 10b) [163]. The IBS comprises four steps (Fig. 10c): (i) the IL-3 in biological samples was initially enriched by capture antibody-modified magnetic beads and then separated by magnetic pipet; (ii) the enriched IL-3 was recognized by detection antibody; (iii) the captured IL-3 was labeled with oxidizing enzyme (horseradish peroxidase, HRP); and (iv) finally, the IL-3-enriched beads were mixed with chromogenic electron mediators (3,3′,5,5′-tetramethylbenzidine, TMB) that were oxidized through HRP catalysis by receiving electrons from the electrode (Fig. 10d left), thus generating electrical current as an electrochemical readout signal. In this case, the chronoamperometry method was adopted for signal detection, in which the enhanced current level (Δ*I*) between IL-3 and the IgG controls is the analytical metric (Fig. 10d left). Compared with gold standard ELISA, IBS achieved > 10 times greater sensitivity (LOD < 10 pg mL^−1^) with > 5 times faster speed (Fig. 10d right). When subjected to clinical study, IBS also differentiated septic from nonseptic patients more accurately than ELISA according to the IL-3 level in blood (Fig. 10e). Further clinical study using the IBS platform demonstrated that an IL-3 concentration of > 24 pg mL^−1^ is critically correlated with the multiorgan failure and mortality of septic patients, suggesting that IL-3 could serve as an independent biomarker to facilitate early warning of sepsis. For typically characterized cytokines, such as TNF-α, IL-1β, and IL-6, which are involved in the response to a heterogeneous inflammatory network in the sepsis microenvironment, convenient, fast and accurate detection can also be achieved by nanomaterial-inspired platforms to enable early diagnosis. For instance, functionalized double-walled carbon nanotubes were employed to modify carbon electrodes as scaffolds to dramatically enhance the lifetime and accuracy of electrochemical immunosensors, achieving LODs of 0.38 pg mL^−1^ for IL-1β and 0.85 pg mL^−1^ for TNF-α (Fig. 10f) [160]. Based on the same principle as immunoarrays, the inorganic metal nanoparticle CuInS_2_/ZnS was conjugated to the nonrecognized region of the detected antibody for IL-6 characterization via photoluminescence readout (Fig. 10g), in which CuInS_2_/ZnS became visible in the near-infrared through changing the initial ratio of Cu/In in the precursors (Fig. 10h) [157].

**Fig. 11:**
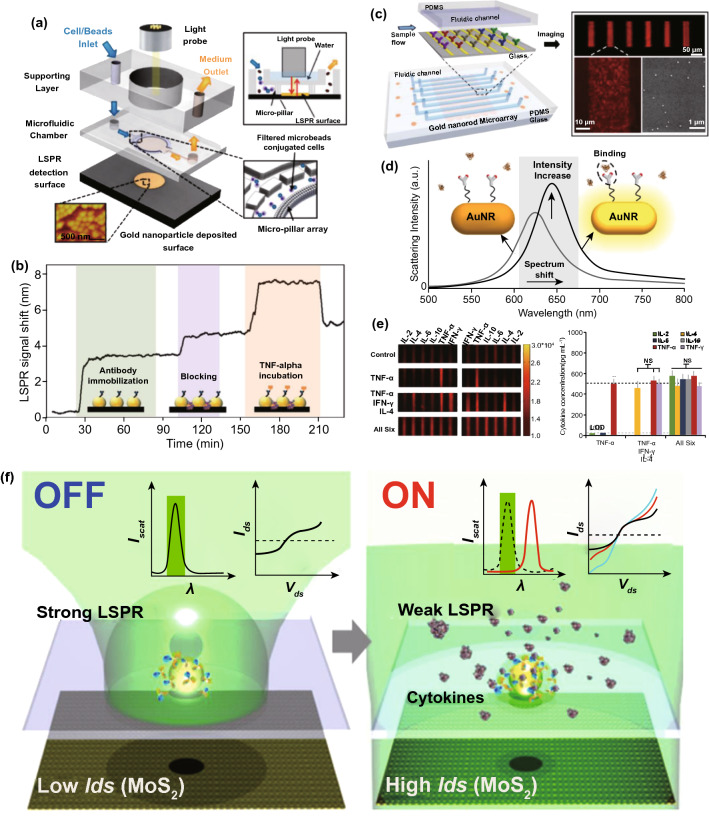
Nanodiagnostic platforms based on nanoplasmonic technology for rapid, ultrasensitive, and/or real-time monitoring of cytokines. Optofluidic LSPR nanoplasmonic detector. **a** Schematic illustration of the organization of the optofluidic LSPR nanoplasmonic detector. **b** Real-time LSPR signal shift of the nanoplasmonic detector when sensing target molecules (TNF-α). Reproduced with permission from Ref. [161]. Copyright 2014 American Chemical Society. Multiplex LSPR nanoplasmonic biosensor. **c** Schematic illustration indicating the organization and detection mechanisms of the multiplex nanoplasmonic biosensor. **d** LSPR signal shift of antibody-coated AuNRs before or after the attachment of target cytokines. **e** Dark-field nanoplasmonic imaging and multiplex cytokine quantification of the multiplex nanoplasmonic biosensor microfluidic channels undergoing different sample mixtures of various cytokines. Reproduced with permission from Ref. [164]. Copyright 2015 American Chemical Society. **f** Biotunable nanoplasmonic filter combined with photoconductive materials for IL-1β detection. Reproduced with permission from Ref. [162]. Copyright 2017 American Chemical Society

Similar to the detection of PCT, nanoplasmonic technology has also been employed to resolve the challenges impeding conventional methods and has achieved the rapid, ultrasensitive, real-time, and/or multiplex detection of various cytokines [159, 161, 162, 164, 177, 180]. To support label-free, real-time, and accurate detection and characterization of immune cellular functions, Oh et al. [161] aimed to develop a localized surface plasmon resonance (LSPR)-based nanoplasmonic device that, however, was limited by insufficient sensitivity and cell sorting capability. Nevertheless, integrating an optofluidic platform in an LSPR-based nanoplasmonic sensor resolved these issues by constructing an LSPR nanoplasmonic optofluidic platform that allowed the trapping, sorting and stimulation of target immune cells for cytokine secretion assays (Fig. 11a) [161]. The nanodiagnostic platform comprises four parts: a light probe, a supporting layer, a microfluidic chamber and an LSPR detection surface (Fig. 11a). The mixture of cell beads was injected into the inlet and then underwent cell sorting and enrichment mediated by the microfluidic chamber (Fig. 11a). The target cells could be stimulated and monitored by the nanoplasmonic effects of antibody-coated AuNPs for the expression of target cytokines (Fig. 11b). This LSPR nanoplasmonic optofluidic device successfully detected as few as 1000 molecules of cell-secreted TNF-α, which was 100 times lower than that of conventional methods, and the total assay time was only 4–5 h, which was 3 times shorter than that of ELISA. Based on analogous concepts, Chen et al. [164] established a multiplex serum cytokine immunodetector by integrating nanoplasmonic microarrays (Fig. 11c), which could simultaneously quantify diverse serum cytokines, including IL-2, IL-4, IL-6, IL-10, TNF-α, and IFN-γ. The multiplex nanoplasmonic sensor was composed of eight parallel microfluidic channels running orthogonal to six meandering stripes coated with six cytokine antibody-functionalized gold nanorods (AuNRs) (Fig. 11c). After the attachment of target biomolecules, nanoplasmonic material AuNRs could generate a redshift and scattering intensity change in the plasmonic resonance without particle–particle electromagnetic interferences (Fig. 11d), which were therefore recorded by dark-field imaging (Fig. 11c), consequently achieving the real-time monitoring of multiplex cytokines. In terms of these advantages, the multiplex nanoplasmonic sensor capable of identifying corresponding cytokines in samples with different components (Fig. 11e) paves a broad avenue in monitoring immune alterations and organ failure, which possibly revolutionizes the theranostic strategies of sepsis. Nevertheless, more sophisticated concepts have been proposed to overcome the limitations impeding LSPR and thus facilitate the clinical application of nanoplasmonic technology in cytokine detection. For the optical nanoplasmonic platform, the strong LSPR effect induced by antibody-conjugated AuNPs greatly attenuates the delivery of incident light to photoconductive devices such as MoS_2_ flakes, thus generating a low photocurrent (Fig. 11f OFF) [162]. However, the attachment of target biomolecules (e.g., IL-1β) onto the surface of AuNPs would shift the LSPR wavelength, exhibiting a weak LSPR effect that permits the enhanced transmission of incident light to the surface of the MoS_2_ flake, which thereby generates a high photocurrent for readout whose strength depends on the concentrations of target molecules (Fig. 11f ON) [162]. Such a biotunable nanoplasmonic optical sensor contributes an LOD of 14 fM and an assay time of 10 min to the detection of IL-1β [162]. Interestingly, utilizing a trehalose glycopolymer as a resist on silicon substrates allows the regular pattern of antibodies (e.g., anti-IL-6 and anti-TNF-α) to be achieved through electron beam lithography [159]. By performing sandwich immunoassays using antibody-coated AuNPs as detection probes, cytokines such as IL-6 and TNF-α could be directly written onto the surface of the capture platform and then visualized by the LSPR effect mediated by AuNPs with dark-field microscopy [159].

Collectively, immunoassays combined with electrochemical nanotechnology and nanoplasmonic techniques significantly advance the development of cytokine detection. Of course, other smart nanodiagnostic concepts have been continuously proposed for cytokine monitoring. For instance, the integration of antibody-functionalized nanobeads into a microgel matrix capable of volume phase-transition change supports an enzyme-free immunoassay with a fivefold increase in signal-to-noise ratios [165]. Such a nano-in-micro smart gel composite has been effectively applied in the detection of IL-6, IL-8, and MCP-1 [165]. In addition, Hao et al. [158] adopted an aptameric nucleic acid rather than an antibody immobilized on graphene to detect IL-6. The aptameric graphene-based nanosensing system avoided the shortcomings of immunoassays, such as the high costs of antibody synthesis [158]. In terms of these advances in cytokine detection, we believe that in the future, nanodiagnostic technology will assist clinicians in more efficiently mapping the immune atlas in patients with sepsis, which will completely advance our understanding and therapeutic modes of sepsis.

In summary, because of their versatile nanoscale characteristics, including optical controllability, large surface energy, and magnetism, nanomaterials present encouraging prospects for sepsis diagnosis, potentially contributing to increasing therapeutic levels worldwide in the future.

## Nanotherapeutic Platforms for Sepsis Treatment through Targeting Pathogenesis

Sepsis involves multiorgan dysfunction, which mainly results from systemic infection and uncontrollable immune disorder. Consequently, it is rather difficult to develop precision therapeutics for sepsis, and the elimination of infection and restoration of immune homeostasis are still the two major trends in sepsis management. However, the occurrence of drug-resistant bacteria and the lack of effective strategies to achieve immune modulation in the ICU pose severe problems. The use of nanotechnology to create and utilize nanoscale drugs and nanocarriers may provide unique insight into resolving these problems in the future. In this section, a series of nanotherapeutics capable of controlling bacterial infections and/or restoring immune homeostasis for sepsis therapy are elucidated in detail.

### Nanotherapeutic Platforms Rescue Sepsis by Targeting Bacterial Infections

It is acknowledged that infection represents the most important contribution to sepsis. The more rapid the elimination of infection is, the more favorable an outcome of sepsis can be achieved. However, the usage of large amounts of traditional antibiotics in patients with sepsis can lead to the occurrence of drug resistance, finally causing therapeutic failure. Nanomedicines that involve direct bacterial killing with low resistance, bacterial-blood separation, or the restoration of the innate immune defense to disrupt bacteria provide unique insights into the future of sepsis management.

#### Brief Summary of Antimicrobial Nanoplatforms

##### Inorganic/Metallic NPs-Based Antimicrobial Platforms

It is commonly acceptable that NPs are desirable carriers for antimicrobial delivery, some of which are bactericidal directly. Inorganic/metallic NPs, such as AuNPs, AgNPs, and superparamagnetic iron oxide NPs, represent the most used antimicrobial materials due to their ease of surface chemistry, fine-tunable characteristics and inherent optical/magnetic effects [181]. AuNPs as an ideal carrier have been employed to deliver antibiotics (e.g., ampicillin and amoxicillin), and the antibiotics are adsorbed onto surface thus allowing targeted delivery [182]. By taking advantage of proper surface chemistry and inherent nanoplasmonic effect, Au-based nanoplatforms achieved photobactericidal activity against both Gram-negative and Gram-positive bacteria through producing ROS [183]. In fact, AuNPs can directly kill bacteria (*e.g., Pseudomonas aeruginosa* and *Staphylococcus aureus*) through inducing lethal bacterial membrane-tension alterations [184]. One of the most representational nanomaterials with intrinsic antibacterial activity is AgNPs which can eradicate broad-spectrum bacteria even including multidrug-resistant strains and have already been developed as antimicrobial dressings for wound management [185, 186]. The mechanisms responsible for bactericidal effect of AuNPs involve direct membrane damage, interaction with membrane proteins, detachment of bacteria wall, ROS production, induction of abnormality in DNA and RNA replication [181, 187]. Interestingly, superparamagnetic iron oxide NPs can trigger potent antibacterial activity under exogenous electromagnetic stimulation, because of their magnetic hyperthermia property (Javanbakht et al., Plos One, 2016).

##### Polymeric NPs-Based Antimicrobial Platforms

Natural and synthetic polymeric nanomaterials have drawn considerable attention in eradicating bacterial infection because of their potential in antibiotic delivery and combatting drug resistance [181, 188]. Due to nontoxicity, controlled release, and ease of structural modification, biopolymers, such as chitosan and alginate, have already been investigated as adaptive antibiotic carriers [188–192]. Chitosan is a polycationic polysaccharide and thus contains numerous positively charged groups that allow mucoadhesion and penetration of biomembranes. Codelivering black phosphorus quantum dots (BPQDs) and antibiotic amikacin using chitosan NPs enables adhesion to mucous membrane, and then allows the autoxidation of BPQDs to drive antibiotic release for therapy of chronic obstructive pulmonary disease (COPD) [193]. Owing to its abundant positive charges, potential toxicity should be taken into account when designing chitosan-based nanoplatforms for biomedical uses. Contrary to chitosan, alginate is a negatively charged polysaccharide which self-assemble into hydrogel in presence of Ca^2+^ [194]. Hence, alginate exhibits excellent safety and has widely been used as hydrogel dressing for controlled release of antimicrobials [181, 194]. For example, alginate aerogel could effectively prolong the release of tigecycline and octahedral Cu crystal and maintain the antibacterial effect over 18 days and simultaneously show low biological toxicity, thus providing an improved pharmacodynamics for treatment of osteomyelitis [195]. With regard to synthetic nanomaterials, PLGA, poly(malic acid), and pluronic as well as derivatives have already been designed to biocompatible NPs for drug delivery. However, some synthetic nanomaterials themselves possessing bactericidal activity are of particular interests, because these nanomaterials naturally integrate both functions of nanoplatforms and antimicrobials. By attaching terminal modifications and adjusting ratio of cationic and hydrophobic residues, amphiphilic antimicrobial peptides can be established, which can self-assemble into supramolecular nanostructures and exhibit antimicrobial activity; another example is positively charged amphiphiles which are composed of one or more positively charged head group(s) (e.g., quaternary amine) and hydrophobic tail(s) (e.g., fatty acid chain). Both of the aforementioned antimicrobial nanomaterials can effectively disrupt bacteria and even combat biofilm and resistance; however, potential systemic toxicity hinders in vivo applications.

##### Liposomes-Based Antimicrobial Platforms

Liposomes, the most representational lipid-based NPs, consist of biomembrane-like phospholipid bilayers that confer membrane affinity and allow favorable drug encapsulation for both hydrophilic and hydrophobic agents. Benefiting from these advantages, liposomes can overcome host biomembrane barriers or biofilms generated by multidrug-resistant strains, delivering antimicrobial agents directly to bacteria [196, 197]. In addition, liposomes have been reported to successfully combat the outer membrane barrier of Gram-negative bacteria and deliver antibiotics into bacterial cells for significantly improved activity [198]. Being supplemented with surface chemistry and/or other nanomedical techniques enables liposomes to be smart-responsive nanoplatforms which provide controlled, on-demand, and/or spatiotemporal release of antimicrobials. Pang and coworkers designed a bacteria-responsive nanoliposomes which were composed of phospholipids and maltohexaose-decorated cholesterol, for delivery of sonosensitizer—purpurin 18 [199]. Such a maltohexaose-functionalized nanoliposomes could actively target bacteria through recognizing bacteria-specific maltodextrin transport pathway; once entering IME, nanoliposomes were degraded by bacteria-oversecreted phospholipase A_2_ (PLA_2_), thus initiating the release and internalization of purpurin 18 into bacteria, which consequently contributed to visualized sonodynamic therapy via near-infrared imaging and producing toxic ROS. Wu and coworkers coincorporated rifampicin and calcium peroxide (CaO_2_) into a liposome-based platform and thereby obtained a bacterial toxin-triggered cascade nanoreactor [200]. The nanoreactors could be embedded by bacterial toxins to form pores once encountering bacteria, and H_2_O molecules could permeate into inner core of nanoreactors to react with CaO_2_ and then generate H_2_O_2_. Resultant H_2_O_2_ decomposed to O_2_ which subsequently power the release of rifampicin for direct bactericidal effect. In vivo efficacy of this cascade nanoreactors was validated in wound healing models complicated with MRSA infection, implying a promising application in advanced wound management.

##### Biomimetic NPs-Based Antimicrobial Platforms

Nature-inspired biomimetic nanoplatforms with optimized surface biophysicochemical properties can overcome shortcomings and/or afford unachievable functions of conventional synthetic NPs, thus contributing to unique advantages in drug delivery, vaccine development, and detoxification, etc*.* [102]. Consequently, unmet medical challenges in infectious diseases can be resolved by biomimetic nanoplatforms [201]. A typical example is RBC membrane-coated NPs which as stealth vehicles can prolong the circulation time and improve pharmacokinetic behaviors of antimicrobial agents [104]. Besides, RBC layer allows nanoplatforms to competitively adsorb bacterial toxins for attenuating toxin-induced hemolysis [110]. Immune cell membrane-coated NPs have reported to target IME, which can be employed for targeted delivery of antibiotics [202]. Intrinsic components in immune cell membrane coating, including proteins, lipids, and some metabolites, might be capable of modulating immune responses. Another strategy is isolating bacteria-derived materials including components of bacterial membrane (e.g., surface layer proteins), bacterial membrane, and bacterial membrane vesicles to decorate NPs. The obtained bacteria-mimicking NPs can favor antibiotic delivery or block bacterial invasion. Huang and coworkers found *Acinetobacter baumannii* evolved an interesting drug-resistant mechanism that eliminates antibiotics using bacterial outer membrane vesicles (OMVs) [203]. Inspired by this phenomenon, they employed sub-MIC dose of antibiotics to stimulate *Acinetobacter baumannii* and then isolated the antibiotic-containing OMVs by ultracentrifugation. In a mouse model of intestinal infection, these OMVs significantly prolonged the retention time of levofloxacin in intestinal tract for 36 h, and reduced bacterial burden in the small intestine and feces. To eradicate intracellular *S. aureus*, Gao and workers isolated extracellular vesicles (EVs) secreted by *S. aureus* as antibiotic carrier to test if this *S. aureus*-mimicking strategy could achieve intracellular delivery of antibiotics [204]. As expected, *S. aureus*-secreted EVs were internalized at a higher performance by *S. aureus*-infected macrophages than by sterile counterparts, whereas the PEGlyated liposomes and *E. coli*-derived OMVs could not be effectively engulfed by *S. aureus*-infected cells; instead, *E. coli*-derived OMVs, but not *S. aureus*-secreted EVs and liposomes, shows distinctly selective uptake by *E. coli*-infected macrophages. These results suggest that bacterial membrane vesicles-based nanoplatforms can be highly selective, which specifically recognize infected cells and further combat pathogens via releasing antibiotics. Such an intelligent nanoplatform is very suitable for sepsis management in our opinion and can be developed as a specifically nanobiotic.

Although numerous NPs-based antimicrobial platforms have been proposed, only a small amount of them has been evaluated in septic models. We think there are two major reasons: (1) sepsis is a highly heterogenic syndrome complicated with multiple abnormalities besides bacterial infections, which significantly increase difficulties of developing effective and adaptive antimicrobial nanoplatforms; (2) despite high mortality in ICU, the incidence of sepsis is significantly lower than cancer and cardiovascular diseases, usually novel technologies are preferentially applied in diseases with high global incidence. Consequently, to advance the development of nanotechnology in sepsis management, we next describe the recent advances of antimicrobial nanoplatforms that have been evaluated in septic models.

#### Antimicrobial Nanoplatforms for Sepsis Management

Meropenem, one of the strongest commercial antibiotics, belongs to the carbapenem class and has already been applied in the ICU for sepsis and pneumonia therapy [205–208]. However, its short half-life usually requires repeated administration of a high dosage, which is prone to inducing drug resistance [205, 209]. Nevertheless, encapsulating meropenem into chitosan nanoparticles could dramatically improve its pharmacokinetic properties, thus resolving these challenges [209]. The resultant meropenem-loaded nanoparticles retain the potent bactericidal activity of the free drug against bacteria, including *S. aureus*, MRSA, *E. coli*, and *K. pneumoniae* [209]. Preclinical experiments demonstrated that meropenem-loaded nanoparticles rescued 100% of *K. pneumoniae*-induced septic mice, while only 70% of free drug-treated mice survived [209]. Hence, designing a suitable drug delivery system to remedy the shortcomings impeding traditional antibiotics might be an effective and practical method to recover therapeutic activity. Despite their promise for the treatment of sepsis and other infectious diseases, most antimicrobial peptides (AMPs) are still limited by their poor pharmaceutical properties, such as poor solubility and short half-life [210]. Clavanins, a type of AMP isolated from marine animals, could be formulated with a methacrylate nanocarrier consisting of EUDRAGIT®L 100–55 and RS 30 D solution (3:1 w/w) [211]. With delivery by methacrylate nanocarriers, clavanins successfully rescued 100% of sublethal polymicrobial sepsis, which resulted from the improvement in pharmaceutical properties [211]. Additionally, lung delivery of the nonnatural AMP SET-M33 using single-chain dextran nanoparticles could distinctly improve the lung biodistribution and prolong the half-life of SET-M33, thus effectively eliminating *P. aeruginosa* in acute lung sepsis [212]. Consequently, employing well-designed nanocarriers to formulate antimicrobials can benefit therapeutic efficiency through targeting delivery, modulating biodistribution, improving bioavailability, and prolonging half-life, which might partly avoid the generation of drug resistance at the late stage of sepsis.

**Fig. 12:**
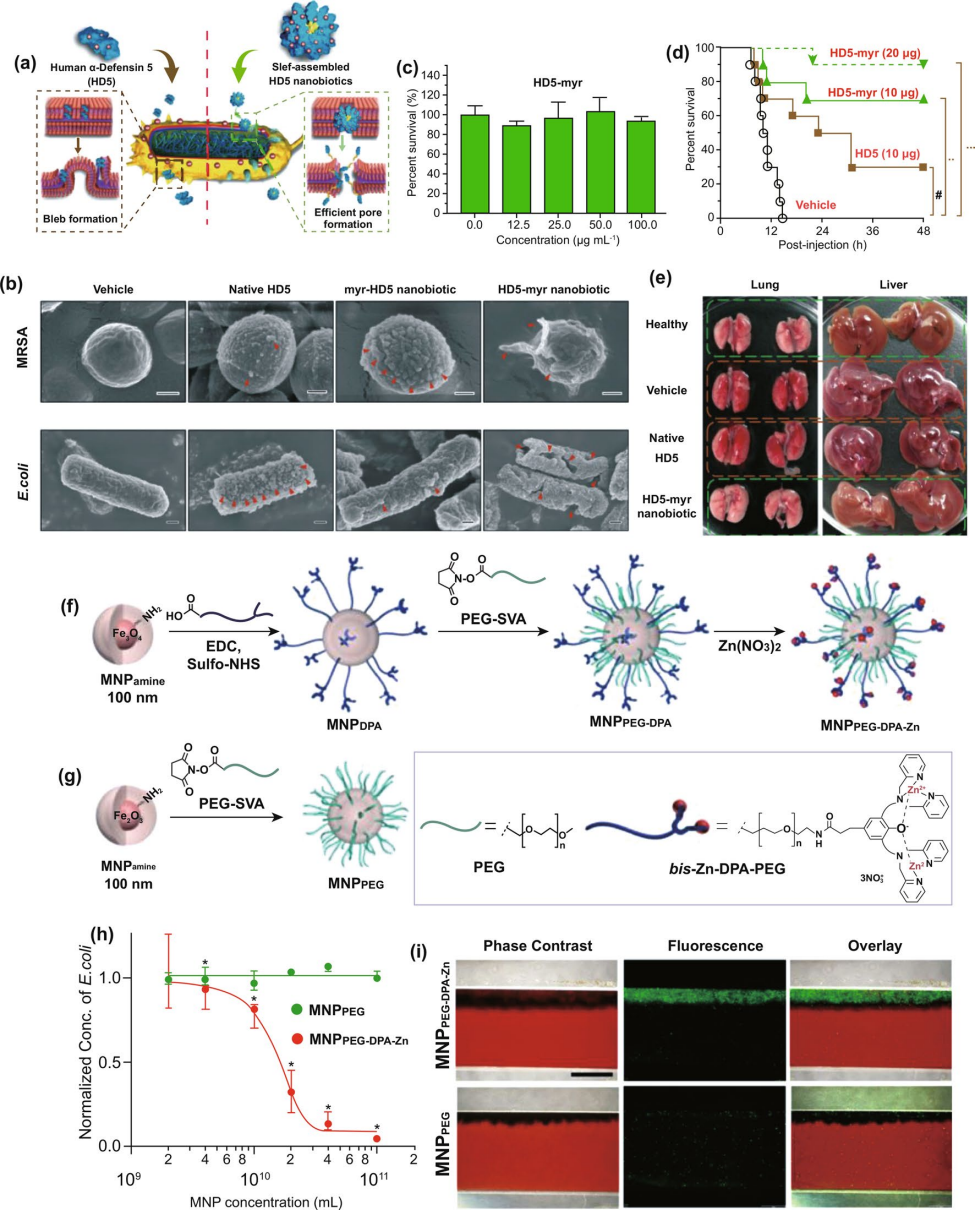
Self-assembled myristoylated HD5 as a potent nanobiotic improving sepsis outcome. **a** Schematic illustration of the bactericidal activity of HD5 and HD5 nanobiotics. **b** SEM observation of the membrane alteration of MRSA and *E. coli* after incubating with vehicle, native HD5 or nanobiotics. **c** MTT assay demonstrating the neglectable cytotoxicity of HD5-myr to RAW264.7 cells. **d** Survival rates of intraperitoneally challenged *E. coli*-induced septic mice undergoing different therapeutics including vehicle, HD5 and HD5-myr nanobiotics. **e** Photographs of lungs and livers from healthy mice and septic mice after treatment with vehicle, HD5 or HD5-myr nanobiotics. Reproduced with permission from Ref. [55]. Copyright 2018 American Chemical Society. Synthetic ligand-coated magnetic nanoparticles separating bacteria from blood with the help of a microfluidic chip. **f** Design and fabrication of MNP_PEG‑DPA‑Zn_. **g** Design and fabrication of MNP_PEG_. **h** Concentration-dependent effect of MNP_PEG‑DPA‑Zn_ and MNP_PEG_ on the separation of *E. coli* from PBS. **i** Phase contrast and fluorescence micrographs of distribution of magnetic nanoparticles and bacteria. MNP_PEG‑DPA‑Zn_ (dark in phase contrast) colocalized with *E. coli* (green fluorescence), while MNP_PEG_ accumulated without *E. coli.* Reproduced with permission from Ref. [225]. Copyright 2014 American Chemical Society

Despite their capacity to avoid drug resistance, AMPs, especially nonnatural AMPs, show potent cytotoxicity that limits their clinical applications [210–214]. Host defense peptides (HDPs), a class of endogenous AMPs with broad-spectrum activity and favorable biocompatibility, can be identified as a library of lead compounds to develop next-generation antimicrobials for the treatment of infectious diseases such as sepsis [215, 216]. HDP hepcidin, a liver-secreted AMP, can kill bacteria and modulate immune responses by regulating iron metabolism and has been employed to treat polymicrobial sepsis and acute kidney sepsis [217, 218]. Human enteric-α defensin 5 (HD5), an HDP produced by intestinal Paneth cells, can protect mice against enteric salmonellosis [219] and is thus another potential lead compound for the development of sepsis therapeutics. However, its bactericidal activity and spatial conformation are susceptible to the physiological environment (*e.g.,* ionic environment and enzyme systems), hindering its druggability [220]. Nevertheless, rational reprogramming of the structure of AMPs based on structure–activity relationships is an effective strategy to resolve this problem [221, 222]. For example, manipulating supramolecular nanotechnology to optimize the chemical structure of AMPs has obtained satisfactory efficiency in the treatment of infection-related diseases such as brain infection and sepsis [55, 223, 224]. Lei et al. [55] reported that introducing a C-terminal myristoylation of HD5 (HD5-myr) significantly enhanced the bactericidal activity of HD5 through a supramolecular nanoassembly with more potent bacterial membrane-disruption ability (Fig. 12a). Due to the supramolecular nanoassembly in aqueous solution, HD5-myr was capable of increasing the local density of positive charges to promote disruption of the bacterial membrane (Fig. 12b). In vitro antimicrobial activity demonstrated that HD5-myr exhibited broad-spectrum bactericidal effects against both gram-negative and gram-positive bacteria, whereas natural HD5 displayed limited activity due to its low charge density and poor physicochemical stability. Furthermore, HD5 is an endogenous antimicrobial peptide with favorable biocompatibility and low cytotoxicity. The MTT assay indicated that HD5-myr exhibited low cytotoxicity, similar to that of HD5 (Fig. 12c). Intraperitoneal administration of HD5-myr nanobiotics dramatically increased the survival of an *E. coli*-induced sepsis model with nonsignificant organ injury (Fig. 12d, e). The resulting HD5-myr nanobiotics possess promising potential for adapting translational applications. This study also inspired us to manipulate endogenous host defense molecules by nanomedical engineering, which might be one of the next-generation approaches to nanotherapeutics for sepsis management.

Although using potent nanobiotics to directly kill bacteria might control infections effectively, the large release of bacterial toxins from disrupted bacteria in circulation presents a potential risk of inducing subsequent inflammatory storms. Hence, therapeutics providing sufficient live bacteria-blood separation can serve as alternative or assistant strategies. To this end, Lee et al. [225] developed a magnetic nanoparticle (MNP) functionalized by a synthetic ligand, *bis*-Zn-DPA, that is capable of binding to both gram-positive and gram-negative bacteria by forming coordination bonds with anionic phospholipids on the outer membrane of bacteria. This functional nanoplatform, termed MNP_PEG-Zn-DPA_, was synthesized from commercially available aminated Fe_3_O_4_ nanoparticles with *bis*-DPA attached to the MNP surface through a PEG linker using carbodiimide chemistry following Zn^2+^ coordination to *bis*-DPA (Fig. 12f). MNPs with only PEG modification (MNP_PEG_) were also synthesized as controls (Fig. 12g). Primary effect assays demonstrated that 1.0 × 10^11^/mL MNP_PEG‑DPA‑Zn_ achieved complete separation of *E. coli* from PBS; however, MNP_PEG_ did not remove *E. coli* at each concentration (Fig. 12h). Notably, the application of MNP_PEG-Zn-DPA_ did not alter the number of red blood cells, which indicated that the recognition of MNP_PEG-Zn-DPA_ to bacteria is specific. Combined with the microfluidic system, MNP_PEG-Zn-DPA_ exhibited distinct separation of *E. coli* from bovine whole blood (Fig. 12i), suggesting a practicable translation for sepsis management.

**Fig. 13:**
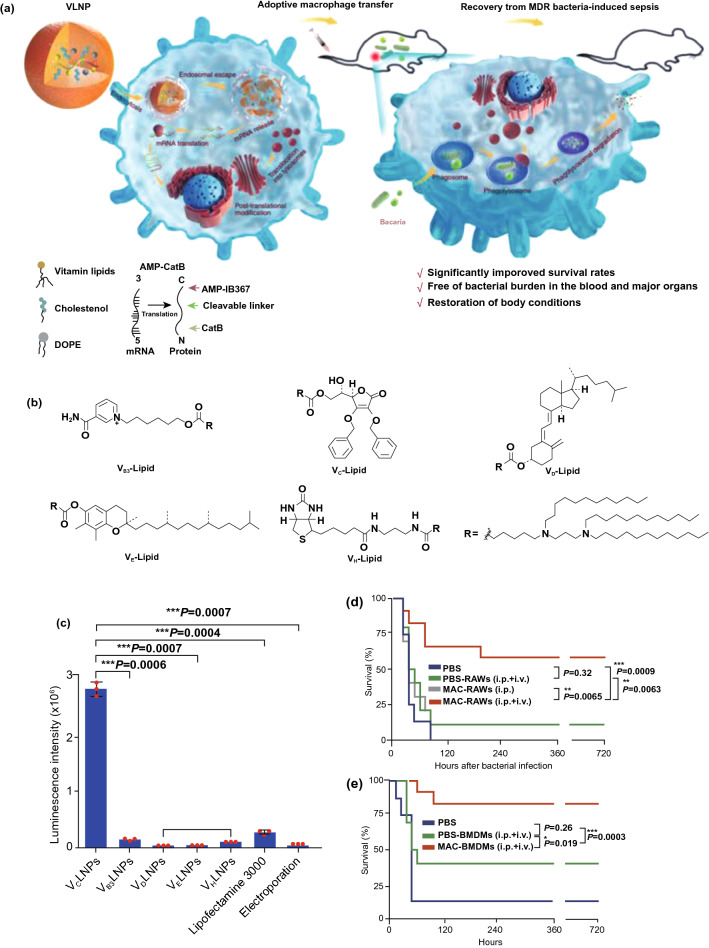
Vitamin lipid nanoparticle-guided AMP-CatB mRNA delivery restores the innate immune ability of macrophages for the treatment of multidrug-resistant bacterial sepsis complicated by immunosuppression. **a** Schematic illustration of the therapeutic mechanism of MACs mediated by VLNP delivery. **b** Chemical structures of vitamin-derived lipids, including V_B3_-lipid, V_C_-lipid, V_D_-lipid, V_E_-lipid and V_H_-lipid. **c** mRNA delivery efficiency of various VLNPs in RAW264.7 cells compared with Lipofectamine 3000 and electroporation. **d** Survival rates of bacterial inoculation-induced septic mice undergoing treatment with PBS, PBS-RAWs and MAC-RAWs. **e** Survival rates of bacterial inoculation-induced septic mice undergoing treatment with PBS, PBS-BMDMs and MAC-BMDMs. Reproduced with permission from Ref. [226]. Copyright 2020 Nature Publishing Group

Sepsis presents two obvious inflammatory statuses: the excessive inflammation phase and the subsequent immunosuppression phase [17]. In general, immunosuppression occurs in the late stage of sepsis, in which the opportunity to generate multidrug-resistant (MDR) bacteria is increased substantially due to the continuous usage of antibiotics [226]. In addition, host immune defense will undergo dysfunction, failing to eliminate bacteria. To overcome these issues, Hou et al. [226] reported that the adoptive transfer of macrophages containing antimicrobial peptide (AMP)-engineered lysosomes (MACs) could rescue MDR bacterial sepsis in mice with immunosuppression. MACs were achieved through the transfection of AMP-IB367 and cathepsin B (AMP-CatB) hybrid mRNA delivered by vitamin lipid nanoparticles (VLNPs) (Fig. 13a). VcLNPs exhibited the greatest mRNA delivery among the five VLNPs and commercial Lipofectamine 3000 as well as electroporation (Fig. 13b, c). After incubation with macrophages, the VcLNPs deliver AMP-CatB mRNA into the intramacrophage via endocytosis and endosomal escape (Fig. 13a). The released AMP-CatB mRNA would be translated into the AMP-CatB protein, in which the cathepsin B fragment is able to transport AMP-IB367 into lysosomes, thereby generating engineered lysosomes with potent bactericidal activity (Fig. 13a). Once the bacteria are captured by macrophages through the formation of phagosomes, the engineered lysosomes infuse the phagosomes to participate in bacterial elimination by the innate immune system (Fig. 13a). Although MDR bacteria could escape the attack of inherent components in phagolysosomes, particularly in the immunosuppression phase, the AMP-IB367 in phagolysosomes can directly disrupt these MDR bacteria (Fig. 13a). To test the feasibility of this approach, macrophage RAW264.7 cells and primary bone marrow-derived macrophages (BMDMs) were adopted to construct the therapeutics MAC-RAW and MAC-BMDMs using the above strategy. The results demonstrated that the administration of both MAC-RAW and MAC-BMDMs significantly improved the survival of septic mice with immunosuppression challenged by mixed MDR bacteria (*Staphylococcus aureus* and *E. coli*) (Fig. 13d, e). This fascinating strategy involves the perfect combination of nanotherapeutics and immunotherapy, which also highlights that therapy for sepsis in the immunosuppression phase should focus on restoring host immune defense, and nanotherapeutics might be sufficient to support this therapeutic concept.

### Nanotherapeutic Platforms Rescue Sepsis by Restoring Immune Homeostasis

During the pathophysiological process of sepsis, continuous infections and substantial PAMPs (e.g., endotoxins) stimulate excessive activation of the immune system with failure to restore immune homeostasis, which usually results in systemic imbalance, including inflammatory disorders, endothelial barrier injury, and coagulation abnormalities [13]. These pathological variants further progress to life-threatening multiorgan dysfunction [13]. As a consequence, effectively controlling inflammatory responses and maintaining immune homeostasis represent another essential intervention to constrain the exacerbation of sepsis. Nevertheless, there is still a lack of effective therapeutics to restore immune homeostasis [16]. The current clinically available strategies include only infection elimination, hemodynamic maintenance, and organ support; however, they fail to prevent multiorgan dysfunction [16]. The systemic administration of immunosuppressive agents or cytokine antagonists can restrict excessive inflammation, but severe side effects are inevitable. Benefiting from favorable characteristics, including the precise delivery of cargo, an inherent ability to modulate immune responses, and support for biomimetic therapies, nanotherapeutics have been widely investigated to modulate immune responses in septic models. Organic–inorganic nanotherapeutics and biomimetic nanotherapeutics exhibit considerable superiority in endotoxin elimination, anti-inflammation, and antioxidant activities, which contribute to the recovery of immune homeostasis. Other nanotherapeutics, such as protein-based delivery platforms, polymeric nanoparticles, and liposomal platforms, also contribute favorable outcomes to septic models by precise drug delivery or inherent anti-inflammatory effects. This review is meant to highlight nanotherapeutics to address sepsis and sepsis-related organ injury (Table 2); hence, publications about nanotherapeutics managing other inflammatory diseases are not included.

**Table 2:** Brief summary of nanotherapeutics improving sepsis management by controlling inflammation and modulating immunity

Classification	Nanotherapeutics	Structural organization	Target factors/pathways	Target cells	Effects	References
Organic–inorganic hybrid nanotherapeutics	Peroxidase-mimicking nanoassembly (mSPAM)	BSA-MnO_2_ NPs with coating of mSP	H_2_O_2_	Macrophage	mSPAM effectively eliminates H_2_O_2_ in macrophages, thus reducing HIF-1α level and exerting an anti-inflammatory effect	[233]
	Subnanometer gold clusters (SAuNCs)	SAuNCs were synthesized by incorporating gold atoms into dendrimers following surface engineering of alkyl motifs	LPS	Immune cell	SAuNCs bind to Lipid A domain of LPS contributing to an anti-inflammatory effect by modulating self-assembling behavior of LPS	[57]
	CeO_2_ nanoparticles	CeO_2_ nanoparticles	Peroxide	Macrophage	CeO_2_ nanoparticles attenuated oxide stress in LPS-induced sepsis through manipulating redox reactions	[231]
	Ceria-zirconia NPs	Ce_0.7_Zr_0.3_O_2_ NPs with a PEGylation shell (7CZ NPs)	Peroxide	Macrophage	7CZ NPs significantly attenuate vicious cycle of inflammatory response during sepsis by converting O_2_^−^, H_2_O_2_, and ·OH to H_2_O and O_2_	[230]
	Polymyxin B-functionalized metal alloy nanomagnet	Ultramagnetic cobalt/iron alloy NPs functionalized by polymyxin B via NHS-dPEG24-MAL linker (C/CoFe-PEG-polymyxin B)	LPS	Endothelial cells and blood cell	C/CoFe-PEG-polymyxin B NPs effectively remove endotoxins from human whole blood in an extracorporeal blood purification	[228]
Biomimetic nanotherapeutics	Macrophage-like NPs (MΦ-NPs)	A PLGA core with coating of cell membrane derived from macrophages	LPS and cytokines	Macrophage	MΦ-NPs concurrently absorb both endotoxins and proinflammatory cytokines, thus controlling the excessive inflammation in LPS/*E.coli*-induced septic mice	[234]
	Biomimetic nanosponges	PLGA nanocores coated with RBC-derived membranes	wSP of MRSA and streptolysin-O	RBC	Biomimetic nanosponges absorb and remove streptolysin-O and MRSA-originated wSP benefiting sepsis outcomes by attenuating hemolysis	[237, 238]
	Macrophage-disguised NPs	PEI-modified Fe_3_O_4_ NPs with a macrophage-derived membrane shell (Fe_3_O_4_@MMs)	LPS	Macrophage	Fe_3_O_4_@MMs are capable of capturing LPS in circulation by disguising macrophages; the LPS can then be removed by magnetic separation	[236]
	Neutrophil-derived EVs loaded with piceatannol	High-yield and scalable EVs are generated using nitrogen cavitation for targeted delivery of piceatannol (Pic-NC-EVs)	NF-κB pathway	Endothelial cell	Pic-NC-EVs effectively deliver piceatannol to endothelial cells through interacting with ICAM-1; the released piceatannol then attenuates LPS-induced ALI and sepsis by modulating the NF-κB pathway	[241]
	Leukosomes	Leukosomes are formulated by murine macrophage J774-derived membrane proteins and choline-based phospholipids as well as cholesterol	Cytokines	Macrophage	Application of leukosomes directly manipulates activated macrophages to covert to anti-inflammatory status contributing to endothelial cells homeostasis, finally prolonging survival of LPS-induced septic mice	[235]
	Mesenchymal stromal cell-derived nanovesicles (MSC-NVs)	MSC-NVs are produced by serial extrusion of mesenchymal stromal cells	IL-10	Immune cell	MSC-NVs suppress the release of cytokines into circulation, exhibiting beneficial effects in septic mice, by upregulating IL-10	[242]
Protein/peptide-based targeted nanotherapeutics	Double-chambered protein nanocage loaded with TRAP and PC-Gla (TFMG)	Self-assembling TRAP-ferritin-MMP2-PC-Gla peptide	EPCR/PAR-1 signaling	Endothelial cell	TFMG is able to control release of PG-Gla and TRAP in response to high MMP2 level in septic microenvironment, thereby activating EPCR and PAR-1 signaling that exert barrier protection and anti-inflammatory effects	[243]
	Doxorubicin (DOX)-conjugated BSA prodrug NPs	DOX was covalently conjugated into BSA with a hydrazone bond through a PEG linker, the resulted prodrug self-assembles into NPs (DOX-hyd-BSA NPs)	–	Neutrophil	DOX-hyd-BSA NPs could be specifically endocytosed by activated neutrophils via recognition of Fcγ receptors, achieving DOX intracellular delivery into neutrophils, then release DOX in an acid-sensitive manner to induce neutrophil apoptosis, which finally diminishes neutrophil transmigration and thus attenuates excessive inflammatory response in septic mice	[244]
	TNF-α siRNA-loaded helical polypeptide hybrid NPs (HNPs)	The TNF-α siRNA-loaded HNPs were fabricated through coassembly of cationic helical peptide PPABLG, TNF-α siRNA and anionic PAOBLG-MPA	TNF-α	Macrophages	PPABLG HNPs could facilitate the intramacrophage delivery of TNF-α siRNA via enhanced membrane-disruptive pores and endosomal escape, thus initiating TNF-α knockdown for anti-inflammatory therapy in LPS/D-GalN-induced hepatic sepsis	[246]
Polymeric nanotherapeutics	Cargo-less NPs	PLGA-PLA NPs	TLR pathways	Macrophage	PLGA-PLA NPs rescue LPS-induced septic mice by modulating TLR-related pathways	[249]
	Sialic acid-decorated NPs	PLGA NPs functionalized with di(α2 → 8) N-acetylneuraminic acid (α2,8 NANA-NP)	Siglecs	Hematopoietic cell	α2,8 NANA-NP specifically activates Siglecs, leading to the recruitment of SHP-1 to downregulate inflammatory mediators, significantly improving survival of LPS-induced septic mice in an IL-10-dependent manner	[250]
	Melatonin-loaded nanomedicines	Melatonin was loaded by PLGA or PLGA-PEG nanoparticles	Peroxides	–	Melatonin-loaded nanomedicines could decrease the oxide stress of sepsis models through the controlled release of melatonin	[251]
	ICAM-1 targeted nanogels loaded with dexamethasone	Lysozyme dextran nanogels with ICAM antibody functionalization were utilized to construct nanoformulation loaded with dexamethasone	–	Endothelial cells	The anti-ICAM coated nanogels could deliver dexamethasone to the lungs in a targeted manner to combat septic inflammation-induced AKI	[255]
	GLP-1 peptide nanomedicine	GLP-1 peptide was delivered by PEGylated phospholipid micelles	Cytokines	–	Using the biocompatible PEGylated phospholipid micelles to deliver GLP-1 could improve the anti-inflammatory effect of GLP-1 to battle gram-negative sepsis-induced AKI by prolonging the drug half-life	[254]
Liposomal nanotherapeutics	Interbilayer-cross-linked multilamellar vesicles loaded with sivelestat (ICMV-Sive)	Liposomes composed of DOPC and MPB were loaded with sivelestat	Neutrophil elastase and NET	Neutrophil	ICMV-Sive provides targeted delivery of sivelestat into neutrophils, significantly attenuating inflammatory injury in endotoxic shock models by decreasing NET formation by the inhibition of elastase	[256]

NPs, nanoparticles; LPS, lipopolysaccharide; mSP, mannosylated disulfide cross-linked polyethylenimine; PLGA, poly(lactic-*co*-glycolic acid); RBC, red blood cell; wSP, the whole secreted protein; MRSA, methicillin-resistant *Staphylococcus aureus*; PEI, polyethylenimine; EVs, extracellular vesicles; TRAP, thrombin receptor agonist peptide; PC-Gla, γ-carboxyglutamic acid of protein C; EPCR, endothelial protein C receptor; PAR-1, protease-activated receptor-1; BSA, bovine serum albumin; PLA, poly(lactic acid); TLR, toll-like receptor; Siglecs, sialic acid-binding immunoglobulin-like lectin; DOPC, 1,2-dioleoyl-sn-glycero-3-phosphocholine; MPNEB, 1,2-dioleoyl-sn-glycero-3-phosphoethanolamine-N-[4-(p-maleimidophenyl)butyramide] sodium salt; NET, neutrophil extracellular trap; AKI, acute lung injury

#### Organic–Inorganic Nanotherapeutics Restore Immune Homeostasis by Targeting Bacterial Endotoxins and Peroxides

Organic–inorganic hybrid nanocomposites prepared by engineering inorganic nanomaterials functionalized with organic molecules have been found to achieve meaningful applications, including the controlled delivery of diagnostic and therapeutic agents, imaging technology, and molecular separation [227]. With regard to sepsis management, the use of inorganic metal nanomaterials as solid supports can fine-tune the properties of functional organic molecules to neutralize/remove bacterial endotoxins or directly manipulate chemical reactions within the septic microenvironment to exert antioxidant effects, collectively controlling proinflammatory responses.

**Fig. 14:**
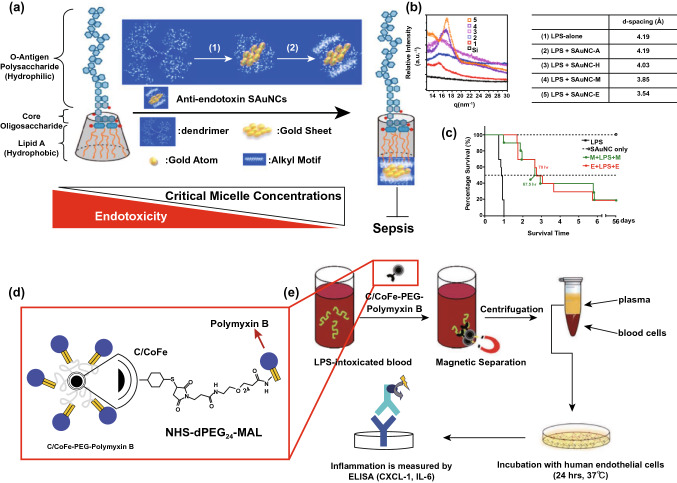
Subnanometer gold clusters protect against endotoxin-induced sepsis by adhering to lipid A. **a** Fabrication of SAuNCs and the LPS neutralization mechanisms. **b** *d*-spacing determination of lipid A in the presence of the different SAuNCs, the table summarizes the *d*-spacing distance under each condition. **c** Survival rates of LPS-induced septic undergone various treatments, the dashed line represents the half percentage survival. M and E indicate SAuNC-M and SAuNC-E, respectively. Reproduced with permission from Ref. [57]. Copyright 2018 American Chemical Society. Magnetic separation-based blood purification for endotoxin removal during sepsis management. **d** Schematic structure of C/CoFe-PEG-Polymyxin B. **e** Schematic illustration of the in vitro blood purification step using C/CoFe-PEG-Polymyxin B. Reproduced with permission from Ref. [228]. Copyright 2013 John Wiley and Sons, Inc

Liao et al. [57] introduced subnanometer gold clusters (SAuNCs) with a coating of short alkyl motifs that effectively decreased the production of proinflammatory cytokines in LPS-induced septic mice by modulating the assembly behaviors of LPS (Fig. 14a). LPS has amphiphilic features and self-assembles into various aggregates under physiological conditions, depending on the packing density of lipid A (the active site of LPS). A looser packing density is prone to trigger interaction between LPS and the TLR4-MD2 complex, which results in proinflammatory cascades. However, the packing density of LPS can be regulated by changing the intramolecular hydrocarbon chain-chain distance (*d*-spacing) of lipid A. Thus, using SAuNCs to compact *d*-spacing represents an alternative strategy to avoid excessive inflammation during sepsis progression. Gold atoms were incorporated into the dendrimer to reassemble into a cluster-like conformation, and then methyl and ethyl groups as lipid A adhesives were modified onto the SAuNC surface to generate two kinds of SAuNCs: SAuNCs-M and SAuNCs-E (Fig. 14a I). As expected, the results demonstrated that the application of SAuNCs-M and SAuNCs-E indeed compacted the *d*-spacing of LPS with a significant reduction in the critical micelle concentration (CMC) compared with those of other hydrophilic and hydrophobic SAuNCs (SAuNCs-A and SAuNCs-H) and of LPS alone (Fig. 14b). In vivo experiments indicated that both SAuNCs-M and SAuNCs-E could dramatically prolong the survival of LPS-induced septic mice by the downregulation of proinflammatory cytokines (Fig. 14c). Additionally, the removal of bacterial endotoxins from circulation represents another approach to avoid proinflammatory cascades. To this end, Herrmann et al. [228] designed polymyxin B-functionalized metal alloy nanomagnets for the rapid removal of LPS from circulation with the guidance of magnetic separation (Fig. 14d, e). The magnetic separation-based nanotherapeutic was constructed by conjugating polymyxin B onto the surface of carbon-coated cobalt/iron alloy nanomagnets through an NHS-dPEG_24_-MAL linker (C/CoFe-PEG-Polymyxin B) (Fig. 14d). After incubation with LPS-intoxicated blood, the polymyxin B motif on the nanomagnets was shown to have captured LPS, which was then removed by magnetic separation. Incorporation of the resultant blood plasma into endothelial cells did not upregulate proinflammatory cytokines such as CXCL-1 and IL-6 (Fig. 14e), suggesting that these polymyxin B-functionalized nanomagnets could potentially be applied for blood purification in sepsis management.

**Fig. 15:**
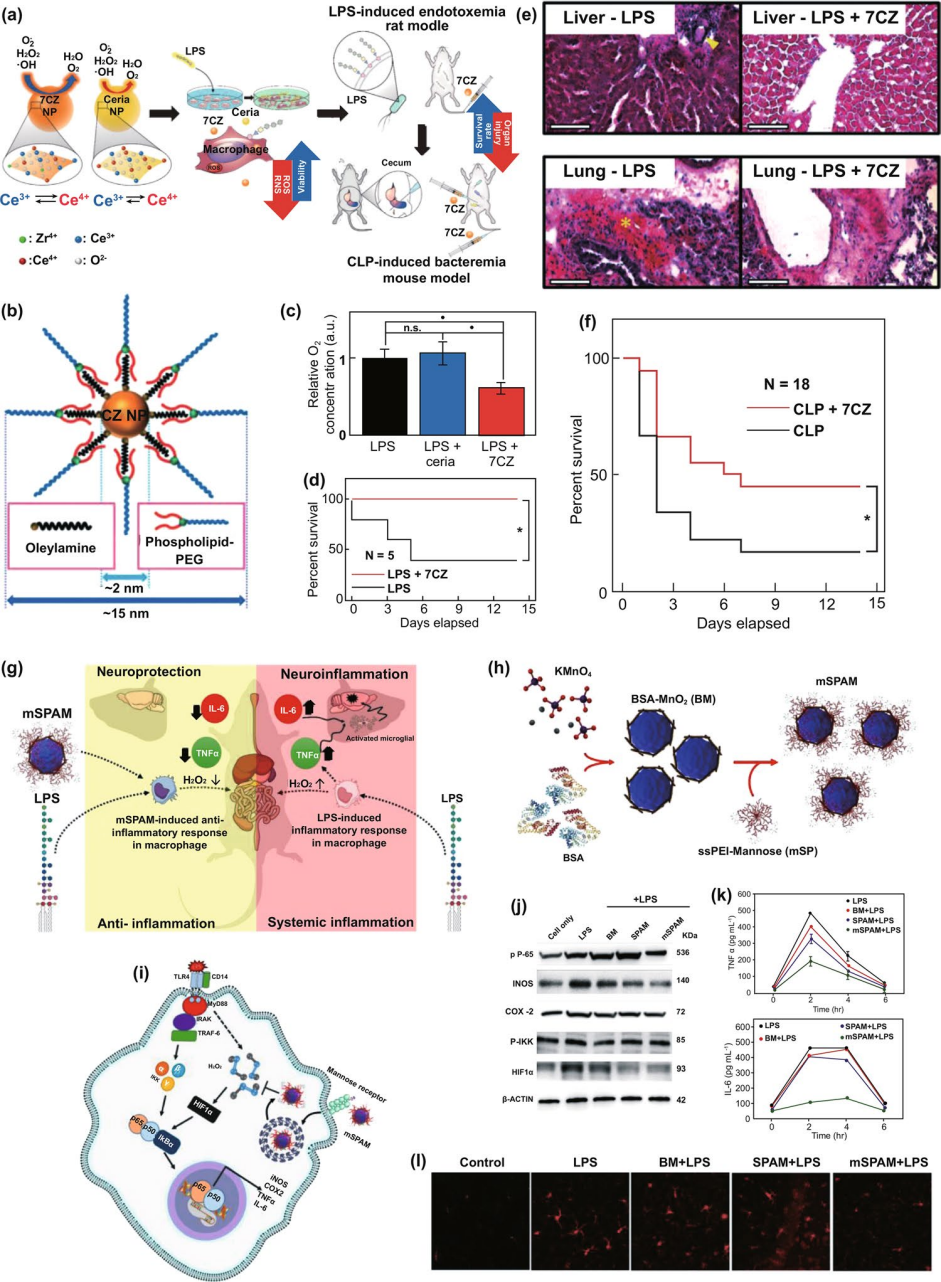
Ceria-zirconia nanoparticles with enhanced multiantioxidant effects for sepsis treatment. **a** Schematic illustration of CZ NPs as therapeutic nanomedicine for controlling in vitro inflammation and in vivo sepsis. **b** Schematic illustration of CZ NPs. **c** Relative O_2_^−^ level measurement of LPS-challenged U937 cells after treatments with vehicle, ceria NPs and 7CZ NPs. **d** Survival rates of LPS-induced septic mice undergoing therapeutics with nontreatment and 7CZ NPs. **e** Representative histopathological images of liver and lung extracted from LPS-induced septic mice with/without 7CN NP treatment. **f** Survival rates of CLP models with/without 7CZ NP therapy. Reproduced with permission from Ref. [230]. Copyright 2017 John Wiley and Sons, Inc. Peroxidase-mimicking nanoassembly with potent antioxidative effects for attenuating inflammation for the management of LPS-induced sepsis. **g** Schematic illustration of mSPAM-mediated anti-inflammatory response in both systemic inflammation and neuroinflammation. **h** Rational design and fabrication of mSPAM. **i** Schematic illustration of mSPAM nanoassembly alleviating proinflammatory response in LPS-challenged macrophages by reducing H_2_O_2_ accumulation. **j** Western blot analysis of proteins in NF-κB pathway in RAW264.7 cells treated with mSPAM nanoassembly. **k** ELISA analysis of TNF-α and IL-6 level at different time points in serum from LPS-induced mice with various treatments. **l** IBA-1 fluorescence staining of mouse brain. Reproduced with permission from Ref. [233]. Copyright 2018 American Chemical Society

The overaccumulation of reactive oxygen species (ROS) within the septic microenvironment is a major cause of multiorgan dysfunction. Inorganic metal nanomaterials (e.g., metal nanozymes) that convert peroxides (e.g., H_2_O_2_, O_2_−, and ·H_2_O) to H_2_O and O_2_ by manipulating enzyme-mimicking redox reactions [229] have been found to abolish ROS threats, contributing to favorable outcomes of sepsis. Soh et al. [230] reported that ceria (Ce)-zirconia (Zr) nanoparticles could alleviate organ injury and increase the survival rate of LPS-induced or CLP-induced septic models via enhanced antioxidation mediated by Ce^3+^-Ce^4+^ transformation (Fig. 15a). The antioxidant effect of conventional CeO_2_ nanoparticles is achieved with the chemical transformation of Ce^3+^ to Ce^4+^, which partially attenuates oxide stress and proinflammatory responses in mice with sepsis [231, 232]. However, the low reprocessing of Ce^3+^ limits the therapeutic efficiency of CeO_2_ nanoparticles and enhances their toxicity due to the large doses used. To overcome this issue, Zr^4+^ was incorporated into Ce nanoparticles to improve the reprocessing of Ce^3+^, and Ce_0.7_Zr_0.3_O_2_ nanoparticles (7CZ NPs) exhibited optimal activity to eliminate peroxides. Moreover, organic surface engineering by PEGylation was adopted to resolve the poor solubility of Ce–Zr nanoparticles (Fig. 15b). In vitro experiments indicated that LPS-challenged U937 cells treated with 7CZ NPs displayed significantly lower relative O_2_^−^ concentrations than the CeO_2_ nanoparticle group and the nontreatment group (Fig. 15c). In vivo experiments demonstrated that the intravenous administration of 7CZ NPs completely rescued LPS-induced septic mice with attenuated organ injury (Fig. 15d, e). Furthermore, the intravenous administration of 7CZ NPs could also improve the survival rate of CLP-induced polymicrobial septic models (Fig. 15f), which suggested that 7CZ NPs were also suitable for treating complex sepsis in the clinic. Rajendrakumar et al. [233] reported a mannosylated disulfide cross-linked polyethylenimine (ssPEI) (mSP)-coated bovine serum albumin (BSA)-reduced MnO_2_ (mSPAM) nanoassembly that acted as a potent H_2_O_2_ scavenger and alleviated systemic inflammation and neuroinflammation in LPS-induced septic mice (Fig. 15g). The antioxidant effect of mSPAM relied on the redox reaction mediated by MnO_2_ nanoparticles that were synthesized by reducing KMnO_4_ using BSA as a template (Fig. 15h). The mSP coating endowed the mSPAM with immune cell-targeted features, which were achieved by recognition of the mannose receptor (Fig. 15h, i). After internalization by immune cells, mSPAM converted the excess H_2_O_2_ to oxygen and water, which downregulated the expression of HIF-1α, thereby inhibiting the NF-κB inflammatory pathway (Fig. 15i). In vitro assays demonstrated that LPS-stimulated RAW264.7 macrophages treated with mSPAM displayed distinct reductions in intracellular H_2_O_2_, ROS and NO. As expected, the application of mSPAM inhibited the pP-65/NF-κB pathway, suppressing HIF-1α expression by eliminating H_2_O_2_ and thereby contributing to a reduction in iNOS and COX-2 levels (Fig. 15j). In LPS-induced septic models, therapy using mSPAM dramatically reduced proinflammatory TNF-α and IL-6 levels and improved organ protection (Fig. 15k). Furthermore, mSPAM prevented sepsis-derived neuroinflammation, as revealed by decreased infiltration of microglial cells in the brain (Fig. 15l). Consequently, employing these metal nanozymes to assist antimicrobial therapy might provide favorable organ protection for sepsis management through scavenging peroxides.

#### Cell Biomimetic Nanotherapeutics Restore Immune Homeostasis by Targeting Cytokines and Bacterial Toxins

Biomimetic nanotherapeutics that mimic cell functions or characteristics, designed by engineering cell-derived components into nanomedicines, have been shown to interfere with the pathogenesis of proinflammation, which in turn blocks the inflammatory storm during sepsis. In addition, extracellular vesicles (EVs) derived from immune cells or mesenchymal stromal cells, regarded as another biomimetic nanotherapeutic, also provide advances in sepsis management by their precise drug delivery or inherent anti-inflammatory efficiency.

**Fig. 16:**
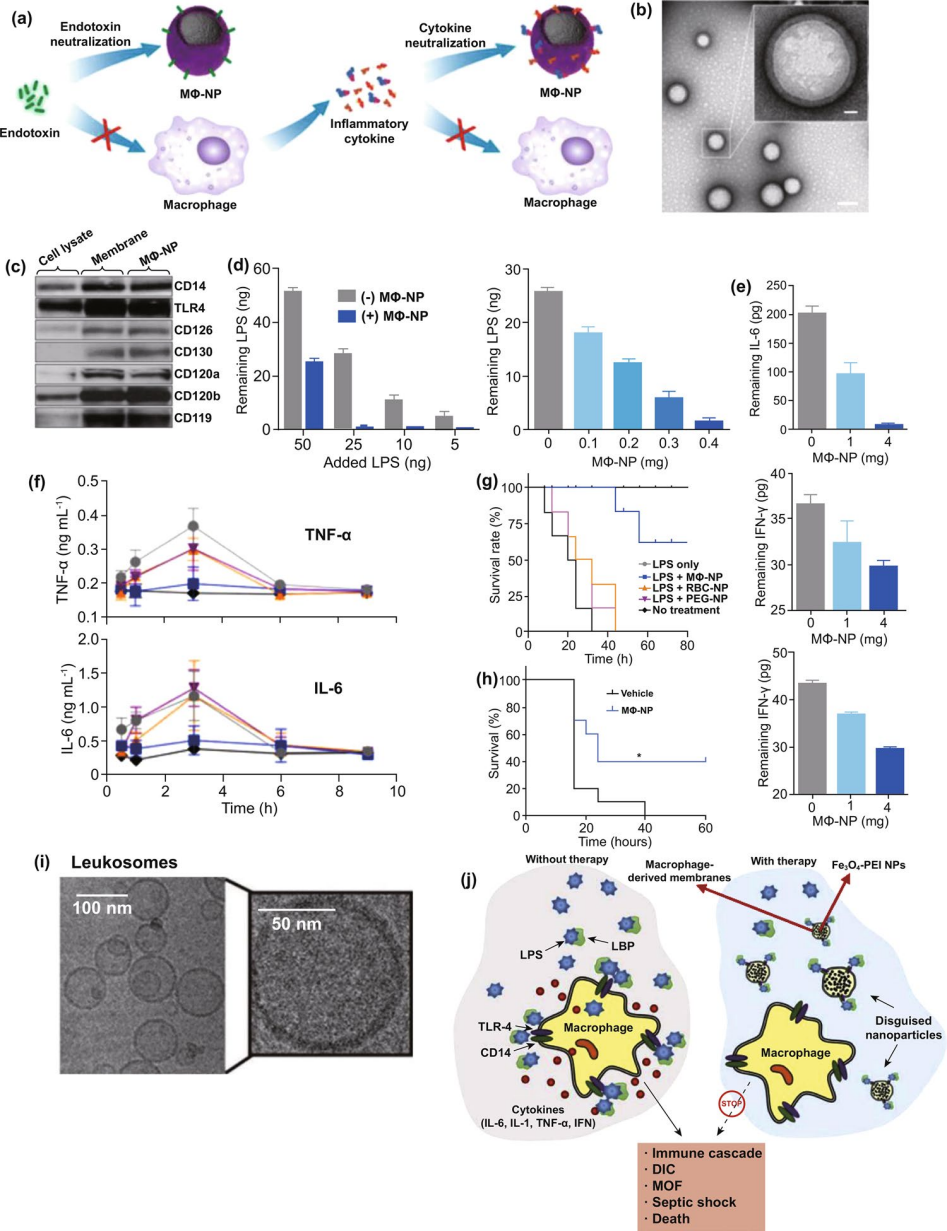
Macrophage-like nanoparticles with dual endotoxin neutralization and absorption of proinflammatory cytokine functions for sepsis management. **a** Schematic illustration of the mechanism for MΦ-NPs as a two-step process to neutralize endotoxins and proinflammatory cytokines for sepsis management. **b** TEM image of MΦ-NPs (scale bar: 100 nm; scale bar of inset image, 10 nm). **c** Determination of the expression of macrophage membrane proteins for LPS recognition and cytokine binding using western blotting. **d** Quantification of LPS removal with/without fixed MΦ-NP treatment at different amounts of added LPS (left), quantification of LPS removal with different amounts of MΦ-NP treatment at a fixed amount of added LPS (right). **e** Removal of proinflammatory cytokines, including IL-6, TNF-α, and IFN-γ, using MΦ-NPs. **f** Dynamics of proinflammatory cytokines, including TNF-α and IL-6, in plasma from LPS-induced septic mice with various treatments. **g** Survival rates of LPS-induced septic mice with various treatments. **h** Survival rates of *E. coli*-induced septic mice with/without MΦ-NPs. Reproduced with permission from Ref. [234]. Copyright 2017 National Academy of Sciences, U.S.A. **i** TEM image of leukosomes. Reproduced with permission from ref. [235]. Copyright 2019 Royal Society of Chemistry. **j** Fe_3_O_4_-PEI NPs coated with macrophage-derived membranes disguised as macrophages for LPS neutralization and protecting against sepsis. Reproduced with permission from Ref. [236]. Copyright 2019 Elsevier

Thamphiwatana et al. [234] constructed macrophage-like nanoparticles (MΦ-NPs) composed of a PLGA core and a J774 mouse macrophage-derived membrane shell (Fig. 16a, b). MΦ-NPs retaining the macrophage membrane proteins responsible for LPS recognition (CD14 and TLR4) and cytokine binding (CD126 and CD130 for IL-6 binding, CD120a and CD120b for TNF binding, and CD119 for IFN-γ) (Fig. 16c) are able to neutralize both endotoxins and proinflammatory cytokines. The in vitro incubation of MΦ-NPs with LPS in the presence of LPS-binding protein demonstrated that MΦ-NPs effectively absorbed up to 25 ng of LPS in a concentration-dependent manner (Fig. 16d). Similar results were also observed in the in vitro incubation of MΦ-NPs with proinflammatory cytokines (IL-6, TNF-α, and IFN-γ) (Fig. 16e). As expected, treatment with MΦ-NPs could prevent the occurrence of cytokine storms in LPS-induced septic mice (Fig. 16f). However, although there was a slight reduction in TNF-α and IL-6, LPS-induced septic mice treated with red blood cell-derived membrane-coated nanoparticles (RBC-NPs) and PEG-coated nanoparticles (PEG-NPs) shared similar inflammatory kinetics with LPS-only mice (Fig. 16f). More importantly, MΦ-NPs indeed rescued 60% of LPS-lethal mice, while RBC-NPs and PEG-NPs all failed to improve the survival rate (Fig. 16g). Clinical sepsis usually results from bacterial infection; hence, the authors established an *E. coli*-induced septic model to test the therapeutic efficiency of MΦ-NPs. The results demonstrated that the administration of MΦ-NPs rescued 40% of *E. coli*-induced mice with significantly lower bacterial burden and proinflammatory cytokines than that of the vehicle-treated group (Fig. 16h). A similar idea was also adopted by Molinaro et al. [235]. They reported macrophage-derived nanovesicles (termed leukosomes) that were constructed by attaching J774 mouse macrophage-derived membrane proteins onto the surface of artificial liposomes (Fig. 16i) [235]. The leukosomes specifically convert activated macrophages to anti-inflammatory status, in turn indirectly maintaining endothelial homeostasis that ultimately prolongs the survival of LPS-induced septic mice. In addition, magnetic PEI-modified Fe_3_O_4_ nanoparticles (Fe_3_O_4_-PEI) coated by macrophage-derived membranes could disguise macrophages in vivo to absorb endotoxins that were then removed through magnetic separation, thus avoiding subsequent proinflammatory cascades, disseminated intravascular coagulation (DIC), multiple organ failure (MOF), septic shock, and even death (Fig. 16j) [236].

**Fig. 17:**
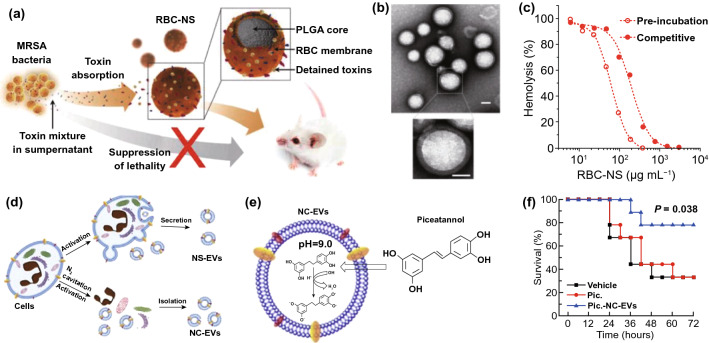
Biomimetic nanosponges coated with RBC-derived membranes protect against sepsis induced by bacterial wSP. **a** Schematic illustration of using RBC-NS to treat MRSA toxin-induced septic shock. **b** TEM images of RBC-NS. **c** Dose-dependent wSP neutralization by RBC-NS against hemolysis. Neutralization was performed in both preincubation and competitive regimens. Reproduced with permission from Ref. [238]. Copyright 2019 John Wiley and Sons, Inc. High-yield neutrophil-derived EVs produced by nitrogen cavitation for anti-inflammatory therapy. **d** Schematic illustration revealing the production of NS-EVs and NC-EVs. **e** Structure and fabrication of Pic-NC-EVs. **f** Survival rates of LPS-induced septic mice with different treatments including vehicle, Pic, or Pic-NC-EVs. Reproduced with permission Ref. [241]. Copyright 2017 Elsevier

Although RBC membrane coating did not allow nanomedicines to protect LPS-induced septic mice, RBC membrane-coated nanosponges (RBC-NS) capable of absorbing bacterial toxins (e.g., streptolysin-O) in vitro [237] have been reported by Chen et al. [238] to successfully abolish whole secreted proteins (wSP, toxin complex secreted by MRSA)-induced septic lethality by attenuating toxin-generated hemolysis (Fig. 17a). Specifically, RBC-NS consists of a PLGA core and RBC-derived membrane shell with a distinct spherical core–shell structure (Fig. 17b). In vitro assays indicated that the preincubation or competitive incubation of RBC-NS with wSP could alleviate RBC hemolysis in a concentration-dependent manner (Fig. 17c). This beneficial effect is mainly attributed to the competitive absorption of wSP preventing the disruption of RBCs. Accordingly, therapy using RBC-NS protected mice from wSP-induced sepsis in a dose-dependent and time-dependent manner, which highlighted the need for deep investigation of the pharmacodynamics of nanotherapeutics used in sepsis management to avoid negative outcomes, which could occur even when potent in vitro efficiency was observed. Extracellular vesicles (EVs), a class of nanovesicles secreted by various endogenous cells, have already been investigated as biomimetic drug delivery platforms or bioactive therapeutic nanoagents for disease therapy, including cancer, acute lung injury (ALI) and sepsis [239, 240]. Once ALI and sepsis occur, neutrophils interact with endothelial cells via the interaction between integrin β2 on neutrophils and intercellular adhesion molecule-1 (ICAM-1) on endothelial cells, contributing to subsequent activation of the NF-κB signaling pathway, which results in barrier disruption and organ injury [241]. Hence, neutrophil-derived EVs might be a suitable delivery system for the targeted treatment of ALI and sepsis. Nevertheless, the low yield of EVs limits their clinical application. Interestingly, Gao et al. [241] adopted nitrogen cavitation to achieve high-yield and scalable EVs (called NC-EVs) (Fig. 17d). The NC-EVs shared similar structures and composites with naturally secreted EVs (NS-EVs). To test the feasibility of this approach, piceatannol (Pic), an inhibitor of NF-κB, was loaded into NC-EVs (Pic-NC-EVs) to treat sepsis (Fig. 17e). The results demonstrated that intervention using Pic-NC-EVs rescued significantly more LPS-induced septic mice than intervention with Pic alone or vehicle treatment (Fig. 17f), which was attributed to the precise delivery of Pic mediated by NC-EVs. In addition to serving as drug delivery systems, EVs can directly exert anti-inflammatory effects, ameliorating sepsis outcomes. For example, mesenchymal stromal cell-derived EVs could suppress the release of proinflammatory cytokines into circulation, thereby attenuating the excessive inflammation of bacterial outer membrane vesicle-induced sepsis in an IL-10-dependent manner [242].

#### Other Nanotherapeutics Restore Immune Homeostasis by Targeting Inflammation-Related Signaling Pathways

**Fig. 18:**
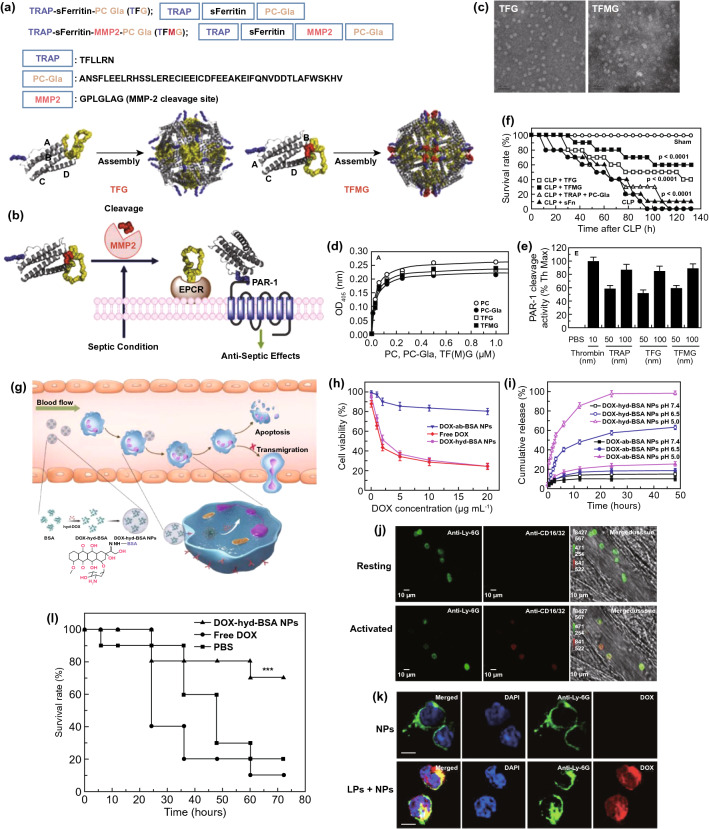
Double-chambered protein nanocage loaded with TRAP and PC-Gla for sepsis management. **a** Structure and sequence of TFG and TFMG and their self-assembly behaviors. **b** Schematic illustration of the septic microenvironment responsive property of TFMG. **c** TEM images of TFG and TFMG. **d** Affinity of PC, PC-Gla, TFG, and TFMG to sEPCR were determined by ELISA. **e** PAR-1 cleavage activity of thrombin, TRAP, TFG, and TFMG. **f** Survival rates of CLP models with various treatments. Reproduced with permission from Ref. [243]. Copyright 2015 John Wiley and Sons, Inc. DOX-conjugated protein prodrug nanoparticles increase survival in sepsis by inducing neutrophil apoptosis. **g** Schematic illustration of DOX-hyd-BSA NPs inducing apoptosis of proinflammatory neutrophils for the treatment of inflammatory diseases. **h** Cell death of neutrophil-like HL-60 cells induced by free DOX, DOX-ab-BSA NPs, or DOX-hyd-BSA NPs. **i** Cumulative release of DOX from DOX-hyd-BSA NPs or DOX-hyd-BSA NPs in PBS at pH 7.4, 6.5, or 5.0. **j** Intravital microscopy of mouse cremaster muscle venules shows that neutrophil (anti-Ly-6G, green fluorescence) activation was associated with upregulation of Fcγ receptors (anti-CD16/32, red fluorescence). **k** Confocal laser scanning microscopy (CLSM) images of blood neutrophils from normal mice or LPS-challenged mice. Both mice underwent treatment with DOX-hyd-BSA NPs at 4 h post-LPS injection. **l** Survival rates of LPS-induced septic mice after treatment with PBS, free DOX, DOX-hyd BSA NPs

**Fig. 19:**
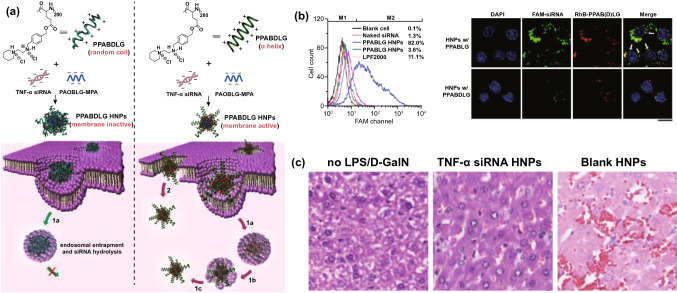
TNF-α siRNA-delivering helical polypeptide hybrid nanoparticles (HNPs) protect animals against hepatic sepsis. **a** Schematic drawing illustrating the intracellular kinetics of nonhelical PPABDLG/PAOBLG-MPA/siRNA or PPABLG/PAOBLG-MPA/siRNA HNPs, highlighting the helical conformation-dependent intracellular delivery to induce enhanced membrane disruption and endosomal escape. **b** Flow cytometric analyses (left) and CLSM (right) demonstrated the intracellular delivery capacity of HNPs to macrophage RAW264.7. **c** HE-stained liver sections from mice receiving HNPs at 50 μg of siRNA/kg. Reproduced with permission from Ref. [246]. Copyright 2016 American Chemical Society

Apart from organic–inorganic nanotherapeutics and biomimetic nanotherapeutics, some other nanomedicines involving protein-guided delivery, supramolecular polymers, and liposomes also show favorable immune modulation for sepsis treatment. Lee et al. [243] developed a double-chambered protein nanocage loaded with thrombin receptor agonist peptide (TRAP) and γ-carboxyglutamic acid of protein C (PC-Gla), in which TRAP could strongly cleave protease-activated receptor-1 (PAR-1) and PC-Gla could activate endothelial protein C receptor (EPCR). Activated EPCR/PAR-1 signaling elicits cytoprotective responses that contribute barrier protection and anti-inflammation to sepsis treatment. To construct this nanotherapeutic platform, short ferritin (sFn) was genetically engineered by inserting PC-Gla (EPCR ligand) at the C-terminus and TRAP (PAR-1 ligand) at the N-terminus to generate TRAP-ferritin-PC-Gla protein (TFG) (Fig. 18a). Furthermore, to achieve responsive release of PC-Gla and decrease steric hindrance, a matrix metalloproteinase (MMP)-2 cleavage site was inserted between sFn and the PC-Gla domain (TFMG) to enable the release of the two ligands in response to high MMP2 levels within the septic microenvironment (Fig. 18a, b). TFG and TFMG were capable of self-assembling into nanocage structures in aqueous solution (Fig. 18c). The results demonstrated that both TFG and TFMG could successfully bind to EPCR and cleave PAR-1 (Fig. 18d, e). In CLP-induced septic mice, both TFG and TFMG showed superiority in improved survival rates, and TFMG exhibited slightly higher therapeutic efficiency than TFG (Fig. 18f). The difference was due in part to MMP2-mediated controlled release endowing the two antiseptic ligands with lower steric hindrance (Fig. 18b). For cell-targeted therapies, Zhang et al. [244] reported doxorubicin (DOX)-conjugated protein prodrug nanoparticles that were engineered by conjugating DOX with BSA via a pH-sensitive hydrazone bond (DOX-hyd-BSA NPs), which specifically targeted activated neutrophils for the intracellular delivery of DOX to induce the apoptosis of activated neutrophils in order to inhibit transmigration, ultimately alleviating inflammatory responses during sepsis (Fig. 18g). For comparison, DOX was conjugated to BSA via a pH-insensitive amide bond (termed DOX-ab-BSA NPs), which could not release DOX in response to pH variance. Cumulative release assays demonstrated that DOX-hyd-BSA NPs achieved a rapid release of DOX at pH 6.5 and pH 5.0 (similar to neutrophil cytosol environments), while very little DOX was released at pH 7.4, suggesting that DOX-hyd-BSA NPs exhibited distinct stability in physiological conditions and controlled release of DOX in response to the acidic environment of neutrophils (Fig. 18h). In contrast, DOX-ab-BSA NPs did not achieve pH-dependent DOX release (Fig. 18h). Besides, DOX-hyd-BSA NPs retained the cytotoxicity of free DOX; however, the DOX-ab-BSA NPs lost their cytotoxic activity (Fig. 18i). Fcγ receptor, a surface receptor that mediates the uptake of BSA NPs by neutrophils, was found to exhibit significantly enhanced expression in activated neutrophils (Fig. 18j), which mediated the intracellular delivery of DOX-hyd-BSA NPs (Fig. 18k). While, upon resting state, the authors did not observe BSA NPs inside neutrophils (Fig. 18k). Finally, the intravenous administration of DOX-hyd-BSA NPs rescued 70% of mice undergoing LPS challenge, while the administration of free DOX did not show any protective effects (Fig. 18l), which highlighted the significant advances of the cell-targeted drug delivery system. In gene therapeutics (*e.g.,* RNA interference), a suitable gene carrier is required to guide the effective delivery of siRNA into cells to knock down the target gene [245]. To develop a gene therapy for sepsis, He et al. [246] designed an α-helical polypeptide, PPABLG, that could condense TNF-α siRNA to achieve sepsis gene immunotherapy (Fig. 19a). To obtain ultrastable nanostructures, another anionic polypeptide, PAOBLG-MPA, was incorporated to strengthen the electrostatic interaction, resulting in coassembly into PPABLG hybrid nanoparticles (HNPs) (Fig. 19a). In contrast to nonhelical PPABDLG HNPs, helical PPABLG HNPs with an amphiphilic structure could transfect more TNF-α siRNA than Lipofectamine 2000 through their membrane-disruptive capacity and endosomal escape (Fig. 19b). In an experimental model, systemic administration of helical PPABLG NHPs loaded with TNF-α siRNA significantly alleviated proinflammatory responses and rescued 50% of animals from LPS/D-GalN-induced hepatic sepsis (Fig. 19c). The literature suggests that RNA interference might be a promising anti-inflammatory gene therapy that could avoid the immune side effects caused by the traditional application of cytokine antagonists or TLR-signaling inhibitors. The rational design of adaptive nanocarriers for gene delivery is the most crucial step that determines the final therapeutic efficiency of sepsis gene therapy. Furthermore, goal-guided nanomaterials functionalized by various targeted ligands or antibodies for gene transfection may protect specific organs against proinflammatory impairment by providing clinically needed biodistribution and microenvironment delivery. Some natural products, such as quercetin, which has been identified as a scavenger of free radicals and inhibitor of proinflammatory signaling, are limited by poor oral bioavailability [247]. Zein nanoparticles combined with 2-hydroxypropyl-β-cyclodextrin could increase the oral bioavailability of quercetin by promoting drug dissolution and improving pharmacokinetic properties [247]. Using such a protein-polymer hybrid nanoplatform for quercetin delivery dramatically improved the in vivo anti-inflammatory effect in LPS-induced septic models [247].

Supramolecular nanomaterials are the most common type of nanocarriers for drug delivery [227, 248]. Intriguingly, cargo-less PLGA/PLA nanoparticles were shown to diminish inflammatory responses by directly modulating TLR-related pathways, suggesting that the inherent activity of nanomaterials should also be considered in formulating nanodrug delivery systems for sepsis management [249]. Furthermore, well-designed surface engineering of nanoparticles could also achieve surprising outcomes. Spence et al. [250] developed sialic acid-functionalized PLGA nanoparticles (named α2,8 NANA-NPs) to address acute inflammatory diseases such as sepsis because the regular display of sialic acid on the surface of nanoparticles could enhance the sialic acid-mediated oligomerization of murine sialic acid-binding immunoglobulin-like lectin-E (Siglec E), which is an important negative regulator of proinflammatory responses. As expected, compared to free sialic acid, treatment with α2,8 NANA-NPs greatly reduced TNF-α and IL-6 production in LPS-challenged macrophages. In vivo experiments indicated that the administration of α2,8 NANA-NPs completely rescued LPS-induced septic mice and prolonged the survival of CLP-induced septic mice, while the administration of free sialic acid or nonfunctionalized nanoparticles did not show protective effects. In summary, this study suggested that the appropriate display of therapeutic agents on nanomaterials might overcome the poor efficiency of free agents. Of course, the most common strategy for drug delivery is encapsulating therapeutic agents in the inner core of nanomedicines to achieve effective delivery. For example, encapsulating melatonin into the inner core of PLGA nanoparticles could significantly increase the bioavailability of melatonin for improved antioxidant effects upon controlled release [251]. Adopting generally recognized as safe (GRAS) nanomaterials to formulate anti-inflammatory agents with short half-lives and/or poor bioavailability has been investigated to prolong the anti-inflammatory efficiency in various inflammatory diseases, such as inflammatory bowel disease, arthritis, and gram-negative acute lung sepsis [252–254]. Furthermore, the surface engineering of lysozyme dextran nanogels with ICAM antibody enables targeting of the septic microenvironment, which increases the lung biodistribution of anti-inflammatory agents in animals with acute lung sepsis [255]. Although evidence indicated that the excessive formation of neutrophil extracellular traps (NETs) would worsen sepsis outcomes that could be treated by the inhibition of neutrophil elastase (NE), effectively delivering the NE inhibitor Sivelestat (Sive) into neutrophils to exert improved therapeutic efficiency is a challenge [256]. To overcome this issue, Okeke et al. [256] adopted a liposomal platform called interbilayer-cross-linked multilamellar vesicles (ICMVs) to deliver Sive (ICMV-Sive). The ICMVs were composed of DOPC and MPB, in which the anionic maleimide-headgroup of MPB would cross-link covalently to provide ultrastable liposomes, which endowed Sive with improved pharmacokinetics. Furthermore, Sive delivered by ICMVs could be specifically endocytosed by neutrophils, thus diminishing NET formation by inhibiting NE. In treating LPS-induced septic mice, ICMV-Sive showed distinct superiority compared with free Sive and ICMV. The above-mentioned results all suggest that using nanoplatforms to improve drug properties (*e.g.,* dissolution, bioavailability, biodistribution, pharmacokinetics, and pharmacodynamics) might overcome the challenges hindering therapeutic efficiency in the clinic.

### Nanotherapeutic Platforms Rescue Sepsis by Both Targeting Bacterial Infections and Restoring Immune Homeostasis

**Fig. 20:**
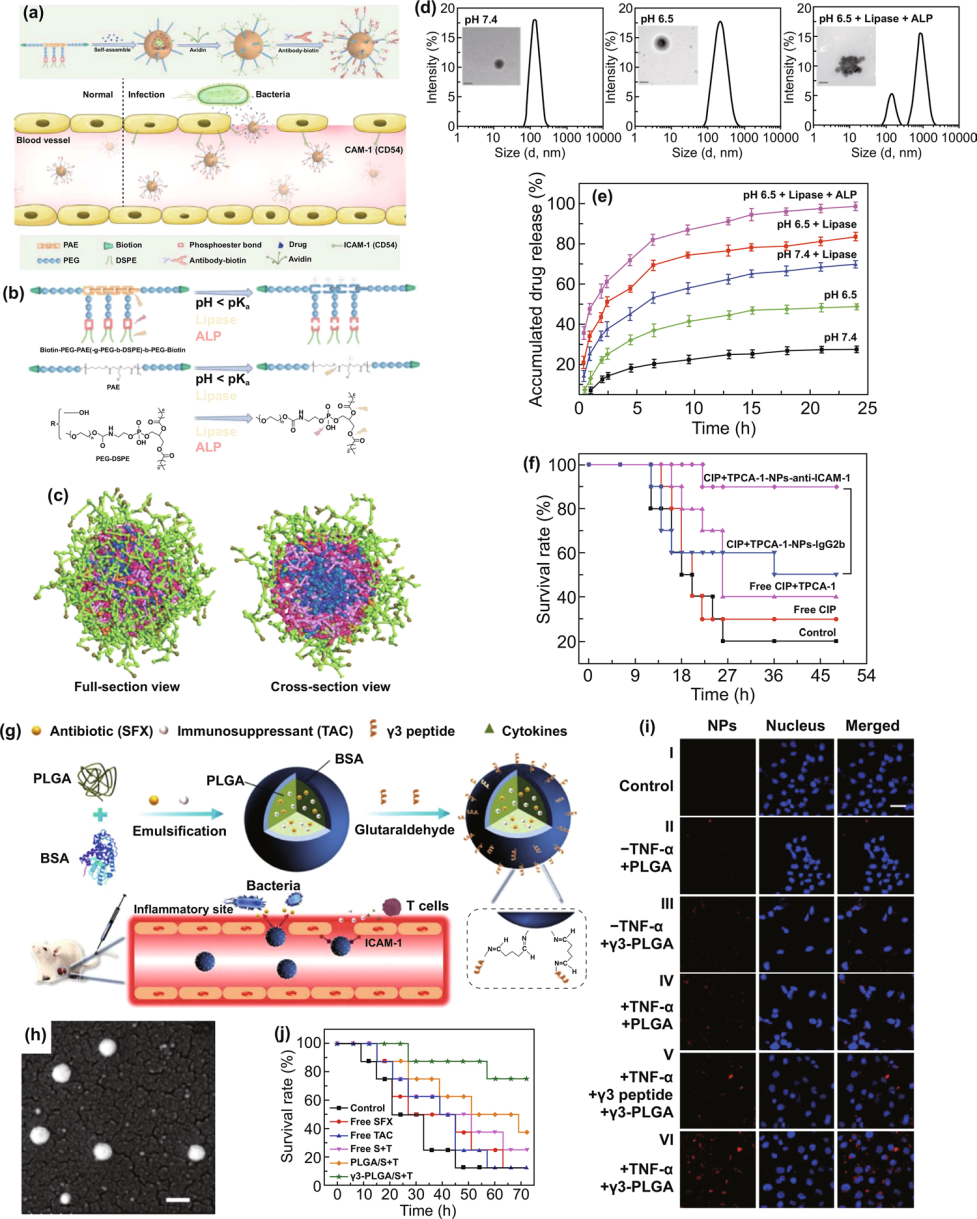
Bioresponsive nanoparticles with dual therapeutic functions targeted to infectious microenvironments for sepsis management. **a** Rational design of IME-responsive nanoparticles with well-designed surface engineering for targeted delivery of therapeutic agent at the septic microenvironment. **b** Structure and pH/enzyme-responsive natures of amphiphilic block copolymer Biotin-PEG-*b*-PAE(-*g*-PEGb-DSPE)-*b*-PEG-biotin. **c** DPD simulation of the self-assembled behaviors of the bioresponsive amphiphilic block copolymer with the drug molecule CIP, HDD (1,6-hexanediol diacrylate) units in PAE and DSPE (peach), PEG moiety (light green), biotin group (dark green), AP (3-amino-1-propanol) units in PAE (pink), CIP molecule (blue). **d** Representative DLS size and TEM images of NPs-anti-ICAM-1 incubated in PBS at pH 7.4, pH 6.5 or pH 6.5 with lipase and ALP for 2 h, scale bars, 100 nm. **e** In vitro drug release of CIP-NPs-anti-ICAM-1 in different buffers. **f** Survival rates of peritonitis-induced septic mice obtained by i.p. injection of a lethal dose of *P. aeruginosa*. At 4 h after bacterial injection, mice were treated with different drug formulations. Reproduced with permission from Ref. [257]. Copyright 2018 John Wiley and Sons, Inc. Sparfloxacin and tacrolimus-loaded polymeric nanoparticles targeting inflammation for the treatment of acute lung sepsis. **g** Schematic illustration of γ3 peptide-functionalized PLGA nanoparticles loaded with both SFX and TAC for improved treatment of lung-infected mice by targeting the inflammatory site. **h** SEM images of γ3-PLGA NPs. **i** CLSM images of interaction of PLGA NPs with HUVECs; cell nuclei (blue), PLGA NPs (red). **j** Survival rates of *P. aeruginosa*-induced septic mice after various treatments. Reproduced with permission from Ref. [258]. Copyright 2020 Elsevier

As noted in Sects. 4.1 and 4.2, infection and inflammatory disorder represent the major targets for sepsis interventions, and nanotherapeutics targeting either have shown distinct advances. Nevertheless, nanotherapeutics concurrently targeting both infection and inflammatory disorder rather than only a single target might be more useful for clinical sepsis treatment. To overcome the poor efficiency of simply combining antibiotics and anti-inflammatory agents for sepsis management, Zhang et al. [257] designed bioresponsive nanoparticles targeted to infectious microenvironments (IMEs) during sepsis, contributing to the precise codelivery and controlled release of antibiotics (ciprofloxacin, CIP) and anti-inflammatory agents (TPCA-1) (Fig. 20a). The bioresponsive nanoparticles were self-assembled by a pH/enzyme-responsive amphiphilic block copolymer that consists of biotinylated poly(ethylene glycol)-*b*-poly(β-amino ester)-*b*-poly(ethylene glycol) grafted with PEGylated lipid (biotin-PEG-*b*-PAE(-*g*-PEGb-DSPE)-*b*-PEG-biotin), in which tertiary amines and ester bonds of PAE and phosphoester bonds of PEG-DSPE could be cleaved by low pH and bacterial enzymes within IMEs (Fig. 20b). Dissipative particle dynamics (DPD) simulation demonstrated that the amphiphilic copolymers could self-assemble into shell-core structural nanomicelles (Fig. 20c). Due to the high ICAM-1 expression of vascular endothelial cells in response to infection, anti-ICAM-1 antibody was conjugated onto the surface of bioresponsive nanoparticles by biotin–avidin interaction to target IMEs. Indeed, in vitro treatment with low pH, lipase, and alkaline phosphatase (ALP) successfully induced disassembly of the bioresponsive nanoparticles and subsequent drug release (Fig. 20d, e). Furthermore, in vitro and in vivo experiments proved that the anti-ICAM-1 coating was an indispensable component enabling bioresponsive nanoparticles to achieve IME targeting. To test therapeutic potential, CIP and TPCA-1 were coloaded into the bioresponsive nanoparticles (named CIP + TPCA-1-NPs-anti-ICAM-1). The results indicated that CIP + TPCA-1-NPs-anti-ICAM-1 rescued 90% of *P. aeruginosa*-challenged septic mice, while CIP + TPCA-1-NPs-IgG2b rescued 50% of septic mice, and free CIP + TPCA-1 rescued only 40% of septic mice (Fig. 20f). Similar to survival, the application of CIP + TPCA-1-NPs-anti-ICAM-1 also exhibited considerable advantages in controlling inflammation compared with the control groups. These favorable outcomes achieved by CIP + TPCA-1-NPs-anti-ICAM-1 suggested that the precise codelivery and IME-responsive properties provided by well-designed nanotherapeutics might be an alternative strategy for drug combinations to control both infection and inflammation during sepsis. Inspired by the above study, Yang et al. [258] designed a similar nanoplatform to codeliver the antibiotic sparfloxacin (SFX) and the anti-inflammatory immunosuppressant tacrolimus (TAC) into IMEs to rescue acute lung sepsis (Fig. 20g). The nanoplatform consisting of a PLGA core and BSA shell was fabricated by an oil-in-water emulsion-based solvent-evaporation method (Fig. 20g). To provide targeting ability, ICAM-1-targeted γ3 peptide (NNQKIVNLKEKVAQLEA) was conjugated onto the surface of the PLGA-BSA nanoparticles (Fig. 20g). The resultant nanoplatform, termed γ3-PLGA NPs, showed a spherical morphology with an average size of 183.7 ± 9.4 nm and excellent biocompatibility (Fig. 15h). In vitro experiments demonstrated that γ3-PLGA NPs could specifically target activated endothelial cells rather than resting endothelial cells (Fig. 20i). Of further note, lungs from mice with acute lung infection accumulated many more γ3-PLGA NPs than other organs, also implying favorable targeting ability in vivo. In light of these advantages, SFX and TAC were coencapsulated in the hydrophobic core of γ3-PLGA NPs (γ3-PLGA/S + T NPs) to treat acute lung sepsis. The results indicated that γ3-PLGA/S + T NPs successfully rescued 75% of acute lung septic mice, which was significantly better than the controls (Fig. 20j). The bacterial burden and inflammatory responses were also controlled by γ3-PLGA/S + T NPs, contributing to the attenuation of organ injury.

Structural modification based on supramolecular chemistry has been found to enhance the bioactive activity of therapeutic agents. The antimicrobial decapeptide KSLW (KKVVFWVKFK) is a commercially available drug with dual anti-infection and vascular barrier protective functions; however, it is limited by its poor pharmacokinetics, which severely limit its activity [259]. Interestingly, lipid-PEGylation could resolve this problem. Specifically, the PEGylated phospholipid DSPE-PEG was conjugated to the N-terminus of KSLW via a Schiff base reaction, and the resultant supramolecular peptide (termed PLM-KSLW) could self-assemble into nanomicelles in aqueous solution [191]. PLM-KLSW possessed a significantly longer half-life (9.3956 h) than PEGylated KSLW (PEG-KSLW) (8.9984 h) and free KSLW (0.8626 h), which indicated that DSPE-PEGylation and PEGylation effectively improved the pharmacokinetics of KSLW. Besides, neither DSPE-PEGylation nor PEGylation affected the inherent bactericidal activity of KSLW. Intriguingly, compared with PEGylation alone, DSEP-PEGylation greatly enhanced the binding affinity between KSLW and the glycine/tyrosine-rich domain of occludin (OCLN), which dramatically improved vascular barrier integrity under septic conditions. As expected, PLM-KSLW was greatly superior to PEG-KSLW and free KSLW in preventing CLP-induced lethality in mice. The three therapeutics had nearly equal ability to eliminate bacteria, but PLM-KSLW controlled the inflammatory response better than PEG-KSLW and free KSLW, which might be attributed to the enhanced interaction between OCLN and KSLW after DSPE-PEGylation. This work suggests the potential use of nanotechnology as an efficient enhancer for the construction of promising nanotherapeutic antiseptic drugs.

Despite advances of nanoplatforms in diagnosis or therapy of sepsis, investigations involve nanoplatforms that integrate both diagnostic (e.g., in vivo imaging) and therapeutic functions (termed theranostic nanoplatforms) are rather limited. In fact, theranostic nanoplatforms have been widely used in managing various diseases, including cancer [260], neurodegenerative diseases [68], rheumatoid arthritis [261], and bacterial infection [66], etc. Inflammation and bacterial infection are the two major characteristics of sepsis. By taking advantage of the oxidative stress in inflammatory environment, theranostic agents constructed by combining an anti-inflammatory agent and a two-photon fluorophore via a ROS sensitive bond could accumulate in inflammatory sites with the delivery by polymeric NPs whose biodistribution can be tracked by two-photon aggregation-induced emission (AIE) imaging, and subsequently release therapeutic agents for constraining inflammation [262]. This theranostic nanoplatform has been proven to exhibit favorable application in arthritis, atherosclerosis, and LPS-induced acute lung sepsis [262]. Given high expression of vascular cell adhesion molecule-1 (VCAM-1) in activated endothelium, Fuior and coworkers developed a theranostic nanoemulsions that were attached by a peptide ligand of VCAM-1 for conferring targeting property [263]. Naringenin and indocyanine green (ICG) were coincorporated into the nanoemulsions, as therapeutic agent and imaging probe, respectively. In a mouse model of LPS-induced inflammation, the resultant theranostic nanoemulsions could selectively accumulate in endothelium-rich organs (e.g., heart and lung) via a synergistic delivery mediated by passive and active targeting, which were clearly visible under the NIR imaging [263]. To achieve bacterial theranostics, Mao and coworkers fabricated a D-AzAla@MIL-100 NPs consist of MOF MIL-100 (Fe) as carrier, D-AzAla as bacterial labeling agent, and pluronic F127 as stabilizer [264]. After in vivo administration, D-AzAla@MIL-100 NPs accumulated in infectious environment via EPR-like effect, and the MIL-100 was subsequently selectively damaged by H_2_O_2_, which allowed robust release of D-AzAla in infectious region. These free D-AzAla could bind to the peptidoglycan of bacteria, thus achieving sufficient expression of azide groups on bacterial surface. Afterward, administration of photosensitizers-loaded AIE NPs led to the bacterial metabolic labeling through a click reaction, which allowed the in vivo tracking of target bacteria and subsequent PDT-mediated bactericidal activity.

The aforementioned nanoplatforms show favorable applications in inflammatory and bacterial theranostics and thus might be promising candidates for sepsis theranostics. Nevertheless, due to the specificity of sepsis, feasibility of these inflammatory or bacterial theranostic nanoplatforms in sepsis management should be in-depth evaluated in septic models including polymicrobial sepsis, PAMAs-induced inflammatory sepsis, and CLP models. Of further note, diagnosis of sepsis usually requires characterization of multiple biomarkers rather than single markers, and its therapeutic strategies should adopt multitarget intervention rather than single-target therapy. Consequently, from our perspective, future development of nanoplatforms for sepsis management should consider how to integrate multibiomarker diagnostics and multitarget therapies into a single nanoplatforms and achieve multibiomarker imaging (e.g., different fluorescent color for different biomarkers)/quantification and on-demand release of different therapeutics. A sophisticated nanoplatform developed by Shi and coworkers might achieve multimodal theranostics for sepsis if further improved in the future [265]. They synthesized a telodendrimer (TD) nanotrap (NT) capable of selectively capturing PAMPs/DAMPs (e.g., LPS and cytokines) via multivalent, hybrid and synergistic interactions. By rational controlling charge and hydrophobicity of TD, TD could specifically bind to different PAMPs/DAMPs. The TD-NTs were encapsulated by a size-exclusive hydrogel resins which allows septic molecules to enter while other molecules (*e.g.,* HSA and IgG) cannot. Administration of TD-NTs resin alone rescued 50% of CLP-induced septic mice, whereas CLP-induced septic mice could completely survive when combining TD-NTs resin and antibiotic treatment. Though this study only proved the therapeutic functions of TD, we theorize TD also is a promising material for multimodal diagnosis through quantifying the types and amounts of PAMPs/DAMPs in septic patients.

## Summary and Outlook

This review provides a general description of sepsis that mainly involves clinical definition, pathogenesis and therapeutics and then deeply elucidates the recent advances of nanoplatforms in managing sepsis. With the continuous clarification of pathogenetic mechanisms, the theranostic principles and strategies for treating sepsis have gradually been improved. Furthermore, introducing nanotechnology into preclinical research on theranostics of sepsis has also achieved significant advances, offering promise for clinical usage. Nevertheless, there are still many challenges that require collaboration among pharmaceutical chemists, material scientists, biochemists, and clinicians to resolve. Some important problems listed below need to be further explored and will serve as a roadmap to develop future beneficial strategies for sepsis management.

(i) Despite significant advances in preclinical management, clinical translation and application of nanoplatforms remain a challenge. The construction of the nanoarchitectures requires the utilization of diverse nanomaterials, such as supramolecular nanomaterials, organism-originated biomaterials, metal nanoparticles, and organic–inorganic nanocomposites. However, most of these materials have not been approved by the FDA as pharmaceutically acceptable adjuvants. It is crucial for pharmaceutical materials to focus instead on biocompatibility and biodegradability, absorption, distribution, metabolism, and elimination (ADME) processes, in vivo drug-material interactions, the cross-influence between nanomaterials and body systems/organs, toxicology and side effects. Although mounting evidence has gradually illuminated the interactions between nanomaterials and body systems, especially the vasculature and respiratory system [266, 267], more efforts are urgently needed to elucidate the detailed mechanisms and the influence of nanomaterials on other body systems, which will contribute to mapping the structure-bioeffects relationship, thus guiding the precision optimization of nanomaterials for in vivo applications. Only by fully clarifying the above-mentioned elements or parameters, these fascinating nanoplatforms can be advanced toward use in clinical practice. In this regard, in-depth understanding structure–activity relationship (SAR) of nanomaterials is of particular importance, which shall aid precision design of nanomaterials to minimize unwanted responses and maintain superiorities. Usually, biological effects and profiles such as circulation time, metabolic pathways, biodistribution, biodegradation, and release behaviors, mainly determined by the biophysicochemical characteristics of NPs, including size, composition, shape, charge, surface chemistry, hydrophilic/hydrophobic features, protein corona, and assembly modes, etc. Passive distribution of NPs can be modulated by size, shape, and/or charge; adjusting proper surface chemistry contributes to active targeting behaviors; personalized drug encapsulation and release can be achieved via tuning hydrophobicity and/or assembly modes. Only balancing every properties of NPs no matter for inherent natures or attached functions, we can finally obtain the most medically/pharmacologically acceptable nanoplatforms, which, however, needs more efforts to explore and/or summarize SAR of nanomaterials.
(ii) Although the pathogenetic mechanisms and clinical manifestations have been gradually elucidated, effective drugs for treating sepsis remain limited. Currently, only antibiotic usage, hemodynamic maintenance, and organ support are clinically available. However, these strategies fail to prevent the occurrence of multiorgan dysfunction and inflammatory cascades. In addition, high heterogenicity and complex pathogenesis require multipathway therapeutics rather than single-factor therapeutics throughout the treatment process. Fortunately, some molecular signaling pathways were found to modulate the pathogenesis of sepsis and consequently could be considered valuable drug targets for the development of novel therapeutics. Agonists or antagonists of these signaling pathways might be potential drug candidates with promising clinical utility in multipathway therapeutics. However, the poor solubility and pharmacokinetics of these candidate compounds limits their clinical translation. Notably, nanotechnology has already been found to resolve such problems through introducing amphiphilic nanocarriers or modulating crystal forms. Hence, we theorized that combining drug discovery and nanomedicine should drive the future clinical translation of therapeutic candidates.
(iii) The diagnosis of sepsis remains challenging due to its heterogenicity and complex pathogenesis and depends mainly on the characterization of infection, inflammatory status, and organ injury. Currently, the severity of organ injury can be revealed by the SOFA scoring system in clinical practice, while the infection and inflammatory status are usually indicated by the detection of corresponding biomarkers. Nanomaterial-inspired nanodiagnostic platforms contribute accurate and rapid detection of sepsis-associated biomarkers such as live bacteria, CRP, PCT, and cytokines, satisfying the needs of point-of-care diagnosis for timely warning of sepsis progression. Nevertheless, these biomarkers are not specific for sepsis characterization due to their positive signaling in other infectious and/or inflammatory diseases. Consequently, in-depth research to discover sepsis-specific biomarkers represents the most important step for improving early warning. Of further note, developing corresponding nanodiagnostic platforms to reinforce the sensitivity of novel biomarkers might be meaningful. In addition, real-time monitoring of the septic microenvironment (*e.g.,* immune status) helps clinicians to judge disease progress and the need for therapeutics and can be achieved by analyzing functions and signaling molecules at the single-cell level. Thus, integrating single-cell sequencing technology into nanodiagnostic platforms may reveal alterations in the septic microenvironment (*e.g.,* cytokines, signaling pathways and immune cells) in a real-time, accurate, and rapid manner.
(iv) Nanotherapeutic platforms targeting infections and/or immune disorders have achieved favorable therapeutic efficiency in sepsis. However, manipulating nanomedicine to potentiate the activity of antibiotics or to restore immune homeostasis by organic–inorganic nanotherapeutics, cell biomimetic nanotherapeutics or other strategies is insufficient for multipathway therapy, making it difficult for current nanotherapeutics to address clinical sepsis. To date, investigations to develop multipathway nanotherapeutics remain rare. Current dual-function nanotherapeutics simply encapsulate commercial antibiotics and immunosuppressants into nanocarriers, contributing targeted anti-infection and anti-inflammation effects to sepsis therapy. Despite relatively favorable outcomes, their ability to manage different sepsis statuses represents a huge challenge. Hence, designing septic status-responsive nanotherapeutics to treat different inflammatory and infectious phases through the controlled release of corresponding agents might be more interesting and reliable. Furthermore, organ dysfunction is a life-threatening element in sepsis mortality. Accordingly, in addition to antibiotics and immune modulators, therapeutic agents to alleviate organ dysfunction should also be incorporated into nanotherapeutics. For example, some growth factors could be codelivered by nanoplatforms to assist the regeneration of injured organs; the function of mitochondrial resuscitation could also be introduced into nanotherapeutic design to restore energy metabolism and thereby recover organ functions. Sepsis management is still a challenge in critical care medicine. Although pathogenetic research and nanoplatforms have achieved significant advances in the preclinical therapy of sepsis, there are many ongoing challenges demanding more collaborative efforts with multidisciplinary cross-linking among critical care medicine, medicinal chemistry, material engineering, and immunology.
